# Direct and Efficient C(sp^3^)–H Functionalization of *N*-Acyl/Sulfonyl Tetrahydroisoquinolines (THIQs) With Electron-Rich Nucleophiles via 2,3-Dichloro-5,6-Dicyano-1,4-Benzoquinone (DDQ) Oxidation

**DOI:** 10.3389/fchem.2020.00629

**Published:** 2020-07-29

**Authors:** Heesun Yu, Hyoungsu Kim, Seung-Hoon Baek, Dongjoo Lee

**Affiliations:** Research Institute of Pharmaceutical Science and Technology (RIPST), College of Pharmacy, Ajou University, Suwon, South Korea

**Keywords:** tetrahydroisoquinoline, oxidation, DDQ, electron-rich, natural products

## Abstract

A highly efficient metal-free oxidative direct C(sp^3^)–H functionalization of *N*-acyl/sulfonyl 1,2,3,4-tetrahydroisoquinolines (THIQs) with a wide range of electron-rich nucleophiles was accomplished under mild conditions through oxidation with DDQ and subsequent trapping of the resulting reactive and stable *N*-acyl/sulfonyl iminium ions. The synthetic utility of this method was illustrated by a concise and efficient total synthesis of (±)-benzo[*a*]quinolizidine (**10**) in 3 steps from the known *N*-Cbz 1,2,3,4-THIQ **4b**.

## Introduction

**Figure 1:**
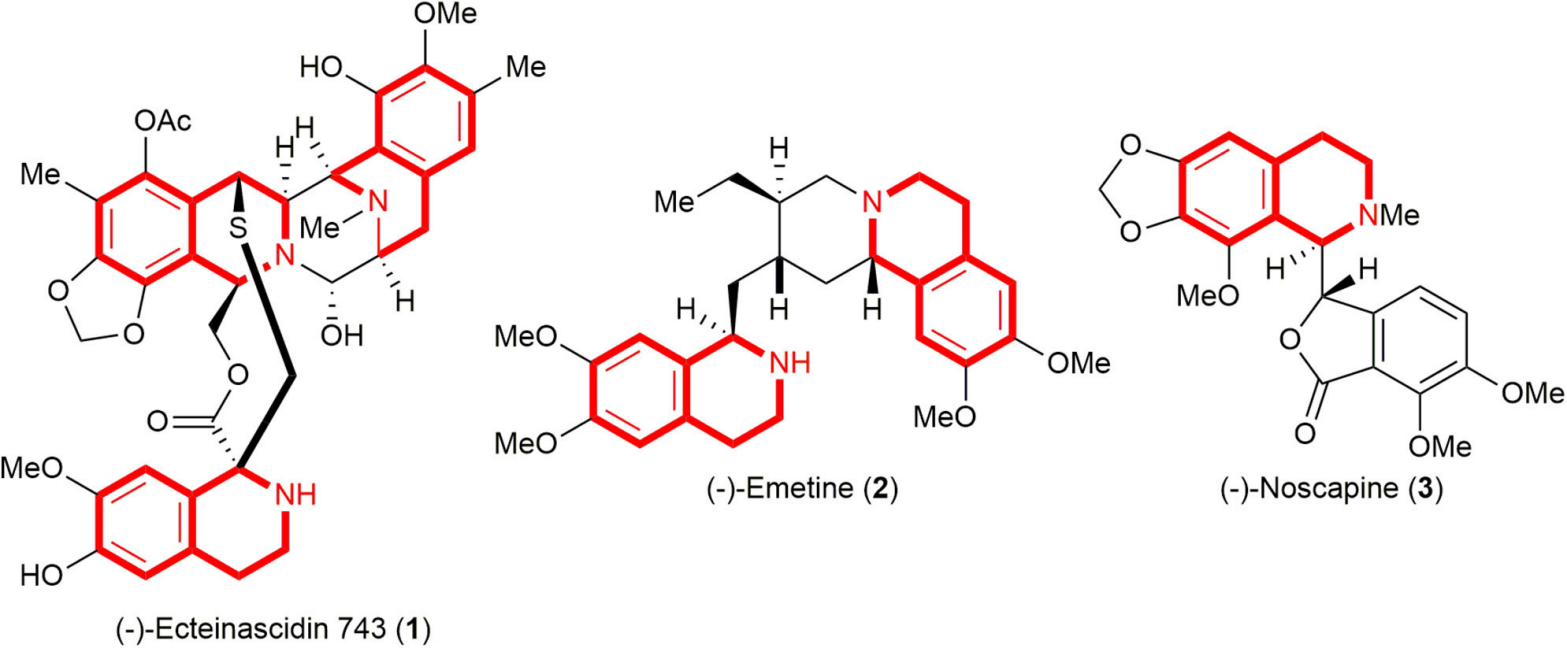
Selected biologically active natural products embodying C(1)-substituted 1,2,3,4-tetrahydroisoquinoline (THIQ) subunit.

C(1)-Substituted 1,2,3,4-tetrahydroisoquinolines (THIQs) constitute an important family of biologically active alkaloids, and their derivatives are found as major structural motifs in a wide range of natural products as well as medicines such as (–)-ecteinascidin 743 (Yondelis®, **1**, anti-tumor activity) (Rinehart, 2000), (–)-emetine (**2**, treatment of amoebiasis and amebic dysentery) (Akinboye and Bakare, 2011), and (–)-noscapine (**3**,anti-tussive agent) (Segal et al., 1957) (Figure 1). Not surprisingly, natural and synthetic C(1)-substituted 1,2,3,4-THIQs have attracted much interest from synthetic organic as well as medicinal chemists due to their interesting structural features, in conjunction with a diverse range of biological activities (Bentley, 2001; Scott and Williams, 2002; Chrzanowska and Rozwadowska, 2004), and the development of a new and efficient strategy toward the construction of the C(1)-substituted 1,2,3,4-THIQs still remains imperative.

**Scheme 1:**
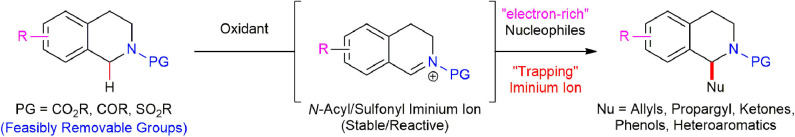
Proposed strategy for oxidative C(sp^3^)–H functionalization of *N*-acyl/sulfonyl 1,2,3,4-THIQs with electron-rich nucleophiles.

During our investigation of the scope and limitations of using a variety of nucleophiles in the oxidative direct C(sp^3^)–H functionalization of *N*-acyl/sulfonyl 1,2,3,4-THIQs (Kim et al., 2018), we recognized that a variety of structurally and electronically different nucleophiles were employed in the majority of reported examples such as styrenes (Richter et al., 2012), terminal alkynes (Su et al., 2011; Freeman et al., 2012; Yu et al., 2013; Sun et al., 2015), nitroalkanes (Tsang and Todd, 2009; Hari and König, 2011; Su et al., 2011; Dhineshkumar et al., 2013; Nobuta et al., 2013), dialkyl malonate (Dubs et al., 2008; Hari and König, 2011), malonitrile (Su et al., 2011), nitrile (Murahashi et al., 2003, 2005; Yan et al., 2014), aldehydes (Xie et al., 2016), α,β-unsaturated aldehydes (Zhang et al., 2012), ketones (Shen et al., 2009; Sud et al., 2009; Alagiri et al., 2012a,b; Chen et al., 2014), α,β-unsaturated γ-butyrolactam (Ma et al., 2014), coumarins (Alagiri et al., 2012a,b; Dhineshkumar et al., 2013), aryl boronic acids (Baslé and Li, 2008), aryl boronates (Liu et al., 2015), organotrifluoroboronates (Xie et al., 2014), phosphonates (Hari and König, 2011; Alagiri et al., 2012a,b; Wang et al., 2012), or difluoramide (Chen et al., 2015). Although sporadic examples were reported on the use of electron-rich aromatic nucleophiles such as indole (Alagiri et al., 2011; Ghobrial et al., 2011; Su et al., 2011; Dhineshkumar et al., 2013) and phenols (Dhineshkumar et al., 2013) in this research area, there has been no practical and general method for oxidative direct C(sp^3^)–H functionalization of 1,2,3,4-THIQs with electron-rich nucleophiles which are labile to oxidation such as organostannes, silyl enol ethers, or other aromatic rings bearing electron-donating substituents. We postulated that the use of such electron-rich nucleophiles was limited in this area, presumably since they are rapidly oxidized and lose their nucleophilicity under oxidative conditions. In addition, silyl enol ethers or ketene silyl acetals are very unstable under harsh reaction conditions such as a high temperature and long reaction time that most transition metal-catalyzed oxidative direct C(sp^3^)–H functionalization of 1,2,3,4-THIQs required to proceed to completion. We envisaged that this problem could be circumvented through the direct oxidation of *N*-protected 1,2,3,4-THIQs with a proper oxidant in the absence of moisture first, thereby leading to a high-yielding *in situ* reactive and stable iminium ion along with the consumption of the oxidant, then subsequent trapping of the resultant iminium ion with electron-rich nucleophiles, which will afford the corresponding *N*-protected C(1)-substituted 1,2,3,4-THIQs avoiding the oxidation of electron-rich nucleophiles (Scheme 1).

The majority of oxidative functionalization reactions widely employed an aryl group as the activating and protecting group for 1,2,3,4-THIQs (Li, 2009; Scheuermann, 2010; Yoo and Li, 2010; Klussmann and Sureshkumar, 2011; Yeung and Dong, 2011; Rohlmann and Mancheño, 2013), since the aryl group on the nitrogen atom activates the C(sp^3^)–H bond at the C(1)-position of 1,2,3,4-THIQs and stabilizes the resulting iminium ion intermediate. Although Todd and co-workers recently identified that 4-methoxyphenyl (PMP) group is a removable protecting group in the oxidative direct C(sp^3^)–H functionalization (Tsang et al., 2013), it still proves to be problematic to remove the aryl protecting group from the nitrogen atom in the presence of other functional groups, which significantly limits the synthetic utility of oxidative functionalization of *N*-aryl 1,2,3,4-tetrahydroisoquinolines. For instance, the phenyl protecting group from amines was removed under harsh reaction conditions where only a small set of organic compounds could be tolerated (Girard et al., 2004, 2005; Girard and Hurvois, 2007). Therefore, use of easily removable *N*-acyl or *N*-sulfonyl groups on the nitrogen atom of 1,2,3,4-THIQs in place of the aryl ones would provide an attractive solution for enhancing the scope and synthetic utility of the direct C(sp^3^)–H functionalization of 1,2,3,4-THIQs through generating a more reactive *N*-acyl/sulfonyl iminium ion intermediate that can react with a broader range of nucleophiles.

Considering that 1,2,3,4-THIQ motifs are core units found in a multitude of pharmacologically active natural products and medicines, the development of an operationally convenient and practical method to introduce a wide range of nucleophiles is still a worthwhile project to pursue. Herein we wish to report a new direct metal-free direct C(sp^3^)–H functionalization of *N*-acyl/sulfonyl 1,2,3,4-THIQs with a variety of electron-rich nucleophiles via 2,3-dichloro-5,6-dicyano-1,4-benzoquinone (DDQ) oxidation under ambient conditions.

## Results and Discussion

### Initial Attempt and Optimization of the Reaction Conditions

**Scheme 2:**
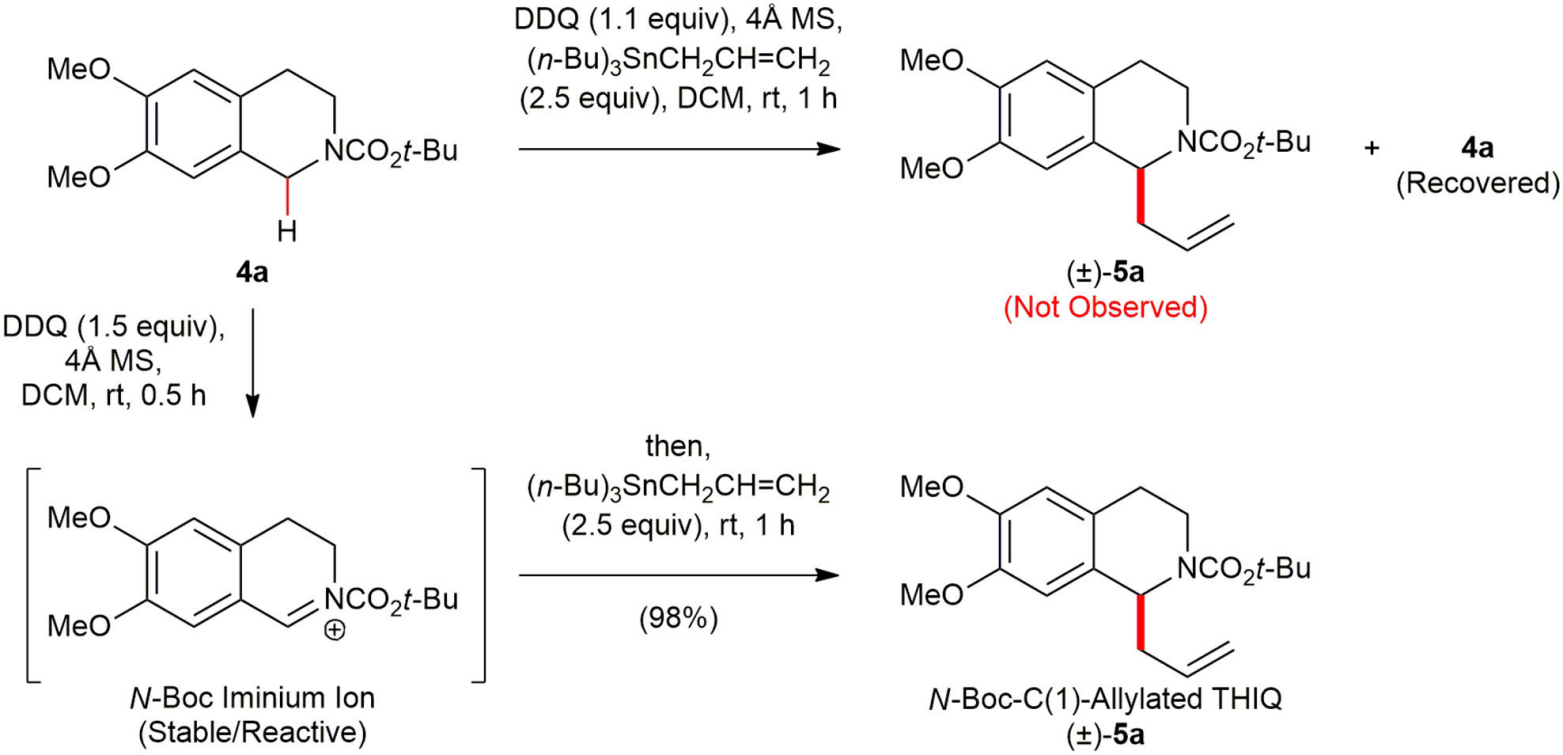
DDQ-promoted C(1)-allylation of *N*-Boc 1,2,3,4-THIQ **4a** with an electron-rich allyltributylstannane.

At the outset of our studies, we examined the C(1)-allylation of *N*-Boc 1,2,3,4-THIQ **4a** (Hickin et al., 2014), which is ubiquitous structural frameworks in numerous pharmacologically active THIQ natural products, as a model substrate to test the viability of the envisioned direct metal-free C(sp^3^)–H functionalization. The allyl moiety is exceptionally versatile and synthetically useful in that this functional group offers a wealth of opportunities to further functionalization (Denmark and Fu, 2003). Although Wang and co-workers (Yan et al., 2015) recently reported the use of allyltrimethylsilane (Me_3_SiCH_2_CH=CH_2_) as the nucleophile in direct oxidative C(1)-allylation of *N*-acyl/*N*-sulfonyl 1,2,3,4-THIQs employing 2,2,6,6-tetramethylpiperidine-1-oxoammonium tetrafluoroborate (T^+^${\text{BF}}_{4}^{-}$), success of such a direct oxidative transformation with an electron-rich allyltrialkylstannane was not yet to be proven, presumably, due to their high propensity of oxidation in the presence of oxidizing agents. It is difficult to generate *N*-acyl or *N*-sulfonyl iminium ion intermediates with commonly used transition metal catalysts or non-metal organic oxidants (Luo et al., 2020). Therefore, a judicious selection of oxidant is critical. We selected 1,2-dichloro-5,6-dicyano-1,4-benzoquinone (DDQ) (Walker and Hiebert, 1967; Fu and Harvey, 1978; Wendlandt and Stahl, 2015) since it is inexpensive and stable organic solid that is conveniently handled under ambient conditions, and permits mild and more practical reaction conditions. To test the compatibility of allyltributylstannane ((*n*-Bu)_3_SnCH_2_CH=CH_2_) in the presence of DDQ, DDQ (1.1 equiv) was added to a mixture of **4a** (1.0 equiv) and (*n*-Bu)_3_SnCH_2_CH=CH_2_ (2.5 equiv) in the presence of 4Å MS in DCM, and the reaction mixture was stirred for 1 h at ambient temperature (Scheme 2). However, the desired C(1)-allylated *N*-Boc 1,2,3,4-THIQ (±)-**5a** was not obtained, but most of **4a** was recovered, presumably due to faster oxidation of electron-rich nucleophile (*n*-Bu)_3_SnCH_2_CH=CH_2_ than **4a**. Pleasingly, treatment of **4a** with DDQ (1.1 equiv) as an oxidant in the presence of 4Å MS in DCM at room temperature for 30 min, thereby leading *in situ* high yield of the reactive *N*-Boc iminium ion along with consumption of the oxidant. The subsequent addition of (*n*-Bu)_3_SnCH_2_CH=CH_2_ (2.5 equiv) afforded the desired (±)–**5a** in excellent yield (98%). Molecular sieves (4Å) was added to eliminate moisture that might be present in the reaction mixture and the reactivity of the *N*-Boc iminium ion lasted for several hours at room temperature under argon atmosphere. To the best of our knowledge, such a DDQ-mediated direct functionalization of C(sp^3^)–H functionalization of *N*-Boc 1,2,3,4-THIQ with electron-rich (*n*-Bu)_3_SnCH_2_CH=CH_2_ as a nucleophile has not been reported yet.

Among oxidants tested in this study, ceric ammonium nitrate (CAN) proved to be an effective oxidant albeit with lower yield (62%) (entry 5, Table 1) compared with DDQ. However, other oxidants including (diacetoxyiodo)benzene (PhI(OAc)_2_), 1,4-benzoquinone (1,4-BQ), TBHP (*tert*-butyl hydroperoxide), and silver acetate (AgOAc) did not promote the oxidative allylation reaction, and only unreacted starting material **4a** was recovered (entries 1–4, Table 1). Solvent screening studies revealed that most organic solvents tested were effective (entries 6–14, Table 1). When EtOAc or THF was used, the desired product could be obtained in excellent yields (90 and 92%, respectively) (entries 6–7, Table 1). Also, highly polar solvents such as acetone, DMF, and MeCN resulted in the desired product (±)–**5a** in high yields (70–86%) (entries 9–11, Table 1). Allyltriphenylstannane (Ph_3_SnCH_2_CH=CH_2_) also proved to be an effective nucleophile (entries 12, Table 1). However, low yield (17%) was obtained when allyltrimethylsilane (Me_3_SiCH_2_CH=CH_2_) (entries 13, Table 1) was used as an allyl nucleophile.

**Table 1:** Optimization of oxidative C(1)-allylation of *N*-Boc 1,2,3,4-THIQ **4a** with an electron-rich allylating reagent.

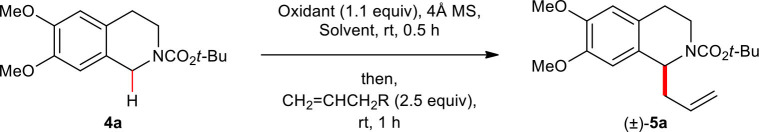					
**Entry**	**Oxidant**	**R**	**Solvent**	**Temp**.	**Yield (%)**
1	PhI(OAc)_2_	(*n*-Bu)_3_Sn	MeCN	rt	0
2	1,4-BQ^a^	(*n*-Bu)_3_Sn	MeCN	rt	0
3	TBHP^b^	(*n*-Bu)_3_Sn	DCM	rt	0
4	AgOAc	(*n*-Bu)_3_Sn	DCM	rt	0
5	CAN^c^	(*n*-Bu)_3_Sn	MeCN	rt	62
6	DDQ	(*n*-Bu)_3_Sn	EtOAc	rt	90
7	DDQ	(*n*-Bu)_3_Sn	THF	rt	92
8	DDQ	(*n*-Bu)_3_Sn	DCM	rt	98 (80)^d^
9	DDQ	(*n*-Bu)_3_Sn	Acetone	rt	86
10	DDQ	(*n*-Bu)_3_Sn	DMF	rt	70
11	DDQ	(*n*-Bu)_3_Sn	MeCN	rt	79
12	DDQ	Ph_3_Sn	DCM	rt	90
13	DDQ	Me_3_Si	DCM	rt	17

All reactions were conducted at 0.1 M concentration with 0.3 mmol of N-Boc 1,2,3,4-THIQ **4a** (1.0 equiv), 0.33 mmol of oxidant (1.1 equiv) in the presence of 120 mg of 4Å molecular sieves (MS) at ambient temperature under argon atmosphere. After 30 min, 0.75 mmol of allyl nucleophiles (2.5 equiv) were added to the reaction mixture, and the reaction mixture was stirred for 1 h. Yield was based on isolated product after purification by chromatography. ^*a*^1,4-benzoquinone; ^*b*^tert-butyl hydroperoxide; ^*c*^ceric ammonium nitrate; ^*d*^yield in the absence of 4Å molecular sieves (MS).

### Scope and Limitations of the Reaction

**Scheme 3:**
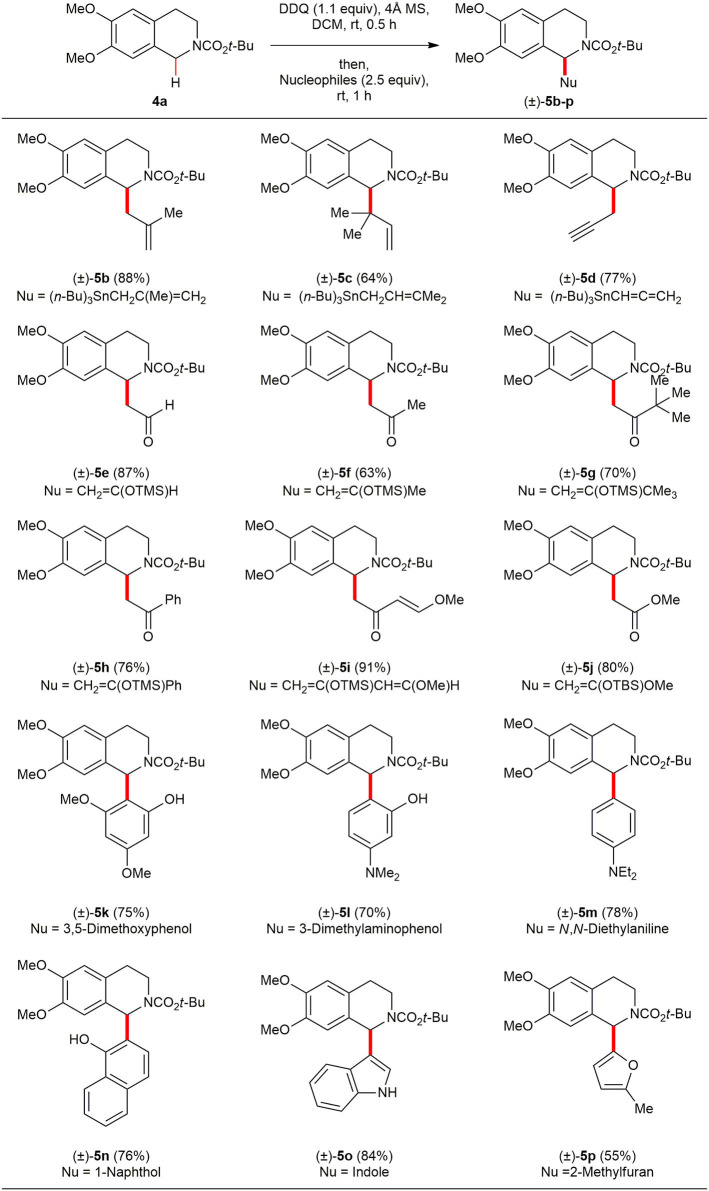
Reaction scope of *N*-Boc 1,2,3,4-THIQ **4a** and electron-rich nucleophiles. All reactions were conducted at 0.1 M concentration with 0.3 mmol of *N*-Boc 1,2,3,4-THIQ **4a** (1.0 equiv), 0.33 mmol of DDQ (1.1 equiv) in the presence of 120 mg of 4Å MS at ambient temperature under argon atmosphere. After 30 min, 0.75 mmol of nucleophiles were added to the reaction mixture, and the reaction mixture was stirred for 1 h. Yield was based on isolated product after purification by chromatography.

With optimized reaction conditions in hand, the scope of the oxidative direct C(sp^3^)–H functionalization was investigated with a diverse range of electron-rich nucleophiles (Scheme 3). The reactions of methallyltributylstannane [(*n*-Bu)_3_SnCH(Me)CH=CH_2_] and dimethyltributylstannane [(*n*-Bu)_3_SnCH_2_CH=CMe_2_] provided the corresponding C(1)-allylated products (±)–**5b** and (±)–**5c** in 88% and 64% yields, respectively. Also, allenyltributylstannane [(*n*-Bu)_3_SnC=C=CH_2_] provided the desired C(1)-propargylated product (±)–**5d** in 77% yield. Although a variety of ketones have been widely employed as pro-nucleophiles in cross dehydrogenative coupling (CDC) reactions of *N*-aryl 1,2,3,4-THIQs, the use of electron-rich silyl enol ethers (Scott et al., 2014) or silyl ketene acetals have rarely been reported. A diverse range of silyl enol ethers and a silyl ketene acetal have been tested in order to expand the scope and utility of this oxidative DDQ-promoted direct C(sp^3^)–H functionalization of *N*-acyl 1,2,3,4-THIQs. All of the silyl enol ethers tested so far worked rather well with **4a** to provide Mannich products (±)–**5e**-**j** in isolated yield ranging from 63 to 91%. The reaction could also be readily expanded to oxidative Friedel–Crafts-type reaction. Under the optimal reaction condition, **4a** with 3,5-dimethoxyphenol, 3-dimethylaminophenol and 1-naphthol afforded Friedel–Crafts products (±)-**5k** (75%), (±)–**5l** (70%), and (±)–**5n** (76%) in good yields. In these examples, **4a** was coupled to phenols selectively at the *ortho*-position, while the phenolic-OH is unaffected. Furthermore, we found that *N, N*-diethylaniline, indole, and 2-methyl furan are good nucleophiles for this oxidative DDQ-promoted direct C(sp^3^)–H functionalization to afford the corresponding Friedel-Crafts products (±)–**5m** (78%), (±)–**5o** (84%), and (±)–**5p** (55%). To the best of our knowledge, this is the first report using electron-rich nucleophiles in oxidative direct C(sp^3^)–H functionalization of a *N*-acyl 1,2,3,4-THIQ.

**Scheme 4:**
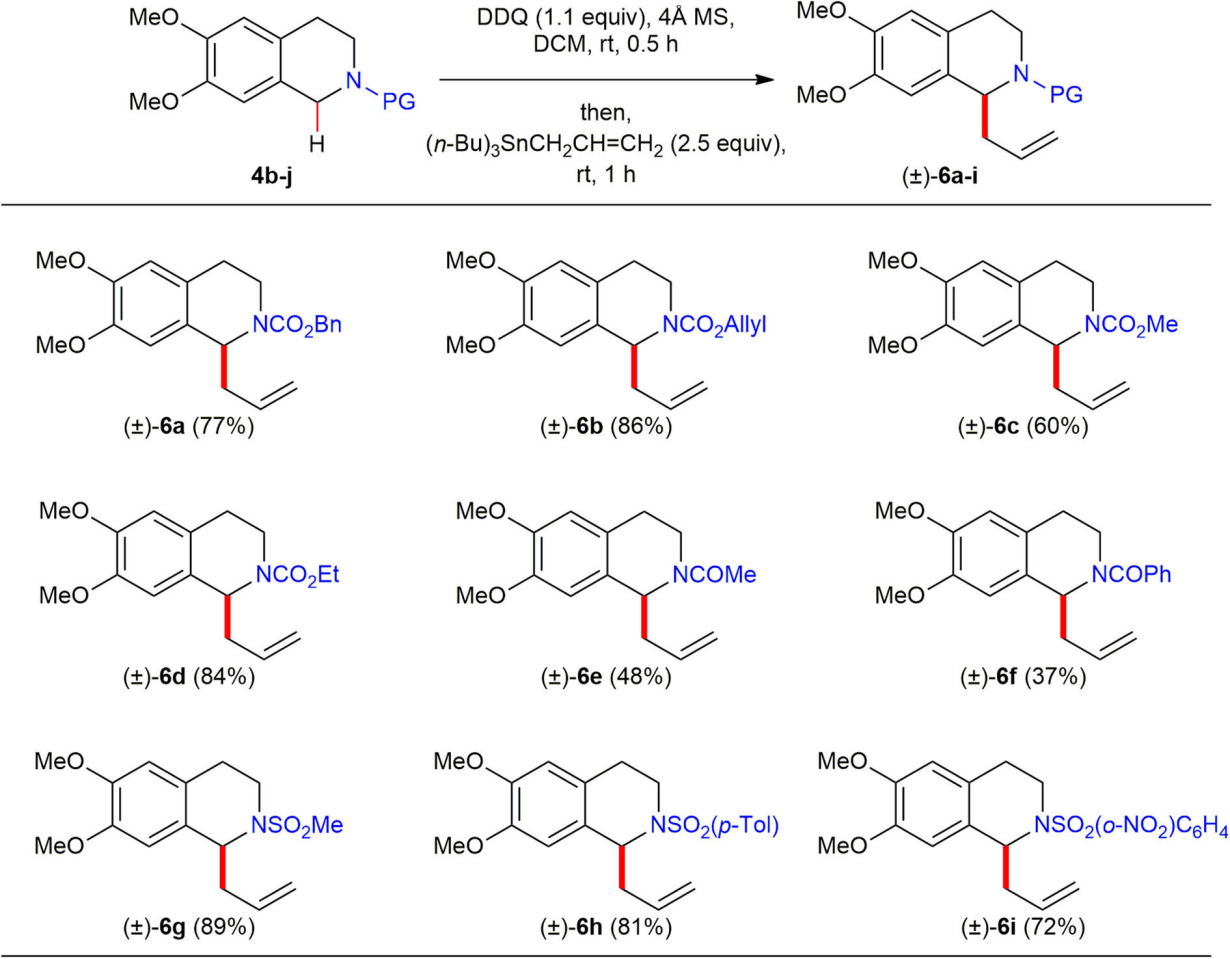
Reaction scope of *N*-acyl/sulfonyl 1,2,3,4-THIQs **4b**–**j** and allyltributylstannane. All reactions were conducted at 0.1 M concentration with 0.3 mmol of *N*-acyl/sulfonyl 1,2,3,4-THIQs **4b**–**j** (1.0 equiv), 0.33 mmol of DDQ (1.1 equiv) in the presence of 120 mg of 4Å MS at ambient temperature under argon atmosphere. After 30 min, 0.75 mmol of allyltributylstannane (2.5 equiv) was added to the reaction mixture, and the reaction mixture was stirred for 1 h. Yield was based on isolated product after purification by chromatography.

To make this oxidative direct C(sp^3^)–H functionalization synthetically useful, we explored the use of a broad range of *N*-acyl/sulfonyl THIQs since installation and liberation of their amine protecting groups are easy and operationally convenient (Scheme 4). The C(1)-allylation reaction of benzyl- (**4b**), allyl- (**4c**), methyl- (**4d**), and ethyl (**4e**) carbamates under DDQ-promoted oxidative reaction conditions all provided the corresponding C(1)-allylated products (±)–**6a**–**d** in good to excellent yields (60–84%). Furthermore, reactions of *N*-sulfonamides such as *N*-Ts (**4h**), *N*-Ms (**4i**), *N*-Ns (**4j**) generated the corresponding C(1)-allylated products (±)–**6g**–**i** in high yields (72–89%). However, amides such as acetamide (**4f**) and benzamide (**4g**) proved to be ineffective substrates to afford the corresponding C(1)-allylated products (±)–**6e** and (±)–**6f** in low yield (48 and 37%, respectively) under the optimized reaction conditions.

**Scheme 5:**
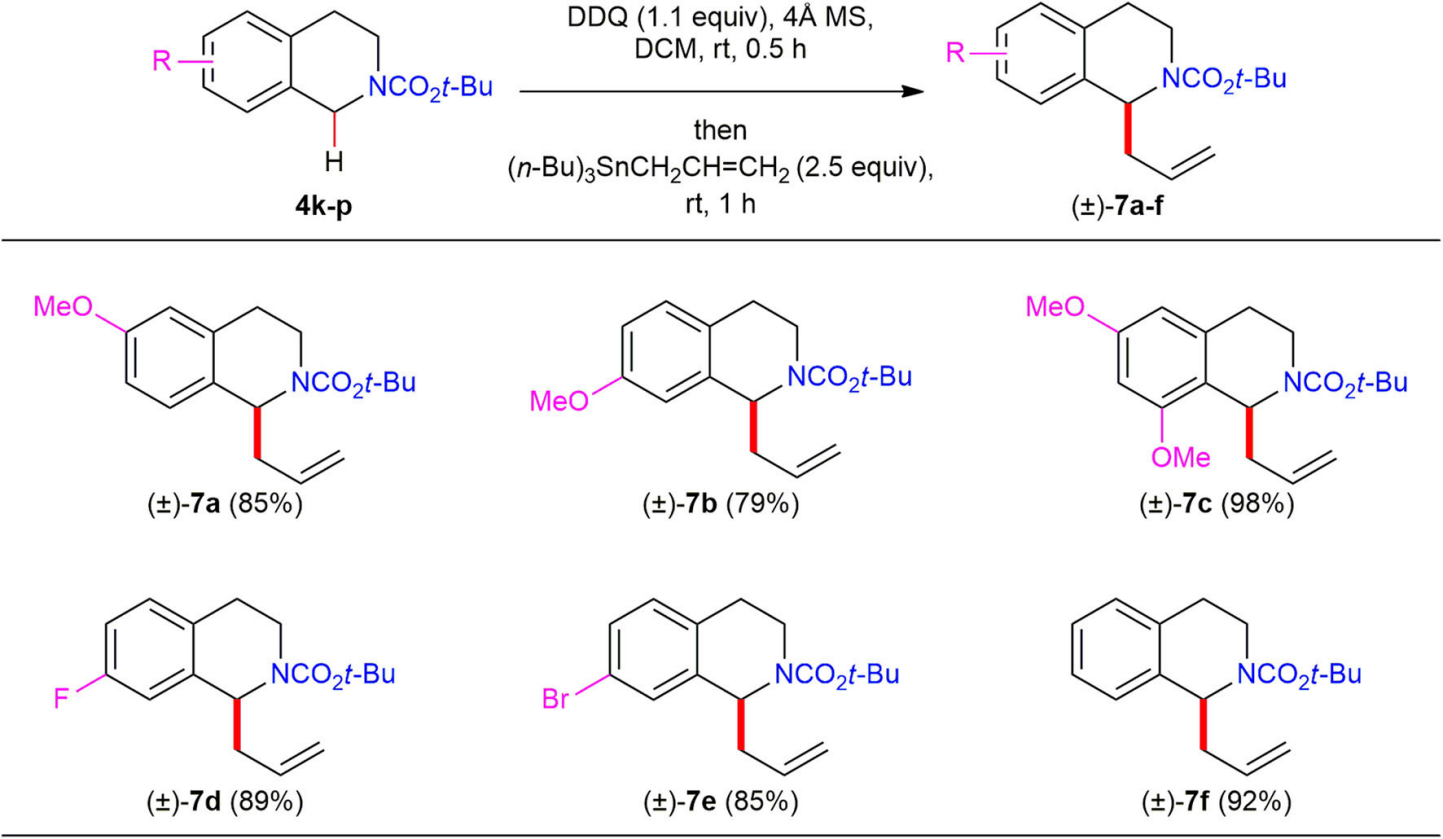
Reaction scope of electronically diverse *N*-Boc 1,2,3,4-THIQs **4k**–**p** and allyltributylstannane. All reactions were conducted at 0.1 M concentration with 0.3 mmol of *N*-Boc 1,2,3,4-THIQs **4k**–**p** (1.0 equiv), 0.33 mmol of DDQ (1.1 equiv) in the presence of 120 mg of 4Å MS at ambient temperature under argon atmosphere. After 30 min, 0.75 mmol of allyltributylstannane (2.5 equiv) was added to the reaction mixture, and the reaction mixture was stirred for 1 h. Yield was based on isolated product after purification by chromatography.

We further investigated the substrate scope with respect to electronically diverse *N*-Boc 1,2,3,4-THIQs (Scheme 5). As expected, direct C(1)-allylation of *N*-Boc 1,2,3,4-THIQs **4k**–**m** bearing electron-donating substituents on the phenyl moiety led to the corresponding C(1)-allylated products (±)–**7a**–**7c** in high yields (79–98%). Notably, *N*-Boc 1,2,3,4-THIQs bearing electron-withdrawing substituents such as fluorine (**4n**) and bromine (**4o**) on the phenyl moiety were also tolerated to furnish the corresponding C(1)-allylated products (±)–**7d** and (±)–**7e** in high yield (89 and 85%, respectively), which are useful for further diversifications. Also, *N*-Boc 1,2,3,4-THIQ **4p** with no substituents on the phenyl moiety was found to be effective to afford the desired C(1)-allylated product (±)–**7r** in 92% yield.

With the desired *N*-acyl/sulfonyl C(1)-substituted 1,2,3,4-THIQs in hand, a variety of means for liberation of the C(1)-allylated 1,2,3,4-THIQs were investigated (Table 2). The *tert*-butoxycarbonyl (Boc) group of (±)–**5a** was cleanly removed under acidic (CF_3_CO_2_H) conditions to give free amine (±)–**8** in high yield (88%). Alkaline hydrolysis of the methoxycarbonyl group in (±)–**6c** with KOH by heating at reflux in ethylene glycol furnished free amine (±)–**8** in good yield (70%). Also, removal of the 2-nitrobenzenesulfonyl (Ns) group of (±)–**6h** proceeded smoothly by employing the condition (PhSH and K_2_CO_3_) reported by Fukuyama and co-worker (Fukuyama et al., 1995) to afford free amine (±)–**8** in high yield (88%).

**Table 2:** Liberation of C(1)-allylated 1,2,3,4-THIQ (±)–**8** from (±)–**5a**, **6c**, and **6h**.

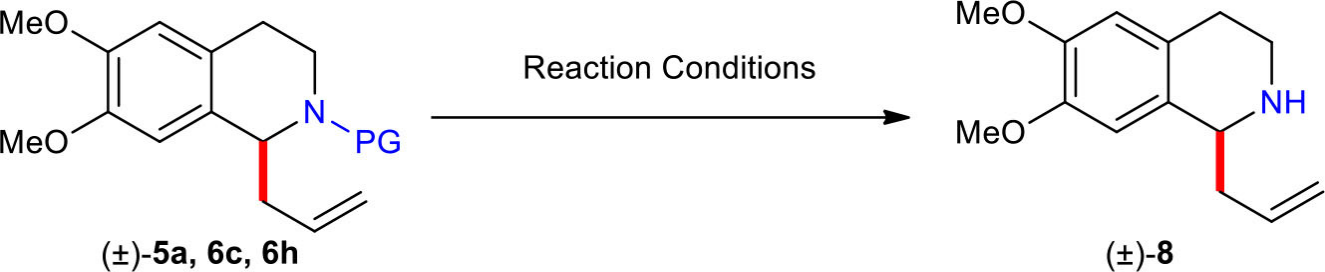					
**Entry**	**Reactant**	**PG**	**Reaction conditions**	**Yield (%)**
1	**5a**	CO_2_*t*-Bu (Boc)	TFA, DCM, rt, 2 h	88
2	**6c**	CO_2_Me	KOH, H_2_O, ethylene glycol, reflux, 12 h	70
3	**6h**	SO_2_(*o*-NO_2_)C_6_H_4_ (Ns)	PhSH, K_2_CO_3_, DMF, rt, 18 h	88

### Proposed Reaction Mechanism

**Scheme 6:**
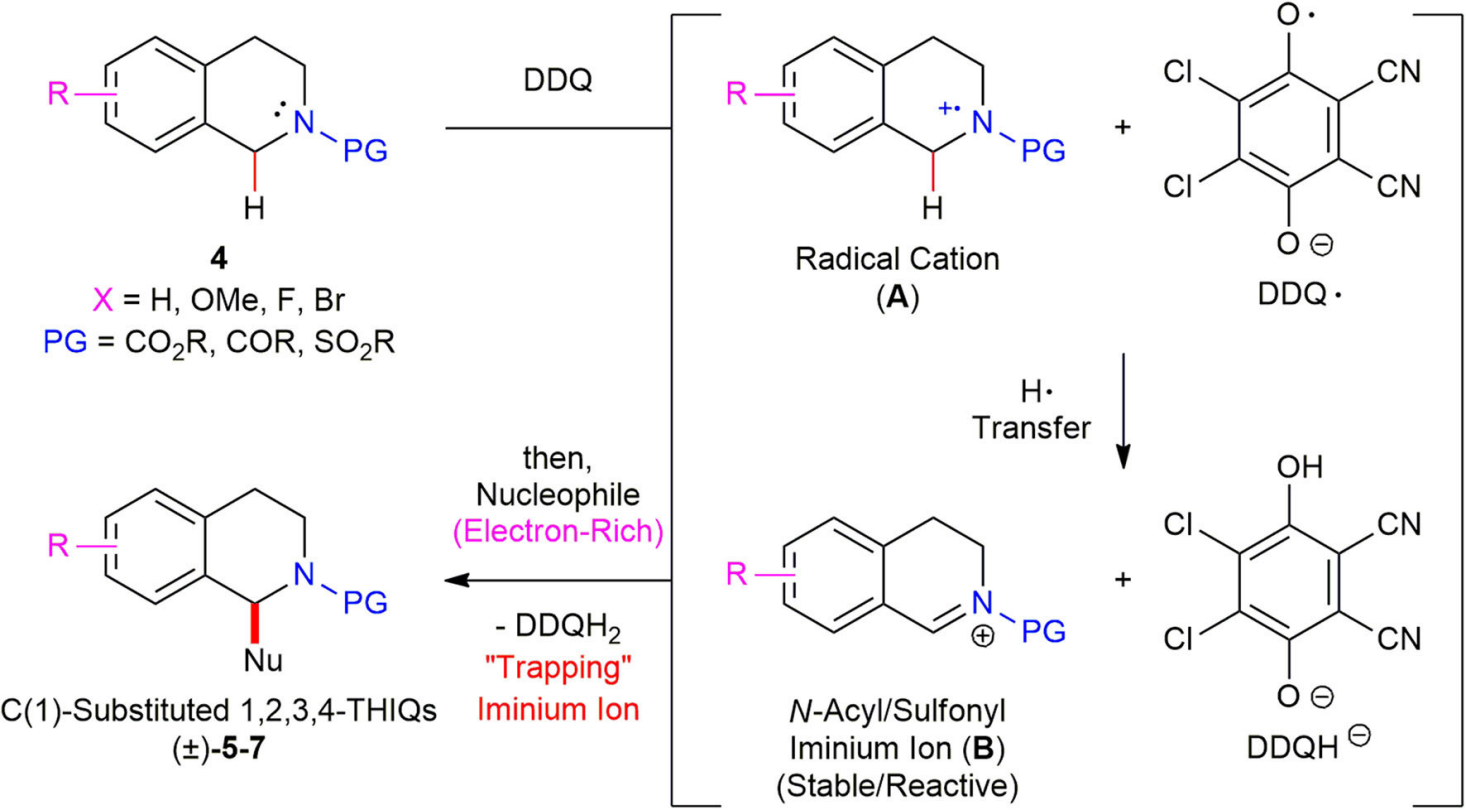
Proposed reaction mechanism for DDQ-promoted C(1)-allylation of *N*-acyl/sulfonyl 1,2,3,4-THIQs.

In order to gain some mechanistic insight into the reaction mechanism, the radical inhibition experiments were conducted. When 2,6-di-*tert*-butyl-4-methylphenol (BHT) (1.1 equiv) was added to the reaction mixture of *N*-Boc 1,2,3,4-tetrahydroisoquinoline **4a** (1.0 equiv) and DDQ (1.1 equiv), the yield of the desired product (±)–**5a** was dramatically decreased from 98 to 20% and 79% of the starting material **4a** was recovered. This result suggests that a radical cation species might be involved in the reaction. On the basis of the radical inhibition experiments and literature precedents (Muramatsu et al., 2013; Chen et al., 2015), a plausible reaction mechanism for the DDQ-promoted oxidative direct C(sp^3^)–H functionalization of *N*-acyl/sulfonyl 1,2,3,4-THIQ **4** was proposed (Scheme 6). *N*-Acyl/sulfonyl 1,2,3,4-THIQ **4** undergoes a single electron transfer from *N*-acyl/sulfonyl 1,2,3,4-THIQ **4** to DDQ to generate a radical cation (**A**). The DDQ radical oxygen then abstracts a H-atom from **A**, leading to a stable and reactive *N*-acyl/sulfonyl iminium ion (**B**). Finally, the trapping the iminium ion (**B**) with a diverse range of electron-rich nucleophiles afforded the desired *N*-acyl/sulfonyl C(1)-substituted THIQs (±)–**5**–**7**.

### A Concise and Efficient 3-Step Total Synthesis of (±)-Benzo[*a*]quinolizidine

**Scheme 7:**
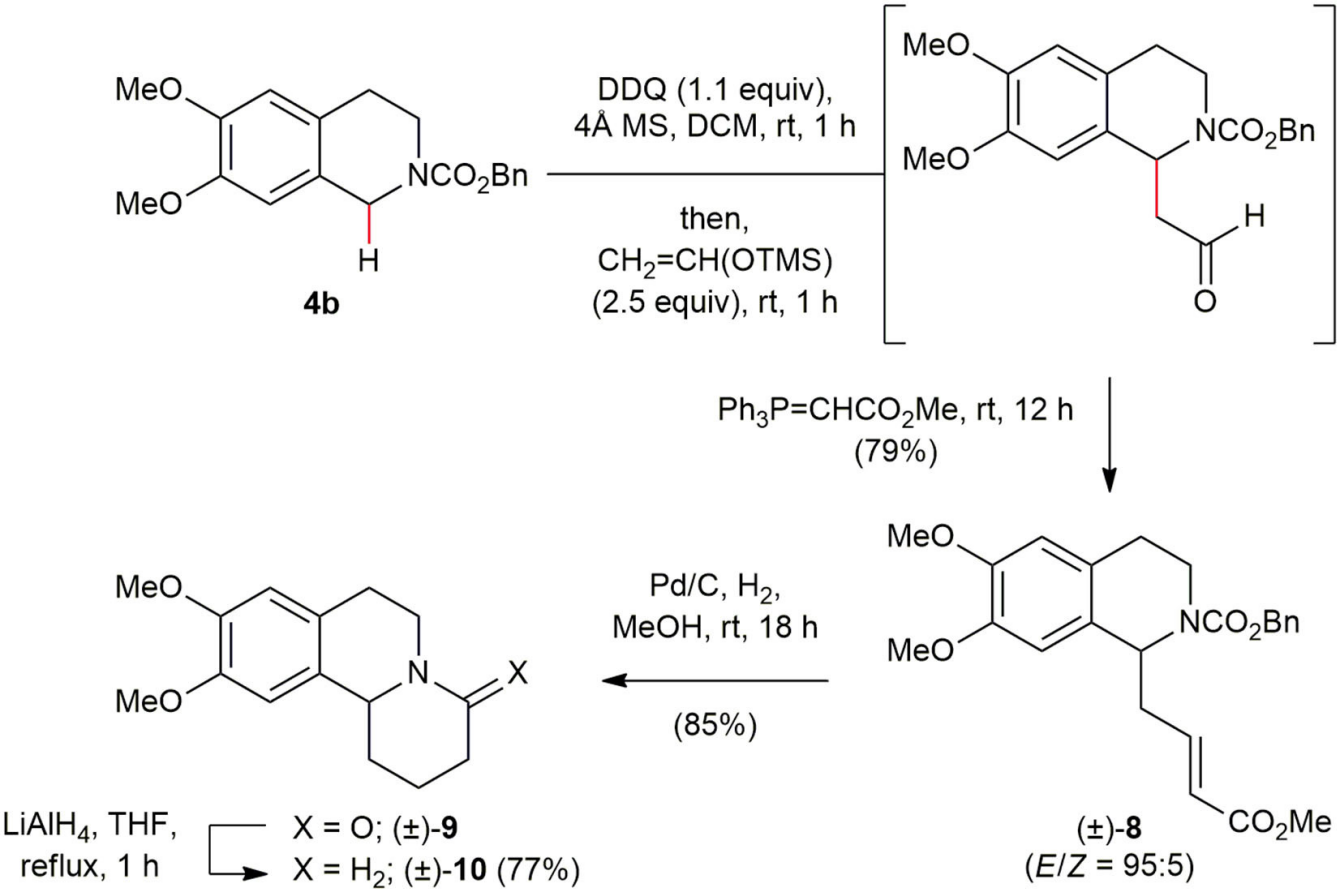
A concise and efficient 3-step total synthesis of (±)-benzo[*a*]quinolizidine (**11**).

We next turned on our attention to a short and efficient total synthesis of (±)-benzo[*a*]quinolizidine (**10**) to prove the synthetic utility of this method (Scheme 7). The oxidative direct C(sp^3^)–H functionalization of the readily available *N*-Cbz 1,2,3,4-THIQ **4b** (Dunetz et al., 2005; Kim et al., 2018) with CH_2_=C(OTMS)H afforded aldehyde, which underwent Wittig olefination with a two carbon stabilized ylide Ph_3_P=CHCO_2_Me to furnish α,β-unsaturated ester (±)–**8** in 79% yield in a one-pot fashion, exhibiting high stereoselectivity (*E*/*Z* = 95:5), that is none the less to be rendered in consequential at this stage because the planned hydrogenation/deprotection/ring-closure reaction sequence was envisaged to provide a single product regardless of the olefin geometry. The hydrogenation of the olefin moiety, simultaneous deprotection of the Cbz group on the nitrogen atom of the THIQ framework and ring closure was achieved smoothly by hydrogenation (1 atm) over 10% Pd/C in EtOAc to provide the desired lactam (±)–**9** in 85%. Reduction of lactam (±)–**9** with LiAlH_4_ in THF according to Reddy and co-workers (Reddy et al., 2013) afforded (±)-benzo[*a*]quinolizidine (**10**) in 77%, whose spectral data were in good agreement with those reported in the literature (Williams et al., 2005; Szawkalo et al., 2007; Reddy et al., 2013; Talk et al., 2016).

## Conclusions

In conclusion, *N*-acyl/sulfonyl 1,2,3,4-THIQ iminium complexes *in situ* generated by DDQ were found to be very effective and compatible with a wide range of electron-rich nucleophiles. New and useful nucleophiles such as silyl enol ethers and silyl ketene acetals are employed to afford Mannich-type products and use of phenols, heteroaromatics furnished Friedel–Crafts-type products. Further studies are ongoing to expand the synthetic utility of this products to natural product or synthetically useful compounds.

## Materials and Methods

### General Information

#### General Methods

Except as otherwise indicated, reactions were carried out under argon atmosphere in flame- or oven-dried glassware. In aqueous work-up, all organic solutions were dried over sodium sulfate (Na_2_SO_4_) or magnesium sulfate (MgSO_4_), and filtered prior to rotary evaporation at water aspirator pressure. Reactions were monitored by thin layer chromatography (TLC) with 0.25-mm E. Merck pre-coated silica gel plates (Kieselgel 60F_254_, Merck). Spots were detected by viewing under a UV light, colorizing with charring after dipping in *p*-anisaldehyde solution with acetic acid and sulfuric acid and ethanol, or ceric ammonium molybdate solution with sulfuric acid and ethanol. Silica gel for flash chromatography (particle size 0.040–0.063 mm) was supplied by E. Merck. Yields refer to chromatographically and spectroscopically pure compounds unless otherwise noted.

#### Materials

All commercial reagents and solvents were purchased from Sigma Aldrich Co. or Tokyo Chemical Industry (TCI) and used as received with the following exceptions. All solvents were freshly purified and dried by standard techniques just before use. Tetrahydrofuran (THF) was distilled from sodium/benzophenone. Dichloromethane (CH_2_Cl_2_), acetonitrile (MeCN), *N, N*-dimethylformamide (Me_2_NC(=O)H), benzene (C_6_H_6_) and toluene (C_7_H_8_) were distilled from calcium hydride (CaH_2_). Methanol (MeOH) was distilled from magnesium sulfate (MgSO_4_).

#### Instrumentation

^1^H and ^13^C spectra were recorded on Varian Mercury-400BB (400 MHz). Chemical shifts are reported as δ value relative to internal chloroform (δ 7.26 for ^1^H and δ 77.0 for ^13^C). Data are represented as follows: chemical shift, multiplicity (s = singlet, d = doublet, t = triplet, q = quartet, m = multiplet, br = broad), coupling constant in Hz, and integration. High resolution mass spectra (HRMS) were recorded on JEOL JMS-700 (FAB or EI) mass spectrometer. High resolution values are calculated to four decimal places from the molecular formula, all found values being within a tolerance of 5 ppm.

### Synthesis of *N*-Protected 1-Substituted-1,2,3,4-Tetrahydroisoquinolines

To a stirred solution of *N*-protected 1,2,3,4-tetrahydroisoquinoline (0.30 mmol) in DCM (3.0 mL, 0.1M) was added 4Å molecular sieves (120 mg) at room temperature. After the reaction mixture was stirred for 15 min at room temperature, 2,3-dichloro-5,6-dicyano-1,4-benzoquinone (DDQ) (0.45 mmol, 1.1 equiv) was added portionwise and the reaction mixture was stirred at room temperature for 30 min under argon atmosphere. Nucleophile (0.75 mmol, 2.5 equiv) was added dropwise or portionwise at room temperature. The reaction mixture was stirred at room temperature for 1 h under argon atmosphere, then quenched with saturated NaHCO_3_ solution (10 mL) and the layers were separated. The aqueous layer was extracted with DCM (2 × 25 mL), and the combined organic layer was washed with brine (5 mL), dried over Na_2_SO_4_, filtered, and concentrated *in vacuo*. Purification of the residue by flash column chromatography on silica gel, using hexanes/EtOAc as eluent, provided the corresponding *N*-protected 1-substituted-1,2,3,4-tetrahydroisoquinoline.

(±)-*tert*-Butyl 1-allyl-6,7-dimethoxy-3,4-dihydroisoquinoline-2(1*H*)-carboxylate (**5a**). Yield 98% as a colorless oil. ^1^H NMR (400 MHz, CDCl_3_, a 1.5:1 mixture of amide rotamers at room temperature) δ 6.60 (s, 2H), 5.80–5.90 (m, 1H), 5.16 (brs, 0.4H), 5.01–5.07 (m, 2.6H), 4.20–4.23 (m, 0.6H), 3.97–4.02 (m, 0.4H), 3.86 (s, 1.2H), 3.84 (1.8H), 3.23–3.28 (m, 0.4H), 3.12–3.18 (m, 0.6H), 2.82–2.90 (m, 1H), 2.64 (t, *J* = 3.6 Hz, 0.6H), 2.60 (t, *J* = 3.6 Hz, 0.4H), 2.52 (t, *J* = 7.2 Hz, 2H), 1.47 (s, 9H); ^13^C NMR (100 MHz, CDCl_3_, a rotameric mixture, resonances for minor rotamer are enclosed in parenthesis) δ 153.9, (147.0), 146.7, 134.6, (128.7), 128.4, 125.8, (125.5), 116.6, (116.2), 111.0, (110.9), (109.7), 109.4, 79.1, (78.8), 55.5, (55.4), 53.7, (52.8), 41.1, (40.8), (38.0), 36.3, 28.1, (27.9), 27.8; IR (Film) 2975, 1691, 1519, 1422, 1259, 1165 (cm^−1^); HRMS (EI-magnetic sector) *m/z*: {M}^+^ Calcd for C_19_H_27_NO_4_: 333.1940; Found 333.1936.

(±)-*tert*-Butyl 6,7-dimethoxy-1-(2-methylallyl)-3,4-dihydroisoquinoline-2(1*H*)-carboxylate (**5b**). Yield 88% as a colorless oil. ^1^H NMR (400 MHz, CDCl_3_, a 1.5:1 mixture of amide rotamers at room temperature) δ 6.56–6.60 (m, 2H), 5.27 (dd, *J* = 8.8, 5.6 Hz, 0.4H), 5.07 (dd, *J* = 8.8, 5.2 Hz, 0.6H), 4.82 (s, 0.6H), 4.78 (s, 0.4H), 4.68 (s, 1H), 4.24 (dd, *J* = 13.4, 3.8 Hz, 0.6H), 4.00 (dd, *J* = 13.6, 3.2 Hz), 3.86 (s, 1.2H), 3.85 (s, 1.8H), 3.13–3.29 (m, 1H), 2.79–2.93 (m, 1H), 2.60–2.64 (m, 1H), 2.49–2.55 (m, 1H), 2.28–2.39 (m, 1H), 1.89 (s, 3H), 1.47 (s, 5.4H), 1.45 (s, 3.6H); ^13^C NMR (100 MHz, CDCl_3_, a rotameric mixture, resonances for minor rotamer are enclosed in parenthesis) δ 154.5, 147.6, (147.4), 147.0, (142.7), 141.8, (129.9), 129.3, 126.3, (125.9), 114.0, 113.2, 111.5, (111.3), (110.2), 110.1, 79.8, (79.3), (56.1), 55.9, 52.9, (52.1), 45.3, (45.1), (38.0), 36.5, 28.5, (28.3), 28.2, 22.9, (22.6); IR (Film) 2971, 1684, 1516, 1419, 1240, 1161 (cm^−1^); HRMS (FAB-magnetic sector) *m/z*: {M+H}^+^ Calcd for C_20_H_30_NO_4_: 348.2175; Found 348.2183.

(±)-*tert*-Butyl 6,7-dimethoxy-1-(2-methylbut-3-en-2-yl)-3,4-dihydroisoquinoline-2(1*H*)-carboxylate (**5c**). Yield 64% as a colorless oil. ^1^H NMR (400 MHz, CDCl_3_, a 1:1 mixture of amide rotamers at room temperature) δ 6.74 (s, 0.5H), 6.72 (s, 0.5H), 6.60 (s, 0.5H), 6.58 (s, 0.5H), 5.87 (dd, *J* = 17.6, 11.2 Hz, 0.5H), 5.83 (dd, *J* = 16.4, 10.8 Hz, 0.5H), 5.08 (s, 0.5H), 4.97 (s, 0.5H), 4.91–4.95 (m, 2H), 4.18 (ddd, *J* = 12.8, 7.6, 7.6 Hz, 0.5H), 3.85–3.92 (m, 0.5H), 3.86 (s, 1.5H), 3.85 (s, 1.5H), 3.84 (s, 1.5H), 3.82 (s, 1.5H), 3.52 (ddd, *J* = 14.8, 7.2, 7.2 Hz, 0.5H), 3.39 (ddd, *J* = 15.6, 9.6, 6.0 Hz, 0.5H), 2.69–2.88 (m, 2H), 1.49 (s, 4.5H), 1.46 (s, 4.5H), 1.14 (s, 3H), 1.11 (s, 1.5H), 1.08 (s, 1.5H); ^13^C NMR (100 MHz, CDCl_3_, a rotameric mixture, resonances for minor rotamer are enclosed in parenthesis) δ 155.5, (154.9), (147.7), 147.5, (147.2), 147.0, 146.2, 127.2, (127.0), 126.8, 112.0, 111.5, (111.34), 111.25, (111.1), 79.9, (79.4), (61.7), 61.0, 56.1, (56.0), 55.9, 43.8, 39.6, (38.0), (28.7), 28.6, (27.7), 27.6, 27.5, (27.3), (24.2), 24.0; IR (Film) 2970, 1684, 1517, 1364, 1249, 1160 (cm^−1^); HRMS (FAB-magnetic sector) *m/z*: {M+H}^+^ Calcd for C_21_H_32_NO_4_: 362.2331; Found 362.2331.

(±)-*tert*-Butyl 6,7-dimethoxy-1-(prop-2-yn-1-yl)-3,4-dihydroisoquinoline-2(1*H*)-carboxylate (**5d**). Yield 77% as a colorless oil. ^1^H NMR (400 MHz, CDCl_3_, a 1.4:1 mixture of amide rotamers at room temperature) δ 6.76 (s, 1H), 6.61 (s, 1H), 5.25 (t, *J* = 6.4 Hz, 0.42H), 5.13 (t, *J* = 6.4 Hz, 0.58H), 4.14–4.23 (m, 0.58H), 3.90–3.97 (m, 0.42H), 3.87 (s, 2.52H), 3.86 (s, 3.48H), 3.40–3.46 (m, 0.42H), 3.23–3.30 (m, 0.58H), 2.65–2.90 (m, 4H), 2.01 (s, 0.58H), 1.98 (s, 0.42H), 1.51 (s, 5.22H), 1.49 (s, 3.78H); ^13^C NMR (100 MHz, CDCl_3_, a rotameric mixture, resonances for minor rotamer are enclosed in parenthesis) δ (154.4), 154.2, 147.7, 147.0, (127.6), 127.4, 126.5, (126.3), 111.2, (111.0), (110.2), 109.9, 81.4, 80.0, (79.7), 70.7, (70.6), 55.9, 55.8, 53.1, (52.4), (39.1), 37.3, 28.5, (28.4), 28.2, 26.6, (26.2); IR (Film) 3287, 2974, 2118, 1690, 1519, 1259 (cm^−1^); HRMS (FAB-magnetic sector) *m/z*: {M}^+^ Calcd for C_19_H_25_NO_4_: 331.1784; Found 331.1779.

(±)-*tert*-Butyl 6,7-dimethoxy-1-(2-oxoethyl)-3,4-dihydroisoquinoline-2(1*H*)-carboxylate (**5e**). Yield 87% as a white foam. ^1^H NMR (400 MHz, CDCl_3_, a 1:1 mixture of amide rotamers at room temperature) δ 9.84 (t, *J* = 3.6 Hz, 1H), 6.62 (s, 3H), 6.60 (s, 3H), 5.61–5.71 (m, 0.5H), 5.46–5.54 (m, 0.5H), 4.15–4.30 (m, 1H), 3.94–4.02 (m, 0.5H), 3.85 (s, 3H), 3.10–3.40 (m, 1H), 2.76–2.96 (m, 3H), 2.68 (t, *J* = 3.6 Hz, 0.5H), 2.64 (t, *J* = 3.6 Hz, 0.5H), 1.47 (s, 9H); ^13^C NMR (100 MHz, CDCl_3_, a rotameric mixture, resonances for minor rotamer are enclosed in parenthesis) δ 199.5, 154.0, (153.2), 147.3, 147.1, 127.3, 125.9, (125.8), 111.0, 109.0, 80.1, (79.6), 55.5, 55.4, 50.8, (49.5), 48.9, 38.3, (36.9), 28.0, (27.7), 27.6; IR (Film): 2975, 1722, 1689, 1519, 1419, 1258, 1163 (cm^−1^); HRMS (EI-magnetic sector) *m/z*: {M}^+^ Calcd for C_18_H_25_NO_5_: 335.1733; Found 335.1725.

(±)-*tert*-Butyl 6,7-dimethoxy-1-(2-oxopropyl)-3,4-dihydroisoquinoline-2(1*H*)-carboxylate (**5f**). Yield 63% as a colorless oil. ^1^H NMR (400 MHz, CDCl_3_, a 1.2:1 mixture of amide rotamers at room temperature) δ 6.68 (s, 0.45H), 6.65 (s, 0.55H), 5.61 (s, 0.45H), 5.48 (s, 0.55H), 4.14–4.18 (m, 0.45H), 3.88–3.94 (m, 0.55H), 3.85 (s, 3H), 3.29–3.34 (m, 0.45H), 3.18–3.23 (m, 0.55H), 2.75–2.94 (m, 3H), 2.67 (t, *J* = 4.0 Hz, 0.55H), 2.63 (t, *J* = 4.0 Hz, 0.45H), 2.25 (s, 1.35H), 2.19 (s, 1.65H), 1.47 (s, 9H); ^13^C NMR (100 MHz, CDCl_3_, a rotameric mixture, resonances for minor rotamer are enclosed in parenthesis) δ (206.3), 206.0, (154.4), 153.9, 147.5, 147.3, 128.5, 126.0, (125.9), 111.2, (109.7), 109.3, 80.3, (79.8), 55.9, 55.8, 51.2, 51.1, (50.5), (38.8), 37.5, 31.2, (30.2), 28.4, (28.1), 27.9; IR (Film): 2976, 1689, 1519, 1418, 1222, 1164 (cm^−1^); HRMS (EI-magnetic sector) *m/z*: {M}^+^ Calcd for C_19_H_27_NO_5_: 349.1889; Found 349.1893.

(±)-*tert*-Butyl 1-(3,3-dimethyl-2-oxobutyl)-6,7-dimethoxy-3,4-dihydroisoquinoline-2(1*H*)-carboxylate (**5g**). Yield 63% as a white foam. ^1^H NMR (400 MHz, CDCl_3_, a 1.5:1 mixture of amide rotamers at room temperature) δ 6.68 (s, 0.4H), 6.64 (s, 0.6H), 6.59 (s, 1H), 5.62 (t, *J* = 6.4 Hz, 1H), 4.08–4.12 (m, 0.6H), 3.84 (s, 3H), 3.81 (s, 3H), 3.81–3.84 (m, 0.4H), 3.34–3.43 (m, 0.4H), 3.18–3.28 (m, 0.6H), 2.65–3.03 (m, 4H), 1.47 (s, 9H), 1.07 (s, 9H); ^13^C NMR (100 MHz, CDCl_3_, a rotameric mixture, resonances for minor rotamer are enclosed in parenthesis) δ 212.3, 154.1, 147.4, 147.2, (129.5), 129.3, 126.0, 111.1, (110.0), 109.5, 80.0, (79.5), 55.8, 50.8, (50.5), 44.9, 44.3, (44.2), (39.6), 38.1, 28.4, 28.1, 26.0; IR (Neat): 2974, 1691, 1517, 1364, 1257, 1220 (cm^−1^); HRMS (EI-magnetic sector) *m/z*: {M}^+^ Calcd for C_22_H_33_NO_5_: 391.2359; Found 391.2366.

(±)-*tert*-Butyl 6,7-dimethoxy-1-(2-oxo-2-phenylethyl)-3,4-dihydroisoquinoline-2(1*H*)-carboxylate (**5h**). Yield 76% as a white foam. ^1^H NMR (400 MHz, CDCl_3_, a 1:1 mixture of amide rotamers at room temperature) δ 7.95 (d, *J* = 7.2 Hz, 2H), 7.52–7.60 (m, 1H), 7.47 (t, *J* = 7.2 Hz, 2H), 6.69 (s, 0.75H), 6.64 (s, 0.25H), 6.61 (s, 1H), 5.69–5.74 (m, 0.25H), 5.66 (dd, *J* = 6.0, 5.6 Hz, 0.75H), 4.19–4.22 (m, 0.5H), 3.85 (s, 3H), 3.80 (s, 2.25H), 3.75 (s, 0.75H), 3.45–3.50 (m, 1.5H), 3.22–3.34 (m, 2H), 2.69–2.93 (m, 2H), 1.41 (s, 2.25H), 1.30 (s, 6.75H); ^13^C NMR (100 MHz, CDCl_3_, a rotameric mixture, resonances for minor rotamer are enclosed in parenthesis) δ 197.2, (154.0), 153.6, 147.5, 147.1, (146.9), (136.8), 136.6, 132.8, (132.5), 128.5, 128.3, 128.2, 127.9, 126.0, 111.2, (111.0), (109.9), 109.5, 79.8, (79.14), 55.6, 51.8, (51.2), 46.1, (45.8), (39.3), 37.4, 28.2, 27.9; IR (Film): 2976, 1690, 1518, 1418, 1256, 1164 (cm^−1^); HRMS (EI-magnetic sector) *m/z*: {M}^+^ Calcd for C_24_H_29_NO_5_: 411.2046; Found 411.2052.

(±)-(*E*)-*tert*-Butyl 6,7-dimethoxy-1-(4-methoxy-2-oxobut-3-en-1-yl)-3,4-dihydroisoquinoline-2(1*H*)-carboxylate (**5i**). Yield 91% as a colorless oil. ^1^H NMR (400 MHz, CDCl_3_, a 2:1 mixture of amide rotamers at room temperature) δ 7.65 (d, *J* = 12.4 Hz, 0.33H), 7.57 (d, *J* = 12.8 Hz, 0.67H), 6.67 (s, 1H), 6.59 (s, 1H), 5.70 (d, *J* = 12.4 Hz, 0.33H), 5.59 (d, *J* = 12.8 Hz, 0.67H), 5.50–5.53 (m, 1H), 4.19–4.22 (m, 0.33H), 3.85–3.90 (m, 0.67H), 3.84 (s, 3H), 3.83 (s, 3H), 3.70 (s, 3H), 3.30–3.40 (m, 0.33H), 3.17–3.22 (m, 0.67H), 2.97 (d, *J* = 7.2 Hz, 0.33H), 2.93 (d, *J* = 6.8 Hz, 0.67H), 2.72–2.88 (m, 2H), 2.68 (t, *J* = 3.6 Hz, 0.67H), 2.64 (t, *J* = 3.6 Hz, 0.67H), 1.45 (s, 9H); ^13^C NMR (100 MHz, CDCl_3_, a rotameric mixture, resonances for minor rotamer are enclosed in parenthesis) δ 196.6, 162.9, 154.2, 147.7, 147.4, 128.9, 126.2, 111.4, (110.2) 109.9, 106.3, (105.7), 80.3 (79.8), 57.7, 56.1, (56.0), 51.8, (51.3), 49.0, (39.2), 37.6, 28.5, 28.3; IR (Film): 2975, 1689, 1518, 1419, 1257, 1166 (cm^−1^); HRMS (EI-magnetic sector) *m/z*: {M}^+^ Calcd for C_21_H_29_NO_6_: 391.1995; Found 391.1992.

(±)-*tert*-Butyl 6,7-dimethoxy-1-(2-methoxy-2-oxoethyl)-3,4-dihydroisoquinoline-2(1*H*)-carboxylate (**5j**). Yield 80% as a white foam. ^1^H NMR (400 MHz, CDCl_3_, a 1.5:1 mixture of amide rotamers at room temperature) δ 6.66 (s, 0.4H), 6.65 (s, 0.6H), 6.60 (s, 0.6H), 6.59 (s, 0.4H), 5.54 (t, *J* = 6.0 Hz, 0.4H), 5.46 (t, *J* = 6.4 Hz, 0.6H), 4.14–4.21 (m, 0.6 H), 3.92–3.99 (m, 0.4H), 3.85 (s, 6H), 3.70 (s, 1.8H), 3.68 (s, 1.2H), 3.30–3.35 (m, 0.4H), 3.16–3.23 (m, 0.6H), 2.63–2.92 (5H), 1.48 (s, 9H); ^13^C NMR (100 MHz, CDCl_3_, a rotameric mixture, resonances for minor rotamer are enclosed in parenthesis) δ 170.84, (170.77), (154.0), 153.8, 147.6, 147.2, (128.0), 127.8, 126.1, (125.9), 111.2, (111.1), (109.5), 109.2, 79.9, (79.5), (55.78), 55.75, 55.68, (55.64), 51.7, (51.6), 51.5, 51.1, 41.9, (41.4), (38.5), 37.0, 28.2, 28.0, (27.7); IR (Film): 2971, 1739, 1593, 1517, 1418, 1254, 1166 (cm^−1^); HRMS (EI-magnetic sector) *m/z*: {M}^+^ Calcd for C_19_H_27_NO_6_: 365.1838; Found 365.1836.

(±)-*tert*-Butyl 1-(2-hydroxy-4,6-dimethoxyphenyl)-6,7-dimethoxy-3,4-dihydroisoquinoline-2(1*H*)-carboxylate (**5k**). Yield 75% as a white foam. ^1^H NMR (400 MHz, CDCl_3_) δ 10.19 (brs, 1H), 6.60 (s, 1H), 6.29 (s, 1H), 6.24 (d, *J* = 2.4 Hz, 1H), 6.15 (s, 1H), 5.94 (d, *J* = 2.4 Hz, 1H), 4.12 (dd, *J* = 12.8, 5.6 Hz, 1H), 3.86 (s, 3H), 3.78 (s, 3H), 3.64 (s, 3H), 3.53 (dt, *J* = 12.8, 3.2 Hz, 1H), 3.21 (s, 3H), 2.91 (dt, *J* = 15.6, 5.6 Hz, 1H), 2.67 (dd, *J* = 15.6, 2.8 Hz, 1H), 1.46 (s, 9H); ^13^C NMR (100 MHz, CDCl_3_, a rotameric mixture, resonances for minor rotamer are enclosed in parenthesis) δ 160.7, 159.4, 158.0, 156.3, 147.0, 146.6, 129.0, 125.3, 111.2, 110.6, 108.6, 95.2, 92.8, 81.0, (55.83), 55.76, 55.70, (55.3), 55.2, 55.0, (54.9), 50.1, 39.0, 28.9, 28.3; IR (Film): 3148, 2936, 1644, 1615, 1518, 1428, 1255, 1148 (cm^−1^); HRMS (EI-magnetic sector) *m/z*: {M}^+^ Calcd for C_24_H_31_NO_7_: 445.2101; Found 445.2106.

(±)-*tert*-Butyl 1-(4-(dimethylamino)-2-hydroxyphenyl)-6,7-dimethoxy-3,4-dihydroisoquinoline-2(1*H*)-carboxylate (**5l**). Yield 70% as a colorless oil. ^1^H NMR (400 MHz, CDCl_3_) δ 9.45 (brs, 1H), 6.63 (s, 1H), 6.48 (d, *J* = 8.8 Hz, 1H), 6.40 (s, 1H), 6.36 (d, *J* = 2.4 Hz, 1H), 6.29 (s, 1H), 6.08 (dd, *J* = 8.8, 2.4 Hz, 1H), 3.97 (dd, *J* = 13.6, 5.6 Hz, 1H), 3.88 (s, 3H), 3.70 (s, 3H), 3.13 (ddd, *J* = 12.8, 12.8, 3.6 Hz, 1H), 2.88–2.98 (m, 7H), 2.68 (dd, *J* = 15.6, 2.4 Hz, 1H), 1.47 (s, 9H); ^13^C NMR (100 MHz, CDCl_3_, a rotameric mixture, resonances for minor rotamer are enclosed in parenthesis) δ 156.5, (156.3), 151.4, 147.7, 147.4, 130.7, 126.9, 126.8, 116.2, 111.0, 110.75, 110.71, 103.6, 100.8, 81.3, (55.94), 55.89, 55.85, 52.0, (40.43), 40.38, 40.31, (40.25), 37.4, 28.5, 28.3; IR (Film): 3198, 2976, 1645, 1518, 1432, 1254 (cm^−1^); HRMS (EI-magnetic sector) *m/z*: {M}^+^ Calcd for C_24_H_32_N_2_O_5_: 428.2311; Found 428.2307.

(±)-*tert*-Butyl 1-(4-(diethylamino)phenyl)-6,7-dimethoxy-3,4-dihydroisoquinoline-2(1*H*)-carboxylate (**5m**). Yield 78% as a white foam. ^1^H NMR (400 MHz, CDCl_3_, a 1:1 mixture of amide rotamers at room temperature) δ 7.03 (d, *J* = 8.4 Hz, 2H), 6.94 (s, 1H), 6.56 (d, *J* = 8.4 Hz, 2H), 6.51 (s, 1H), 6.31 (s, 0.5H), 6.12 (s, 0.5H), 4.11 (s, 0.5H), 3.90 (s, 0.5H), 3.88 (s, 3H), 3.75 (s, 3H), 3.31 (q, *J* = 7.2 Hz, 4H), 3.05 (s, 0.5H), 2.91 (s, 0.5H), 2.64 (s, 0.5H), 2.61 (s, 0.5H), 1.5 (s, 9H), 1.14 (t, *J* = 7.2 Hz, 6H); ^13^C NMR (100 MHz, CDCl_3_, a rotameric mixture, resonances for minor rotamer are enclosed in parenthesis) δ 154.1, 147.4, 146.9, 146.5, 129.3, 127.5, 126.9, 110.9, 110.7, 79.4, 56.7, 55.7, 55.6, 44.1, (37.5), 36.1, 28.5, 28.1, (12.53), 12.51; IR (Film): 2974, 1687, 1611, 1519, 1220 (cm^−1^); HRMS (EI-magnetic sector) *m/z*: {M}^+^ Calcd for C_26_H_36_N_2_O_4_: 440.2675; Found 440.2679.

(±)-*tert*-Butyl 1-(1-hydroxynaphthalen-2-yl)-6,7-dimethoxy-3,4-dihydroisoquinoline-2(1*H*)-carboxylate (**5n**). Yield 76% as a white foam. ^1^H NMR (400 MHz, CDCl_3_) δ 10.34 (brs, 1H), 8.44 (dd, *J* = 6.0, 3.2 Hz, 1H), 7.70 (dd, *J* = 7.2, 3.2 Hz, 1H), 7.44–7.49 (m, 2H), 7.17 (d, *J* = 8.8 Hz, 1H), 6.79 (d, *J* = 8.8 Hz, 1H), 6.68 (s, 1H), 6.56 (s, 1H), 6.33 (s, 1H), 4.05 (dd, *J* = 13.6, 5.2 Hz, 1H), 3.90 (s, 3H), 3.63 (s, 3H), 3.21 (td, *J* = 13.2, 3.6 Hz, 1H), 2.99 (td, *J* = 16.0, 5.6 Hz, 1H), 2.75 (dd, *J* = 16.0, 2.4 Hz, 1H), 1.49 (s, 9H); ^13^C NMR (100 MHz, CDCl_3_) δ 156.8, 151.9, 148.0, 147.8, 134.1, 127.2, 127.1, 126.8, 126.7, 126.5, 125.8, 125.0, 123.3, 121.1, 118.5, 111.1, 81.8, 55.99, 55.94, 52.5, 38.1, 28.6; IR (Film): 3134, 2976, 1644, 1518, 1432, 1254, 1159 (cm^−1^); HRMS (EI-magnetic sector) *m/z*: {M}^+^ Calcd for C_26_H_29_NO_5_: 435.2046; Found 435.2048.

(±)-*tert*-Butyl 1-(1*H*-indol-3-yl)-6,7-dimethoxy-3,4-dihydroisoquinoline-2(1*H*)-carboxylate (**5o**). Yield 84% as a white foam. ^1^H NMR (400 MHz, CDCl_3_, a 1.5:1 mixture of amide rotamers at room temperature) δ 8.10 (s, 1H), 7.88 (s, 0.6H), 7.76 (s, 0.4H), 7.34 (d, *J* = 8.0 Hz, 1H), 7.19 (dd, *J* = 8.0, 7.2 Hz, 1H), 7.11 (t, *J* = 7.2 Hz, 1H), 6.63–6.69 (m, 3.6H), 6.49 (brs, 0.4H), 4.03–4.13 (m, 0.4H), 3.89–3.93 (m, 0.6H), 3.89 (s, 3H), 3.73 (s, 3H), 2.95-3.10 (m, 1H), 2.59–2.63 (m, 1H), 1.60 (s, 3.6H), 1.50 (s, 5.4H); ^13^C NMR (100 MHz, CDCl_3_, a rotameric mixture, resonances for minor rotamer are enclosed in parenthesis) δ 154.2, 147.5, 146.8, 136.3, 128.1, 126.7, 126.4, 125.1, 121.9, 120.0, 119.4, 118.5, (118.1), 111.2, 111.0, (80.3), 79.5, 55.9, (51.4), 50.3, 37.6, (36.6), 28.6, 28.2, (27.9); IR (Film): 3360, 2975, 1667, 1517, 1422, 1254 (cm^−1^); HRMS (FAB-magnetic sector) *m/z*: {M}^+^ Calcd for C_24_H_28_N_2_O_4_ 408.2049; Found 408.2047.

(±)-*tert*-Butyl 6,7-dimethoxy-1-(5-methylfuran-2-yl)-3,4-dihydroisoquinoline-2(1*H*)-carboxylate (**5p**). Yield 55% as a white foam. ^1^H NMR (400 MHz, CDCl_3_, a 2:1 mixture of amide rotamers at room temperature) δ 6.63 (s, 1H), 6.62 (s, 1H), 6.24 (s, 0.33H), 6.07 (s, 0.67H), 5.82 (s, 1H), 5.81 (s, 1H), 4.20 (s, 0.67H), 4.05 (s, 0.33H), 3.87 (s, 3H), 3.80 (s, 3H), 3.03–3.31 (m, 1H), 2.84–2.96 (m, 1H), 2.67 (s, 0.67H), 2.63 (s, 0.33H), 2.24 (s, 3H), 1.51 (s, 9H); ^13^C NMR (100 MHz, CDCl_3_, a rotameric mixture, resonances for minor rotamer are enclosed in parenthesis) δ 154.2, 153.2, 151.4, 147.8, 147.0, 126.9, (125.0), 111.2, 111.1, (110.7), 108.9, 105.9, 105.5, 79.7, 55.85, (55.81), 55.7, 52.1, (51.4), (38.7), 37.1, 28.4, 28.1, 13.6; IR (Film): 2976, 1694, 1519, 1415, 1254 (cm^−1^); HRMS (FAB-magnetic sector) *m/z*: {M}^+^ Calcd for C_21_H_27_NO_5_ 373.1889; Found 373.1894.

(±)-Benzyl 1-allyl-6,7-dimethoxy-3,4-dihydroisoquinoline-2(1*H*)-carboxylate (**6a**). Yield 77% as a colorless oil. ^1^H NMR (400 MHz, CDCl_3_, a 1.2:1 mixture of amide rotamers at room temperature) δ 7.30–7.37 (m, 5H), 6.61 (s, 0.45H), 6.59 (s, 0.55H), 6.57 (s, 0.55H), 6.56 (s, 0.45H), 5.82–5.92 (m, 0.45H), 5.71–5.82 (m, 0.55H), 5.16–5.22 (m, 2H), 4.97–5.12 (m, 3H), 4.27 (dd, *J* = 13.2, 3.2 Hz, 0.55H), 4.08–4.11 (m, 0.45H), 3.84 (s, 6H), 3.31–3.38 (m, 0.45H), 3.21–3.28 (m, 0.55H), 2.79–2.94 (m, 1H), 2.65 (dd, *J* = 11.6, 2.4 Hz, 1H), 2.51–2.59 (m, 2H); ^13^C NMR (100 MHz, CDCl_3_, a rotameric mixture, resonances for minor rotamer are enclosed in parenthesis) δ 155.1, 147.5, (147.4), 147.1, (136.7), 136.5, 134.7, (134.6), (128.7), 128.4, 128.2, (127.9), 127.8, 127.7, (127.5), 126.0, (125.7), 117.3, (117.1), 111.4, (111.2), 67.1, (66.9), (55.9), 55.8, 54.0, 41.4, (41.2), (38.3), 37.6, (28.3), 28.0; IR (Film) 2934, 1691, 1516, 1426, 1214 (cm^−1^); HRMS (FAB-magnetic sector) *m/z*: {M+H}^+^ Calcd for C_22_H_26_NO_4_ 368.1862; Found 386.1867.

(±)-Allyl 1-allyl-6,7-dimethoxy-3,4-dihydroisoquinoline-2(1*H*)-carboxylate (**6b**). Yield 86% as a colorless oil. ^1^H NMR (400 MHz, CDCl_3_, a 1.2:1 mixture of amide rotamers at room temperature) δ 6.58–6.61 (m, 2H), 5.95 (ddd, *J* = 16.0, 10.8, 5.6 Hz, 1H), 5.78–5.87 (m, 1H), 5.31 (dd, *J* = 17.2, 6.4 Hz, 0.55H), 5.20–5.22 (m, 1.1H), 5.11 (t, *J* = 6.8 Hz, 0.45H), 5.03–5.07 (m, 2.9H), 4.55–4.67 (m, 2H), 4.25 (dd, *J* = 12.8, 3.6 Hz, 0.55H), 4.01 (dd, *J* = 8.0, 3.6 Hz, 0.45H), 3.85 (s, 6H), 3.34 (dt, *J* = 10.0, 4.0 Hz, 0.45H), 3.23 (dt, *J* = 9.6, 4.0 Hz, 0.55H), 2.81–2.93 (m, 1H), 2.63–2.67 (m, 1H), 2.56–2.57 (m, 2H); ^13^C NMR (100 MHz, CDCl_3_, a rotameric mixture, resonances for minor rotamer are enclosed in parenthesis) δ 155.3, 147.9, (147.8), 147.4, (135.1), 134.9, (133.3), 133.2, (129.1), 128.7, 126.3, (126.0), 117.7, 117.6, (117.4), 117.2, 111.7, (111.5), (110.2), 110.0, 66.3, (66.2), 56.3, 56.2, 54.3, 41.8, (41.5), (38.5), 37.9, (28.6), 28.3; IR (Film) 2934, 1691, 1516, 1431, 1256, 1214 (cm^−1^); HRMS (EI-magnetic sector) *m/z*: {M}^+^ Calcd for C_18_H_23_NO_4_ 317.1627; Found 317.1623.

(±)-Methyl 1-allyl-6,7-dimethoxy-3,4-dihydroisoquinoline-2(1*H*)-carboxylate (**6c**). Yield 60% as a colorless oil. ^1^H NMR (400 MHz, CDCl_3_, a 1.2:1 mixture of amide rotamers at room temperature) δ 6.58–6.61 (m, 2H), 5.78–5.90 (m, 1H), 5.19 (t, *J* = 9.6 Hz, 0.45H), 5.02–5.06 (m, 2.55H), 4.23–4.25 (m, 0.45H), 4.01–4.02 (m, 0.55H), 3.85 (s, 6H), 3.71 (s, 3H), 3.20–3.33 (m, 1H), 2.82–2.93 (m, 1H), 2.66 (t, *J* = 3.6 Hz, 0.55H), 2.62 (t, *J* = 3.6 Hz, 0.45H), 2.53–2.55 (m, 2H); ^13^C NMR (100 MHz, CDCl_3_, a rotameric mixture, resonances for minor rotamer are enclosed in parenthesis) δ 155.7, 147.4, (147.3), 147.0, 134.7, (134.5), 128.7, (128.4), 125.9, (125.6), 117.1, (117.0), 111.3, (111.1), 109.8, (109.6), 55.9, 55.8, 53.8, 52.4, 41.3, (41.0), (38.1), 37.4, 28.1, (27.8); IR (Film) 2953, 1699, 1520, 1449, 1258, 1220 (cm^−1^); HRMS (EI-magnetic sector) *m/z*: {M}^+^ Calcd for C_17_H_21_NO_4_ 291.1471; Found 291.1466.

(±)-Ethyl 1-allyl-6,7-dimethoxy-3,4-dihydroisoquinoline-2(1*H*)-carboxylate (**6d**). Yield 84% as a colorless oil. ^1^H NMR (400 MHz, CDCl_3_, a 1.5:1 mixture of amide rotamers at room temperature) δ 6.58 (brs, 2H), 5.82–5.86 (m, 1H), 5.19 (t, *J* = 7.2 Hz, 0.4H), 5.07–5.10 (m, 0.6H), 5.02–5.07 (m, 2H), 4.03–4.26 (m, 3H), 3.85 (s, 6H), 3.30 (dt, *J* = 11.2, 2.8 Hz, 0.4H), 2.86 (dt, *J* = 12.4, 4.0 Hz, 0.6H), 2.66 (t, *J* = 2.8 Hz, 0.6H), 2.62 (t, *J* = 3.2 Hz, 0.4H), 2.54 (brs, 2H), 1.28 (t, *J* = 6.4 Hz, 3H); ^13^C NMR (100 MHz, CDCl_3_, a rotameric mixture, resonances for minor rotamer are enclosed in parenthesis) δ 155.1, 147.2, (147.1), 146.8, (134.7), 134.4, (128.6), 128.3, 125.8, (125.5), 117.0, (116.7), 111.1, (110.9), (109.6), 109.3, 60.99, (60.97), (55.7), 55.6, 53.5, 41.2, 40.9, (37.8), 37.0, (28.0), 27.8, 14.6; IR (Film) 2934, 1689, 1516, 1427, 1215, 1098 (cm^−1^); HRMS (EI-magnetic sector) *m/z*: {M}^+^ Calcd for C_17_H_23_NO_4_ 305.1627; Found 305.1625.

(±)-1-(1-Allyl-6,7-dimethoxy-3,4-dihydroisoquinolin-2(1*H*)-yl)ethanone (**6e**). Yield 48% as a colorless oil. ^1^H NMR (400 MHz, CDCl_3_, a 1.5:1 mixture of amide rotamers at room temperature) δ 6.62 (s, 0.6H), 6.61(s, 0.4H), 6.592 (s, 0.4H), 6.585 (s, 0.6H), 5.80–5.90 (m, 0.6H), 5.61–5.64 (m, 0.4H), 5.13–5.17 (m, 0.8H), 5.00–5.04 (m, 1.2H), 4.76 (dd, *J* = 9.2, 5.2 Hz, 0.6H), 4.71 (dd, *J* = 8.4, 4.8 Hz, 0.4H), 3.87 (s, 1.2H), 3.86 (s, 1.2H), 3.85 (s, 1.8H), 3.84 (s, 1.8H), 3.79 (ddd, *J* = 8.8, 5.6, 3.6 Hz, 0.4H), 3.53 (ddd, *J* = 14.8, 13.2, 4.4 Hz, 0.6H), 3.04 (dt, *J* = 12.0, 4.4 Hz, 0.4H), 2.87 (dt, *J* = 10.8, 5.6 Hz, 1H), 2.77 (t, *J* = 4.0 Hz, 0.6H), 2.73 (t, *J* = 4.0 Hz, 0.4H), 2.49–2.67 (m, 3H), 2.16 (s, 3H); ^13^C NMR (100 MHz, CDCl_3_, a rotameric mixture, resonances for minor rotamer are enclosed in parenthesis) δ (168.7), 168.5, (147.4), 147.1, 146.9, (146.8), 134.6, (133.5), 128.5, (127.8), (125.9), 124.9, (118.2), 116.6, (111.1), 110.7, 109.7, (109.2), (56.6), (55.7), 55.6, 55.5, 51.0, (41.0), 40.7, 40.3, (34.6), 28.4, (27.4), (21.8), 21.6; IR (Film) 2927, 1632, 1514, 1428, 1255, 1220, 1120 (cm^−1^); HRMS (EI-magnetic sector) *m/z*: {M}^+^ Calcd for C_16_H_21_NO_3_ 275.1521; Found 275.1524.

(±)-(1-Allyl-6,7-dimethoxy-3,4-dihydroisoquinolin-2(1*H*)-yl)(phenyl)methanone (**6f**). Yield 37% as a white foam. ^1^H NMR (400 MHz, CDCl_3_, a 4:1 mixture of amide rotamers at room temperature) δ 7.33–7.42 (m, 5H), 6.69 (s, 0.8H), 6.64 (s, 0.2H), 6.57 (s, 0.8H), 6.38 (s, 0.2H), 5.96–6.06 (m, 0.8H), 5.81 (dd, *J* = 8.8, 4.8 Hz, 0.8H), 5.57–5.65 (m, 0.2H), 5.03–5.13 (m, 2.2H), 4.84 (dd, *J* = 13.6, 6.0 Hz, 0.2H), 4.73–4.76 (m, 0.2H), 3.87 (s, 2.4H), 3.85 (s, 2.4H), 3.79 (s, 1.2H), 3.73–3.77 (m, 1H), 3.45 (dt, *J* = 12.0, 4.0 Hz, 1H), 3.24 (dt, *J* = 12.0, 4.0 Hz, 0.2H), 3.04–3.14 (m, 0.2H), 2.42–2.87 (m, 6.2H); ^13^C NMR (100 MHz, CDCl_3_, a rotameric mixture, resonances for minor rotamer are enclosed in parenthesis) δ (170.7), 170.4, (147.9), 147.6, 136.6, (136.4), 135.0, (133.9), 129.2, (128.5), 128.4, (128.3), (126.9), 126.4, (125.9), 124.9, (118.4), 117.2, (111.6), 111.2, 110.0, (109.3), (57.4), 56.03, 55.93, 50.9, (41.8), 41.5, 41.2, (35.4), 29.1, (27.8); IR (Film) 2933, 1626, 1515, 1428, 1255, 1223, 1118 (cm^−1^); HRMS (EI-magnetic sector) *m/z*: {M}^+^ Calcd for C_21_H_23_NO_3_ 337.1678; Found 337.1679.

(±)-1-Allyl-6,7-dimethoxy-2-(methylsulfonyl)-1,2,3,4-tetrahydroisoquinoline (**6g**). Yield 89% as a white foam. ^1^H NMR (400 MHz, CDCl_3_) δ 6.59 (s, 1H), 6.57 (s, 1H), 5.85–5.95 (m, 1H), 5.13 (s, 1H), 5.10 (d, *J* = 5.6 Hz, 1H), 4.81 (dd, *J* = 8.0, 5.6 Hz, 1H), 3.94 (dd, *J* = 14.4, 6.4 Hz, 1H), 3.86 (s, 3H), 3.85 (s, 3H), 3.46 (ddd, *J* = 16.8, 12.0, 4.8 Hz, 1H), 2.98 (ddd, *J* = 17.2, 12.0, 6.8, 1H), 2.77 (s, 3H), 2.65–2.70 (m, 1H), 2.52–2.62 (m, 2H); ^13^C NMR (100 MHz, CDCl_3_) δ 147.9, 147.4, 134.6, 127.6, 124.5, 117.7, 111.6, 109.7, 56.0, 55.9, 55.6, 41.8, 40.1, 38.8, 26.7; IR (Film) 2935, 1611, 1516, 1316, 1247, 1163, 1120 (cm^−1^); HRMS (EI-magnetic sector) *m/z*: {M}^+^ Calcd for C_15_H_21_NO_4_S 311.1189; Found 311.1191.

(±)-1-Allyl-6,7-dimethoxy-2-tosyl-1,2,3,4-tetrahydroisoquinoline (**6h**). Yield 81% as a colorless oil. ^1^H NMR (400 MHz, CDCl_3_) δ 7.61 (d, *J* = 8.4 Hz, 2H), 7.14 (d, *J* = 8.4 Hz, 2H), 6.53 (s, 1H), 6.38 (s, 1H), 5.83 (dddd, *J* = 17.6, 10.4, 7.2, 7.2 Hz, 1H), 5.05 (d, *J* = 10.4 Hz, 1H), 5.04 (d, *J* = 17.6 Hz, 1H), 4.97 (t, *J* = 6.8 Hz, 1H), 3.85 (s, 3H), 3.81–3.83 (m, 1H), 3.78 (s, 3H), 3.43 (ddd, *J* = 16.4, 10.8, 5.6 Hz, 1H), 2.41–2.60 (m, 4H), 2.34 (s, 3H); ^13^C NMR (100 MHz, CDCl_3_) δ 147.7, 147.2, 143.0, 137.9, 134.6, 129.3, 127.7, 127.0, 124.9, 117.6, 111.3, 109.7, 56.1, 56.0, 55.9, 42.2, 39.2, 26.4, 21.6; IR (Film) 2935, 1517, 1325, 1228, 1157 (cm^−1^); HRMS (EI-magnetic sector) *m/z*: {M}^+^ Calcd for C_21_H_25_NO_4_S 387.1504; Found 387.1504.

(±)-1-Allyl-6,7-dimethoxy-2-((2-nitrophenyl)sulfonyl)-1,2,3,4-tetrahydroisoquinoline (**6i**). Yield 72% as a colorless oil. ^1^H NMR (400 MHz, CDCl_3_) δ 7.95(dd, *J* = 7.6, 1.6 Hz, 1 H), 7.53–7.64 (m, 3H), 6.60 (s, 1H), 6.48 (s, 1H), 5.73 (ddd, *J* = 17.2, 10.0, 7.2 Hz, 1H), 5.02 (d, *J* = 16.8 Hz, 1H), 5.00 (d, *J* = 10.0 Hz, 1H), 4.94 (d, *J* = 9.6 Hz, 1H), 4.04 (dd, *J* = 12.8, 5.6 Hz, 1H), 3.86 (s, 3H), 3.81 (s, 3H), 3.53 (ddd, *J* = 14.8, 12.0, 4.8 Hz, 1H), 2.76 (ddd, *J* = 16.8, 12.0, 6.4 Hz, 1H), 2.62–2.63 (m, 1H), 2.57 (dd, *J* = 15.2, 7.2 Hz, 2H); ^13^C NMR (100 MHz, CDCl_3_) δ 147.8, 147.7, 147.2, 134.1, 134.0, 133.3, 131.5, 130.3, 127.6, 124.5, 123.9, 117.8, 111.3, 109.6, 56.6, 56.0, 55.8, 41.9, 39.4, 27.2; IR (Film) 2937, 1542, 1518, 1350, 1247, 1163, 1120 (cm^−1^); HRMS (FAB-magnetic sector) *m/z*: {M}^+^ Calcd for C_20_H_22_N_2_O_6_S 418.1199; Found 418.1196.

(±)-*tert*-Butyl 1-allyl-6-methoxy-3,4-dihydroisoquinoline-2(1*H*)-carboxylate (**7a**). Yield 85% as a colorless oil. ^1^H NMR (400 MHz, CDCl_3_, a 1.5:1 mixture of amide rotamers at room temperature) δ 7.03 (d, *J* = 8.0 Hz, 1H), 6.65 (brs, 1H), 5.78–5.88 (m, 1H), 5.17–5.20 (m, 0.4H), 4.99–5.05 (m, 2.6H), 4.16–4.20 (m, 0.6H), 3.92–3.96 (m, 0.4H), 3.78 (s, 3H), 3.26–3.31 (m, 0.4H), 3.14–3.21 (m, 0.6H), 2.80–2.95 (m, 1H), 2.72 (t, *J* = 4.0 Hz, 0.6H), 2.68 (t, *J* = 4.0 Hz, 0.4H), 2.45–2.56 (m, 2H), 1.47 (s, 9H); ^13^C NMR (100 MHz, CDCl_3_, a rotameric mixture, resonances for minor rotamer are enclosed in parenthesis) δ 157.9, 154.6, 135.7, (135.5), 135.1, (129.5), 129.3, (128.2), 127.9, 117.2, (116.9), 113.3, 112.4, (112.0), 79.8, (79.5), 55.3, 54.2, (53.3), 41.9, (41.6), (38.6), 36.8, (29.2), 29.1, 28.6; IR (Film) 2974, 1685, 1418, 1232, 1159 (cm^−1^); HRMS (FAB-magnetic sector) *m/z*: {M+H}^+^ Calcd for C_18_H_26_NO_3_ 304.1913; Found 304.1913.

(±)-*tert*-Butyl 1-allyl-7-methoxy-3,4-dihydroisoquinoline-2(1*H*)-carboxylate (**7b**). Yield 79% as a colorless oil. ^1^H NMR (400 MHz, CDCl_3_, a 1.5:1 mixture of amide rotamers at room temperature) δ 7.00–7.05 (m, 1H), 6.73–6.75 (m, 1H), 6.65 (d, *J* = 2.4 Hz, 1H), 5.79–5.89 (m, 1H), 5.20–5.22 (m, 0.4H), 5.01–5.07 (m, 2.6H), 4.18–4.20 (m, 0.6H), 3.93–3.97 (m, 0.4H), 3.79 (s, 3H), 3.24–3.30 (m, 0.4H), 3.13–3.20 (m, 0.6H), 2.76–2.90 (m, 1H), 2.68 (t, *J* = 4.0 Hz, 0.6H), 2.64 (t, *J* = 4.0 Hz, 0.4H), 2.54 (t, *J* = 7.6 Hz, 2H),1.48 (s, 9H); ^13^C NMR (100 MHz, CDCl_3_, a rotameric mixture, resonances for minor rotamer are enclosed in parenthesis) δ 157.6, (154.7), 154.5, (138.3), 138.1, (135.1), 135.0, 129.8, (129.5), 126.4, (126.2), 117.3, (116.9), 112.6, (112.1), 112.0, 79.8, (79.4), 55.4, 54.8, (53.9), 41.6, (41.3), (38.9), 37.1, 28.6, (28.0), 27.9; IR (Film) 2975, 1686, 1420, 1249, 1159 (cm^−1^); HRMS (FAB-magnetic sector) *m/z*: {M+H}^+^ Calcd for C_18_H_26_NO_3_ 304.1913; Found 304.1907.

(±)-*tert*-Butyl 1-allyl-6,8-dimethoxy-3,4-dihydroisoquinoline-2(1*H*)-carboxylate (**7c**). Yield 98% as a colorless oil. ^1^H NMR (400 MHz, CDCl_3_, a 1.5:1 mixture of amide rotamers at room temperature) δ 6.31 (d, *J* = 2.4 Hz, 0.6H), 6.28 (d, *J* = 2.4 Hz, 0.4H), 6.25 (d, *J* = 2.0 Hz, 0.6H), 6.22 (d, *J* = 2.0 Hz, 0.4H), 5.81–5.94 (m, 1H), 5.40 (dd, *J* = 9.6, 4.0 Hz, 0.4H), 5.20 (dd, *J* = 9.6, 3.2 Hz, 0.6H), 4.94–5.07 (m, 2H), 4.20 (ddd, *J* = 13.2, 6.0, 1.6 Hz, 0.6H), 3.95 (ddd, *J* = 12.8, 6.4, 3.2 Hz, 0.4H), 3.82 (s, 1.8H), 3.78 (s, 4.2H), 3.29 (ddd, *J* = 14.8, 10.4, 4.4 Hz, 0.4H), 3.18 (ddd, *J* = 13.2, 11.6, 4.4 Hz, 0.6H), 2.78–2.93 (m, 1H), 2.59–2.68, m, 2H), 2.28–2.36 (m, 1H), 1.47 (s, 5.4H), 1.45 (s, 3.6H); ^13^C NMR (100 MHz, CDCl_3_, a rotameric mixture, resonances for minor rotamer are enclosed in parenthesis) δ 158.9, (158.8), (156.7), 156.5, 154.7, (136.2), 136.0, 135.9, (119.3), 118.9, 116.3, (115.8), 104.2, (104.1), 96.5, (96.3), 79.6, (79.2), 55.4, 55.3, 50.0, (49.0), 38.8, (38.7), (37.8), 36.1, (29.0), 28.9, 28.6; IR (Film) 2975, 1686, 1420, 1249, 1159 (cm^−1^); HRMS (FAB-magnetic sector) *m/z*: {M+H}^+^ Calcd for C_19_H_28_NO_4_ 334.2018; Found 334.2016.

(±)-*tert*-Butyl 1-allyl-7-fluoro-3,4-dihydroisoquinoline-2(1*H*)-carboxylate (**7d**). Yield 89% as a colorless oil. ^1^H NMR (400 MHz, CDCl_3_, a 1.5:1 mixture of amide rotamers at room temperature) δ 7.06–7.07 (m, 1H), 6.82–6.86 (m, 2H), 5.81–5.83 (m, 1H), 5.22 (brs, 0.4H), 5.03–5.06 (m, 2H), 4.21 (d, *J* = 12.0 Hz, 0.6H), 3.97 (d, *J* = 10.4 Hz, 0.4H), 3.25–3.27 (m, 0.4H), 3.14–3.19 (m, 0.6H), 2.83–2.86 (m, 1H), 2.72 (t, *J* = 3.6 Hz, 0.6H), 2.67 (t, *J* = 3.6 Hz, 0.4H), 2.51–2.59 (m, 2H), 1.48 (s, 9H); ^13^C NMR (100 MHz, CDCl_3_, a rotameric mixture, resonances for minor rotamer are enclosed in parenthesis) δ 162.1, 159.7, 138.9, 134.7, 130.4, 130.1, 117.7, (117.4), 113.9, 113.6, (113.4), 54.7, (53.8), 41.6, (41.3), (38.7), 37.1, 28.7, (28.3), 28.2; IR (Film) 2976, 1688, 1413, 1246, 1161, 1114 (cm^−1^); HRMS (FAB-magnetic sector) *m/z*: {M+H}^+^ Calcd for C_17_H_23_FNO_2_ 292.1713; Found 292.1713.

(±)-*tert*-Butyl 1-allyl-7-bromo-3,4-dihydroisoquinoline-2(1*H*)-carboxylate (**7e**). Yield 85% as a white foam. ^1^H NMR (400 MHz, CDCl_3_, a 1.5:1 mixture of amide rotamers at room temperature) δ 7.27 (brs, 2H), 6.98–7.00 (m, 1H), 5.76–5.85 (m, 1H), 5.21 (m, 0.4H), 5.04–5.08 (m, 2.6H), 4.19–4.22 (m, 0.6H), 3.96–3.99 (m, 0.4H), 3.23–3.28 (m, 0.4H), 3.11–3.18 (m, 0.6H), 2.81–2.89 (m, 1H), 2.70 (t, *J* = 3.2 Hz, 0.6H), 2.66 (t, *J* = 3.2Hz, 0.4H), 2.52 (d, *J* = 8.0 Hz, 2H), 1.47 (s, 9H); ^13^C NMR (100 MHz, CDCl_3_, a rotameric mixture, resonances for minor rotamer are enclosed in parenthesis) δ (154.6), 154.5, (139.5), 139.3, 134.6, 133.5, (133.2), 130.7, (130.4), (130.0), 129.8, 129.6, 119.5, 117.7, (117.4), 41.6, (41.3), (38.4), 36.7, 28.7, (28.5), 28.3; IR (Film) 2975, 1687, 1412, 1230, 1159 (cm^−1^); HRMS (FAB-magnetic sector) *m/z*: {M+H}^+^ Calcd for C_17_H_23_BrNO_2_ 352.0912; Found 352.0915.

(±)-*tert*-Butyl 1-allyl-3,4-dihydroisoquinoline-2(1*H*)-carboxylate (**7f**). Yield 92% as a colorless oil. ^1^H NMR (400 MHz, CDCl_3_, a 1.5:1 mixture of amide rotamers at room temperature) δ 7.10–7.15 (m, 4H), 5.81–5.87 (m, 1H), 5.24 (brs, 0.4H), 5.01–5.06 (m, 2.6H), 4.18–4.21 (m, 0.6H), 3.94 (s, 0.4H), 3.17–3.31 (m, 1H), 2.89–2.91 (m, 1H), 2.75 (t, *J* = 4.0 Hz, 0.6H), 2.71 (t, *J* = 4.0 Hz, 0.4H), 2.54 (t, *J* = 7.2 Hz, 2H), 1.47 (s, 9H); ^13^C NMR (100 MHz, CDCl_3_, a rotameric mixture, resonances for minor rotamer are enclosed in parenthesis) δ 154.4, 136.9, 134.93, 134.85, 134.2, (134.0), 128.8, (128.5), 126.8, (126.3), 125.7, 117.1, (116.7), 79.5, (79.2), 54.5, (53.7), 41.6, (41.3), (38.5), 36.9, 28.5; IR (Film) 2978, 1694, 1422, 1166, 1124 (cm^−1^); HRMS (FAB-magnetic sector) *m/z*: {M+H}^+^ Calcd for C_17_H_24_NO_2_ 274.1807; Found 274.1807.

### Synthesis of (±)-1-Allyl-6,7-Dimethoxy-1,2,3,4-Tetrahydroisoquinoline (8)

To a stirred solution of (±)–**5a** (100.0 mg, 0.30 mmol) in DCM (3.0 mL) was added TFA (0.69 mL, 3.0 mmol) at room temperature. The reaction mixture was stirred for 2 h at room temperature under argon atmosphere and then quenched with saturated NaHCO_3_ (5 mL) and the layers were separated and the aqueous layer was extracted with EtOAc (2 × 20 mL). The combined organic layer was washed with brine (5 mL), dried over anhydrous Na_2_SO_4_, filtered, and concentrated *in vacuo*. The residue was purified by column chromatography (silica gel, EtOAc/MeOH/Et_3_N = 15:1:0.1) to afford (±)–**8** (61.6 mg, 0.26 mmol) as a colorless oil.

To a stirred solution of (±)–**6c** (87.4 mg, 0.30 mmol) in ethylene glycol/H_2_O [3.0 mL, 1:1 (v/v)] was added KOH (168.3 mg, 3.0 mmol) at room temperature. The reaction mixture was heated at reflux for 12 h under argon atmosphere and cooled to room temperature and then quenched with saturated NH_4_Cl (5 mL) and the layers were separated and the aqueous layer was extracted with EtOAc (2 × 20 mL). The combined organic layer was washed with brine (5 mL), dried over anhydrous Na_2_SO_4_, filtered, and concentrated *in vacuo*. The residue was purified by column chromatography (silica gel, EtOAc/MeOH/Et_3_N = 15:1:0.1) to afford (±)-**8** (49.0 mg, 0.21 mmol) as a colorless oil.

To a stirred solution of (±)–**6h** (125.5 mg, 0.30 mmol) in DMF (3.0 mL) was added PhSH (0.09 mL, 0.90 mmol) and K_2_CO_3_ (124.4 mg, 0.90 mmol) at room temperature. The reaction mixture was stirred for 12 h at room temperature under argon atmosphere and then quenched with saturated NaHCO_3_ (5 mL) and the layers were separated and the aqueous layer was extracted with EtOAc (2 × 20 mL). The combined organic layer was washed with brine (5 mL), dried over anhydrous Na_2_SO_4_, filtered, and concentrated *in vacuo*. The residue was purified by column chromatography (silica gel, EtOAc/MeOH/Et_3_N = 15:1:0.1) to afford (±)–**8** (61.6 mg, 0.26 mmol) as a colorless oil. ^1^H NMR (400MHz, CDCl_3_) δ 6.64 (s, 1H), 6.56 (s, 1H), 5.83 (dddd, *J* = 16.8, 10.4, 7.6, 6.8 Hz, 1H), 5.12–5.20 (m, 2H), 3.99 (dd, *J* = 8.8, 3.6 Hz, 1H), 3.84 (s, 6H), 3.22 (ddd, *J* = 12.4, 4.8, 4.8 Hz, 1H), 2.95 (ddd, *J* = 12.4, 7.6, 4.8 Hz, 1H), 2.72–2.79 (m, 1H), 2.60–2.70 (m, 2H), 2.45–2.53 (m, 1H); ^13^C NMR (100MHz, CDCl_3_) δ 147.1, 146.9, 135.4, 130.2, 127.2, 117.8, 111.6, 108.9, 56.0, 55.8, 54.7, 41.1, 40.8, 29.5; IR (Film) 2932, 1510, 1464, 1355, 1258, 1112 (cm^−1^); HRMS (EI-magnetic sector) m/z: {M}^+^ Calcd for C_14_H_19_NO_2_ 233.1416; Found 233.1416.

### Total Synthesis of (±)-Benzo[*a*]Quinolizidine (10)

(±)-(*E*)-Benzyl 6,7-dimethoxy-1-(4-methoxy-4-oxobut-2-en-1-yl)-3,4-dihydroisoquinoline-2(1*H*)-carboxylate (**9**). To a stirred solution of **4b** (120.5 mg, 0.37 mmol) in DCM (3.70 mL) was added 4Å molecular sieves (160 mg) at room temperature. After the reaction mixture was stirred for 15 min at room temperature, 2,3-dichloro-5,6-dicyano-1,4-benzoquinone (DDQ) (91.9 mg, 0.40 mmol) was added portionwise and the reaction mixture was stirred at room temperature for 30 min under argon atmosphere. CH_2_=CH(OTMS) (0.14 mL, 0.92 mmol) was added dropwise at room temperature and the reaction mixture was stirred at room temperature for 1 h under argon atmosphere. Ph_3_P=CO_2_Me (213.4 mg, 0.63 mmol) was added portionwise and the reaction mixture was stirred at room temperature for 12 h and then diluted with hexanes (5.0 mL) and concentrated in vacuo. Purification of the crude residue by flash chromatography on silica gel, using hexanes/EtOAc (4:1 to 3:1) as elutant, provided (±)–**8** (124.4 mg, 0.29 mmol, *E/Z* = 95:5) as a white foam. ^1^H NMR (400 MHz, CDCl_3_, a 1:1 mixture of amide rotamers at room temperature) δ 7.29–7.38 (m, 5H), 6.93–7.00 (m, 1H), 6.59 (s, 0.5H), 6.57 (s, 0.5H), 6.56 (s, 0.5H), 6.51 (s, 0.5H), 5.82 (d, *J* = 16.0 Hz, 0.5H), 5.80 (d, *J* = 16.0 Hz, 0.5H), 5.29 (t, *J* = 6.8 Hz, 0.5H), 5.17 (t, *J* = 6.8 Hz, 0.5H), 5.16 (d, *J* = 4.0 Hz, 1H), 5.12 (d, *J* = 4.0 Hz, 1H), 4.24–4.28 (m, 0.5H), 4.02–4.07 (m, 0.5H), 3.85 (s, 3H), 3.83 (s, 1.5H), 3.82 (s, 1.5H), 3.71 (s, 1.5H), 3.70 (s, 1.5H), 3.29–3.36 (m, 0.5H), 3.17–3.25 (m, 0.5H), 2.78–2.94 (m, 1H), 2.64–2.74 (m, 3H);); ^13^C NMR (100 MHz, CDCl_3_, a rotameric mixture, resonances for minor rotamer are enclosed in parenthesis) δ (166.3), 166.2, (155.2), 155.0, 147.8, (147.7), 147.3, (144.9), 144.8, (136.6), 136.2, 128.4, 128.1, (128.0), (127.8), 127.6, 127.4, 126.1 (125.9), 123.24, (123.18), 111.4, (111.3), (109.7), 109.4, 67.6, (67.2), 56.0, (55.9), (53.63), 53.59, 51.5, 39.8, (39.4), (38.8), 37.8, (28.2), 27.9; IR (Film) 2937, 1542, 1517, 1348, 1246, 1162 (cm^−1^); HRMS (FAB-magnetic sector) *m/z*: {M}^+^ Calcd for C_24_H_27_NO_6_ 425.1838; Found 425.1837.

(±)-9,10-Dimethoxy-2,3,6,7-tetrahydro-1*H*-pyrido[2,1-*a*]isoquinolin-4(11b*H*)-one (**9**). To a stirred solution of (±)-**8** (30.5 mg, 0.072 mmol) in EtOAc (2.4 mL) was added 10% Pd/C (3.1 mg) at room temperature. The reaction mixture was stirred under H_2_ atmosphere for 18 h, then filtered through a pad of Celite 545 and concentrated *in vacuo*. Purification of the residue by flash chromatography on silica gel, using hexanes/EtOAc (1:5.5) as elutant, provided (±)–**9** (16.0 mg, 0.061 mmol) as a clear oil. ^1^H NMR (400 MHz, CDCl_3_) δ 6.67 (s, 1H), 6.61 (s, 1H), 4.88 (ddd, *J* = 12.4, 4.4, 2.4 Hz, 1H), 4.61 (dd, *J* = 10.8, 4.4 Hz, 1H), 3.86 (s, 6H), 2.91 (dt, *J* = 12.0, 3.6 Hz, 1H), 2.80 (dt, *J* = 12.0, 2.8 Hz, 1H), 2.50–2.66 (m, 3H), 2.37 (ddd, *J* = 18.0, 12.0, 6.8 Hz, 1H), 1.79–1.92 (m, 1H), 1.79–1.90 (m, 1H) 1.62–1.72 (m, 1H); ^13^C NMR (100 MHz, CDCl_3_) δ 168.7, 147.3, 147.2, 128.7, 126.8, 111.2, 107.9, 56.4, 55.8, 55.6, 39.4, 32.0, 30.7, 28.3, 19.4; IR (Film) 3454, 2936, 1635, 1515, 1257, 1225 (cm^−1^); HRMS (EI-magnetic sector) *m/z*: {M}^+^ Calcd for C_15_H_19_NO_3_ 261.1365; Found 261.1363.

(±)-9,10-Dimethoxy-2,3,4,6,7,11*b*-hexahydro-1*H*-pyrido[2,1-*a*]isoquinoline (**10**). To a stirred solution of (±)–**9** (70.0 mg, 0.268 mmol) in THF (2.4 mL) was LiAlH_4_ (152.5 mg, 4.018 mmol) at 0 °C. The reaction mixture was heated to reflux for 1 h under argon atmosphere, then cooled to room temperature and then quenched with saturated Rochelle's salt (5 mL) and the layers were separated and the aqueous layer was extracted with EtOAc (2 × 20 mL). The combined organic layer was washed with brine (5 mL), dried over anhydrous Na_2_SO_4_, filtered, and concentrated *in vacuo*. The residue was purified by column chromatography (silica gel, hexane/EtOAc/Et_3_N = 1:9:0.1) to afford (±)–**10** (51.0 mg, 0.206 mmol) as a colorless oil. ^1^H NMR (400 MHz, CDCl_3_) δ 6.68 (s, 1H), 6.56 (s, 1H), 3.84 (s, 6H), 3.04–3.15 (m, 2H), 2.91–2.99 (m, 2H), 2.60 (dd, *J* = 16.0, 3.6 Hz, 1H), 2.50 (dt, *J* = 11.2, 4.0 Hz, 1H), 2.32 (dd, *J* = 11.2, 4.0 Hz, 1H), 2.23–2.28 (m, 1H), 1.90–1.95 (m, 1H), 1.66–1.77 (m, 2H), 1.37–1.54 (m, 2H); ^13^C NMR (100 MHz, CDCl_3_) δ 147.3, 147.1, 130.4, 126.8, 111.6, 108.3, 63.4, 57.0, 56.2, 56.0, 53.0, 31.7, 29.3, 25.7, 25.3; IR (Film) 3423, 2933, 1602, 1510, 1259, 1225 (cm^−1^); HRMS (EI-magnetic sector) *m/z*: {M}^+^ Calcd for C_15_H_21_NO_2_ 247.1572; Found 247.1574.

## Data Availability Statement

The raw data supporting the conclusions of this article will be made available by the authors, without undue reservation.

## Author Contributions

DL contributed conception and design of the study. HY and DL have been involved in the synthesis of all compounds with the help of HK. S-HB and DL analyzed the results and wrote the paper. All authors contributed to manuscript revision, read, and approved the submitted version.

## Conflict of Interest

The authors declare that the research was conducted in the absence of any commercial or financial relationships that could be construed as a potential conflict of interest.
