# Supplementary material for: Direct and Efficient C(sp3)–H Functionalization of N-Acyl/Sulfonyl Tetrahydroisoquinolines (THIQs) With Electron-Rich Nucleophiles via 2,3-Dichloro-5,6-Dicyano-1,4-Benzoquinone (DDQ) Oxidation
Source: Front Chem. 2020 Jul 29;8:629. doi: 10.3389/fchem.2020.00629 (PMC7403605; doi:10.3389/fchem.2020.00629)
Supplement: Supplementary file 1 [file Data_Sheet_1.PDF]

Supplementary Material

Direct and Efficient C(sp^3)–H Functionalization of *N*-Acyl/Sulfonyl Tetrahydroisoquinolines (THIQs) with Electron-rich Nucleophiles via 2,3-Dichloro-5,6-dicyano-1,4-benzoquinone (DDQ) Oxidation

Heesun Yu[1], Hyoungsu Kim[1], Seung-Hoon Baek[1]*, Dongjoo Lee[1]*

[1]College of Pharmacy and Research Institute of Pharmaceutical Science and Technology (RIPST), Ajou University, Suwon 16499, Republic of Korea

Correspondence: Seung-Hoon Baek and Dongjoo Lee
(E-mail: shbaek@ajou.ac.kr and dongjoo@ajou.ac.kr)

Table of Contents

List of Substrate Structures and References for their Preparation	4
Compound (±)-**5a**: ^{1}H NMR and ^{13}C NMR	5
Compound (±)-**5b**: ^{1}H NMR and ^{13}C NMR	6
Compound (±)-**5c**: ^{1}H NMR and ^{13}C NMR	7
Compound (±)-**5d**: ^{1}H NMR and ^{13}C NMR	8
Compound (±)-**5e**: ^{1}H NMR and ^{13}C NMR	9
Compound (±)-**5f**: ^{1}H NMR and ^{13}C NMR	10
Compound (±)-**5g**: ^{1}H NMR and ^{13}C NMR	11
Compound (±)-**5h**: ^{1}H NMR and ^{13}C NMR	12
Compound (±)-**5i**: ^{1}H NMR and ^{13}C NMR	13
Compound (±)-**5j**: ^{1}H NMR and ^{13}C NMR	14
Compound (±)-**5k**: ^{1}H NMR and ^{13}C NMR	15
Compound (±)-**5l**: ^{1}H NMR and ^{13}C NMR	16
Compound (±)-**5m**: ^{1}H NMR and ^{13}C NMR	17
Compound (±)-**5n**: ^{1}H NMR and ^{13}C NMR	18
Compound (±)-**5o**: ^{1}H NMR and ^{13}C NMR	19
Compound (±)-**5p**: ^{1}H NMR and ^{13}C NMR	20
Compound (±)-**6a**: ^{1}H NMR and ^{13}C NMR	21
Compound (±)-**6b**: ^{1}H NMR and ^{13}C NMR	22
Compound (±)-**6c**: ^{1}H NMR and ^{13}C NMR	23
Compound (±)-**6d**: ^{1}H NMR and ^{13}C NMR	24
Compound (±)-**6e**: ^{1}H NMR and ^{13}C NMR	25
Compound (±)-**6f**: ^{1}H NMR and ^{13}C NMR	26
Compound (±)-**6g**: ^{1}H NMR and ^{13}C NMR	27

Compound (±)-**6h**: ^{1}H NMR and ^{13}C NMR 28

Compound (±)-**6i**: ^{1}H NMR and ^{13}C NMR 29

Compound (±)-**7a**: ^{1}H NMR and ^{13}C NMR 30

Compound (±)-**7b**: ^{1}H NMR and ^{13}C NMR 31

Compound (±)-**7c**: ^{1}H NMR and ^{13}C NMR 32

Compound (±)-**7d**: ^{1}H NMR and ^{13}C NMR 33

Compound (±)-**7e**: ^{1}H NMR and ^{13}C NMR 34

Compound (±)-**7f**: ^{1}H NMR and ^{13}C NMR 35

Compound (±)-**8**: ^{1}H NMR and ^{13}C NMR 36

Compound (±)-**9**: ^{1}H NMR and ^{13}C NMR 37

Compound (±)-**10**: ^{1}H NMR and ^{13}C NMR 38

Compound (±)-**11**: ^{1}H NMR and ^{13}C NMR 39

Comparison of ^{13}C NMR Data for (±)-Benzo[*a*]quinazoline (**11**) 40

List of Substrate Structures and References for their Preparation

	Substrate	Reference		Substrate	Reference
**4a**	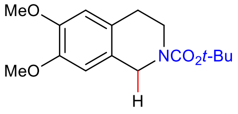	*Chem. Commun.* **2014**, *50*, 1238–1240	**4b**	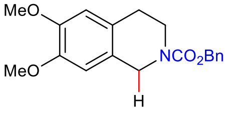	*Chem. Commun.* **2005**, *41*, 4465–4467
**4c**	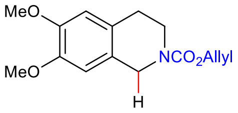	*Molecules* **2018**, *23*, 3223–3235	**4d**	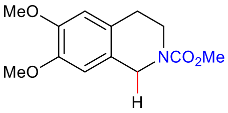	*Bioorg. Med. Chem.* **2004**, *12*, 871–882
**4e**	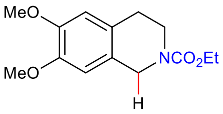	*Helv. Chim. Acta* **1980**, *63*, 938–961	**4f**	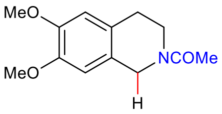	*Bioorg. Med. Chem. Lett.* **2010**, *20*, 4999–5003
**4g**	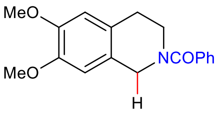	*Synthetic Commun.* **1992**, *22*, 3235–3242	**4h**	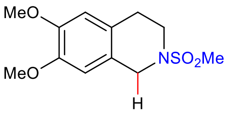	*Tetrahedron Lett.* **2001**, *42*, 6251–6253
**4i**	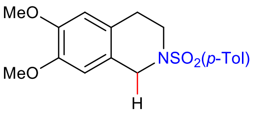	*Tetrahedron Lett.* **2010**, *51*, 435–438	**4j**	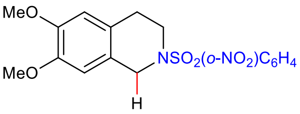	*Molecules* **2018**, *23*, 3223–3235
**4k**	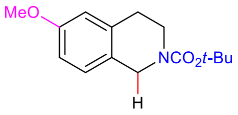	*Adv. Synth. Catal.* **2016**, *358*, 4049–4056	**4l**	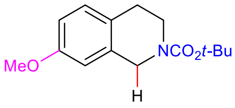	*Bioorg. Med. Chem.* **2008**, *16*, 2499–2512
**4m**	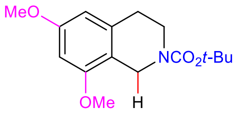	*Adv. Synth. Catal.* **2016**, *358*, 4049–4056	**4n**	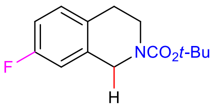	*Molecules* **2018**, *23*, 3223–3235
**4o**	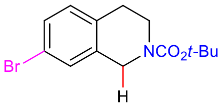	*Bioorg. Med. Chem. Lett.* **2018**, *28*, 3050–3056	**4p**	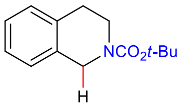	*J. Org. Chem.* **2011**, *76*, 6703–6714

(±)-*tert*-Butyl 1-Allyl-6,7-dimethoxy-3,4-dihydroisoquinoline-2(1*H*)-carboxylate (**5a**)

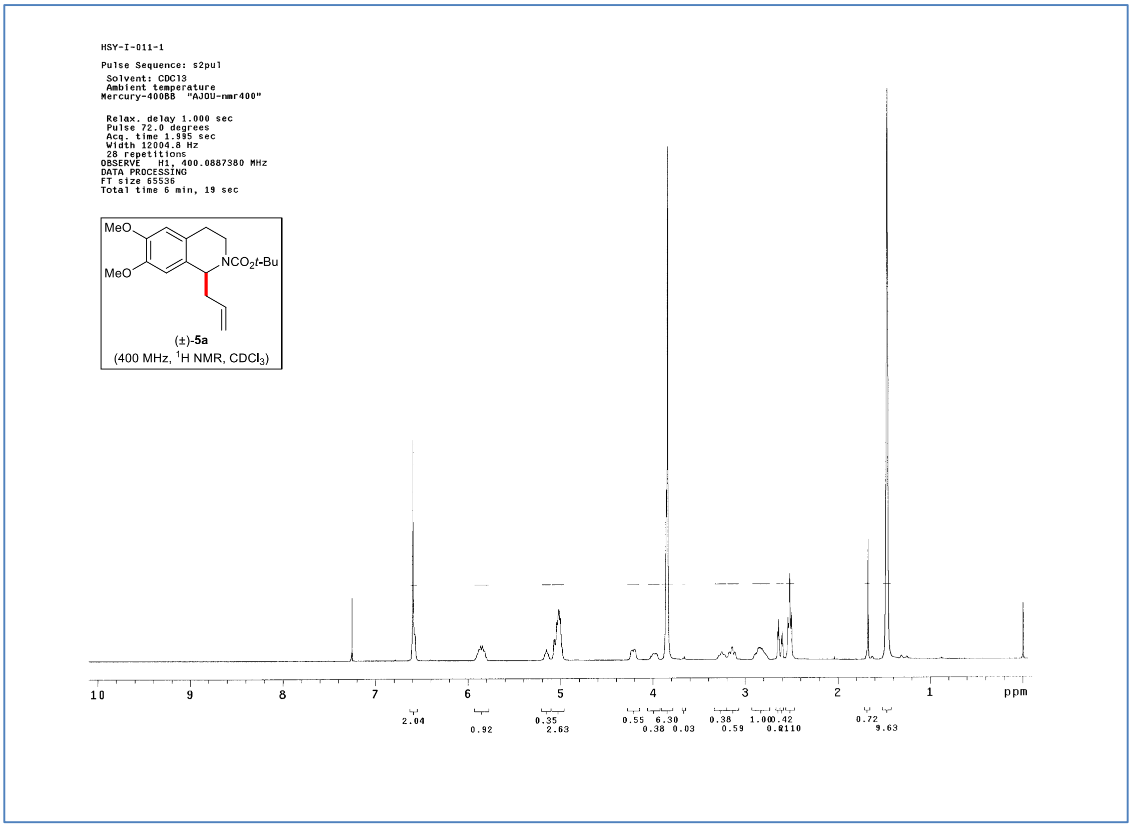

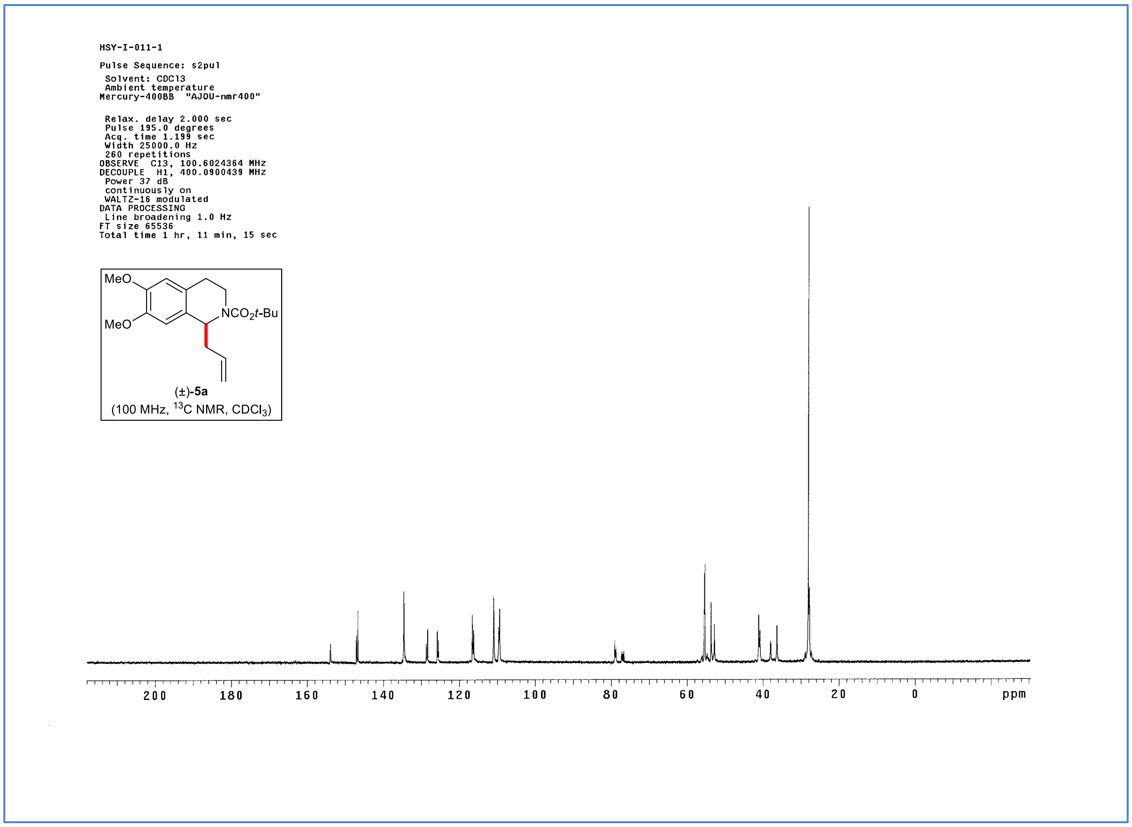

(±)-*tert*-Butyl 6,7-Dimethoxy-1-(2-methylallyl)-3,4-dihydroisoquinoline-2(1*H*)-carboxylate (**5b**)

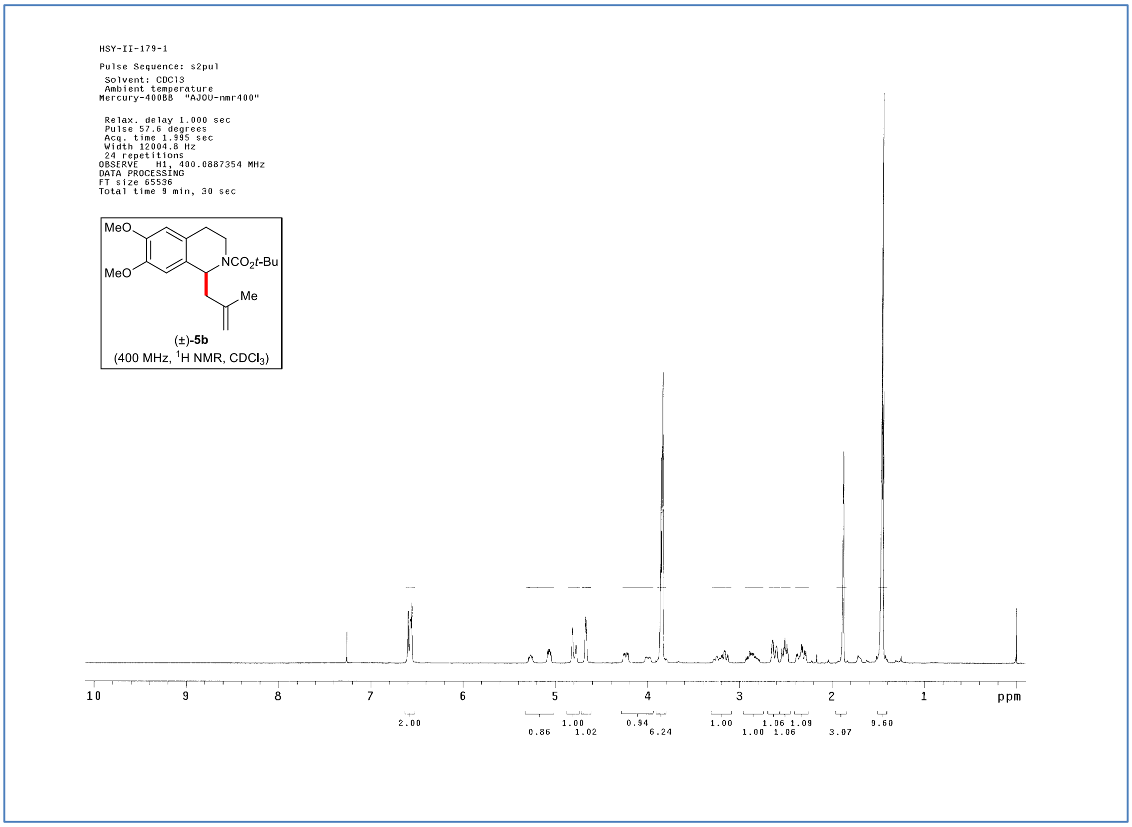

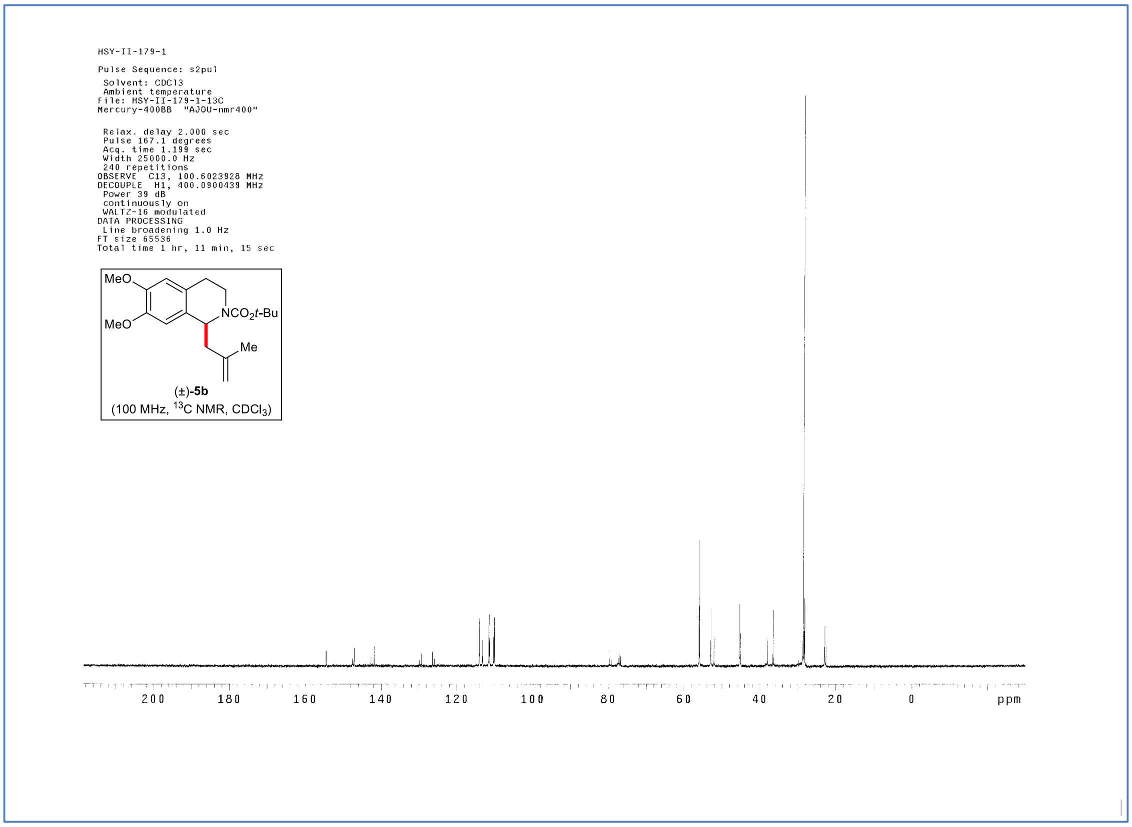

(±)-*tert*-Butyl 6,7-dimethoxy-1-(2-methylbut-3-en-2-yl)-3,4-dihydroisoquinoline-2(1*H*)-carboxylate (**5c**)

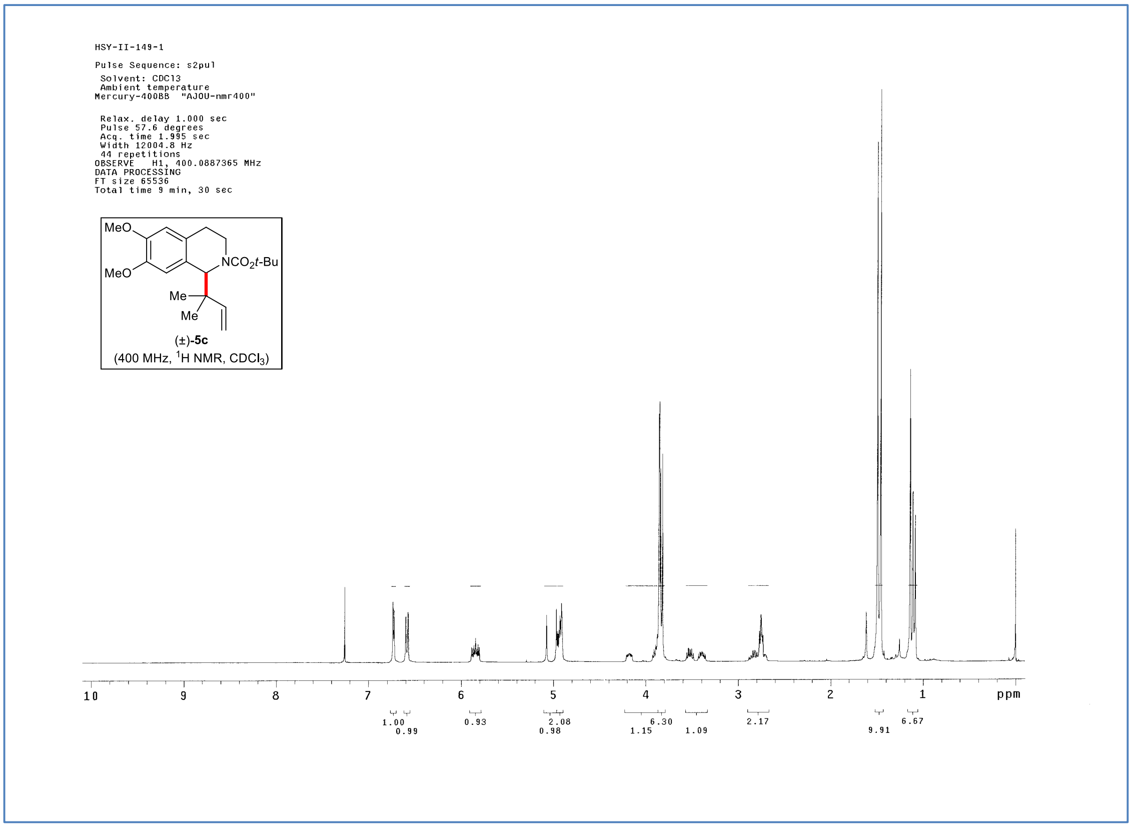

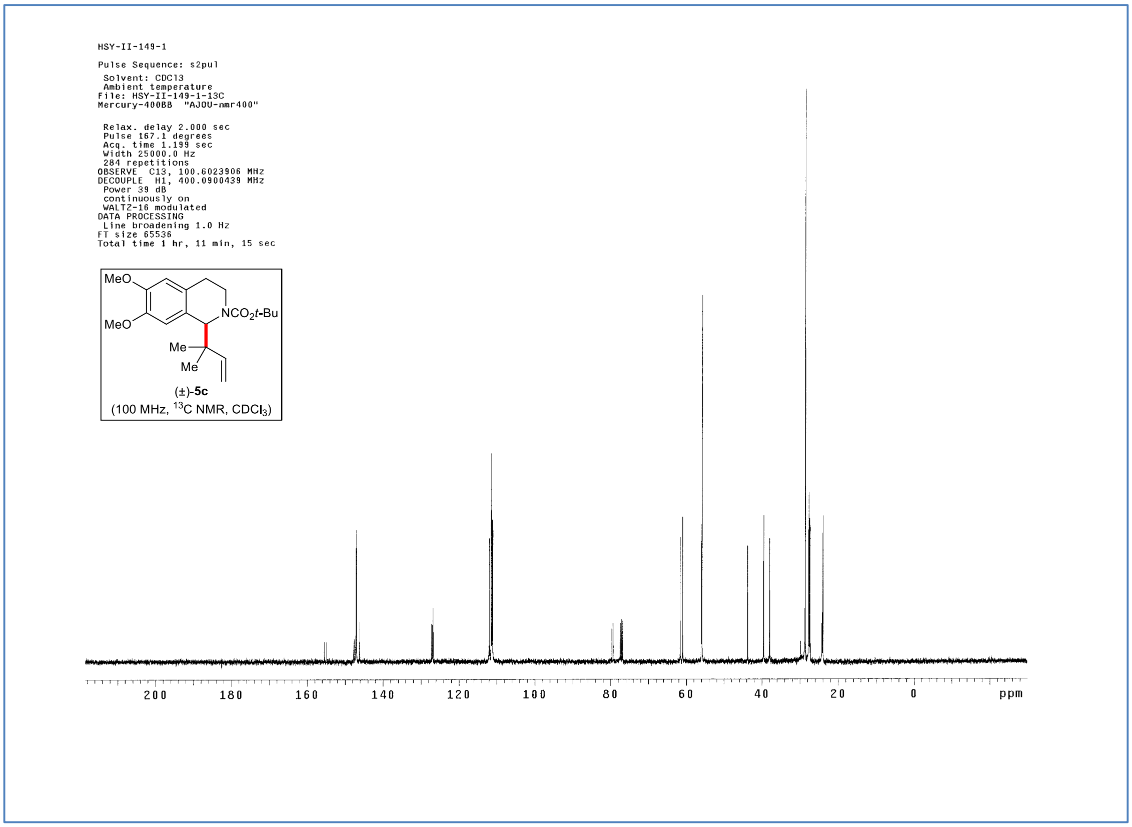

(±)-*tert*-Butyl 6,7-Dimethoxy-1-(prop-2-yn-1-yl)-3,4-dihydroisoquinoline-2(1*H*)-carboxylate (**5d**)

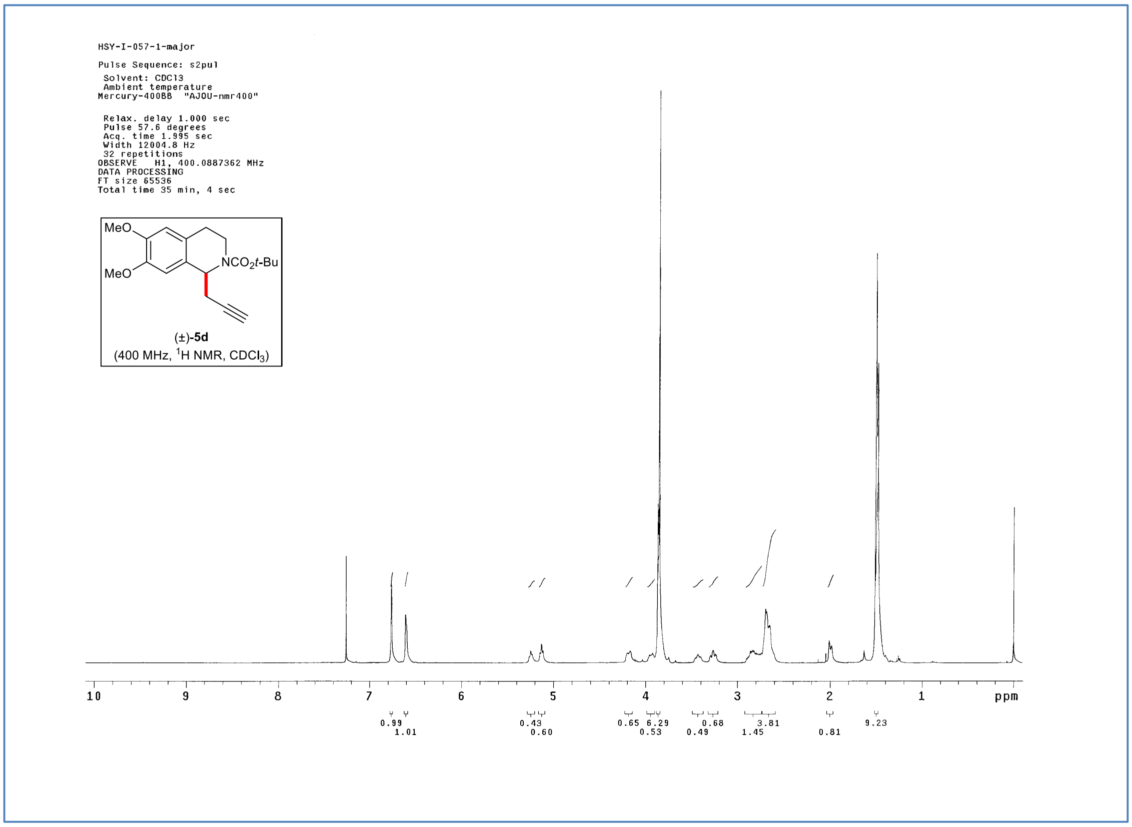

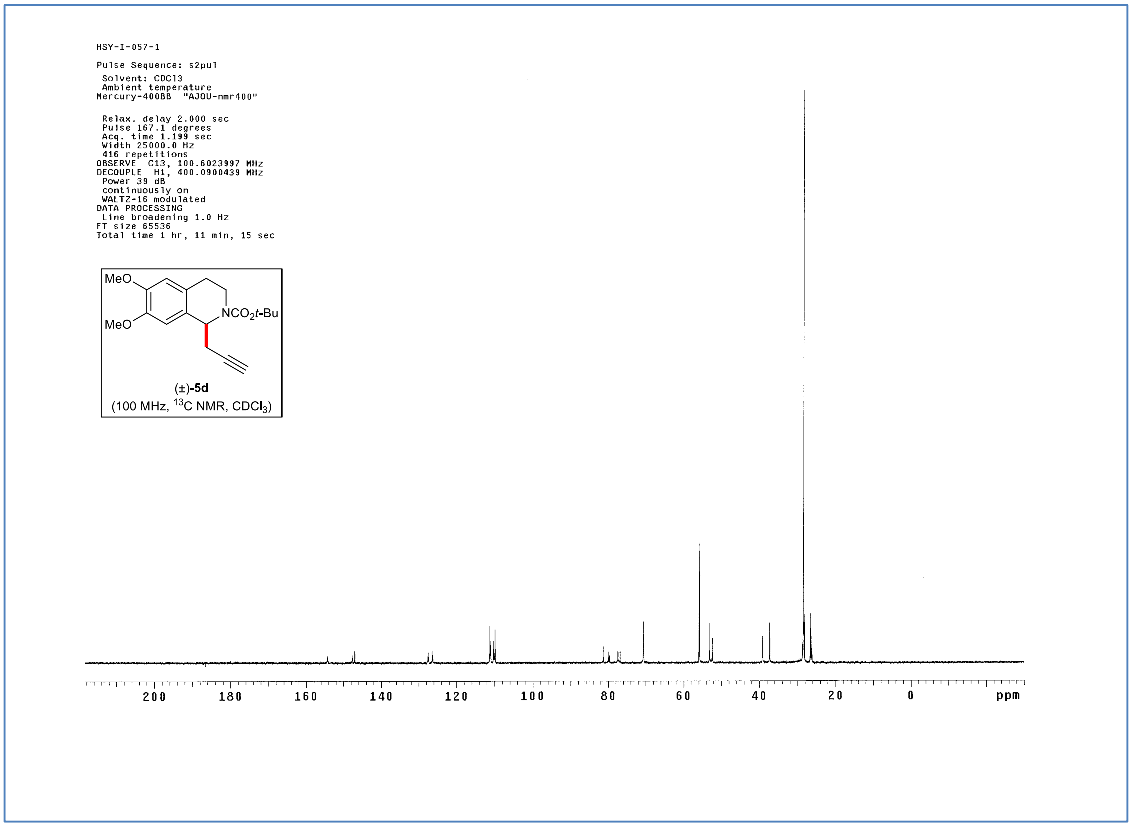

(±)-*tert*-Butyl 6,7-Dimethoxy-1-(2-oxoethyl)-3,4-dihydroisoquinoline-2(1*H*)-carboxylate (**5e**)

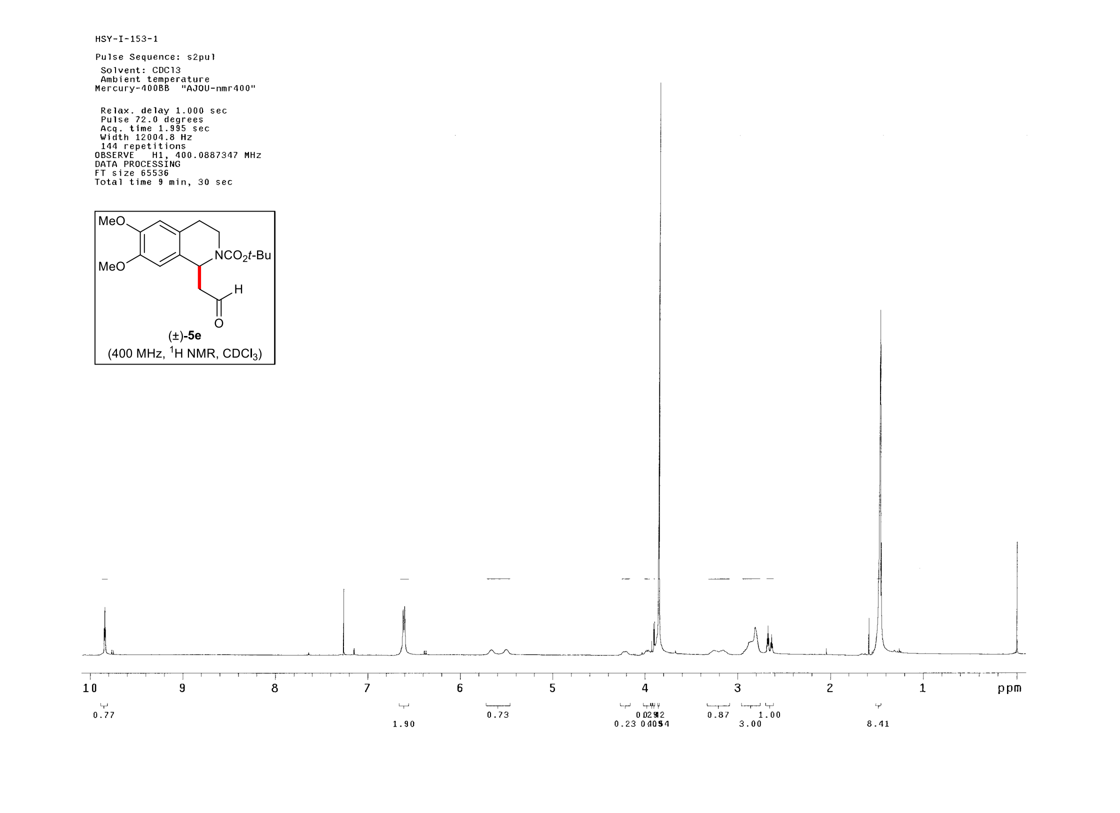

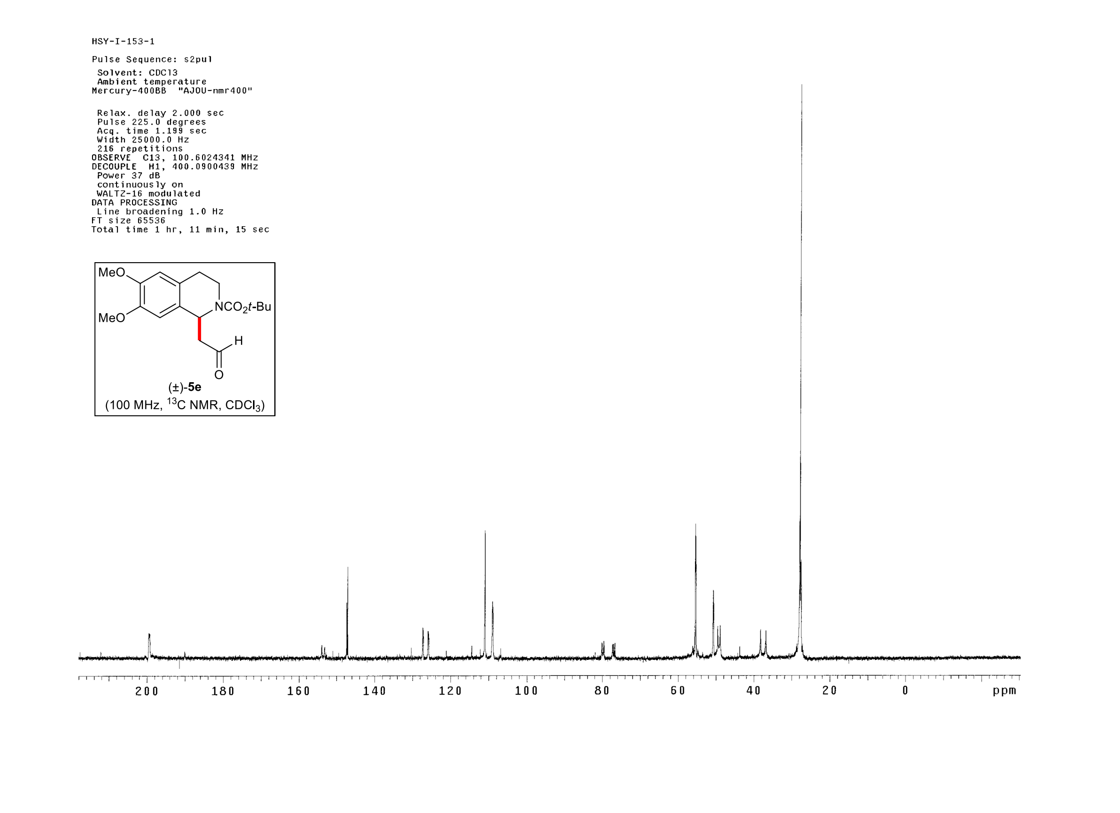

(±)-*tert*-Butyl 6,7-Dimethoxy-1-(2-oxopropyl)-3,4-dihydroisoquinoline-2(1*H*)-carboxylate (**5f**)

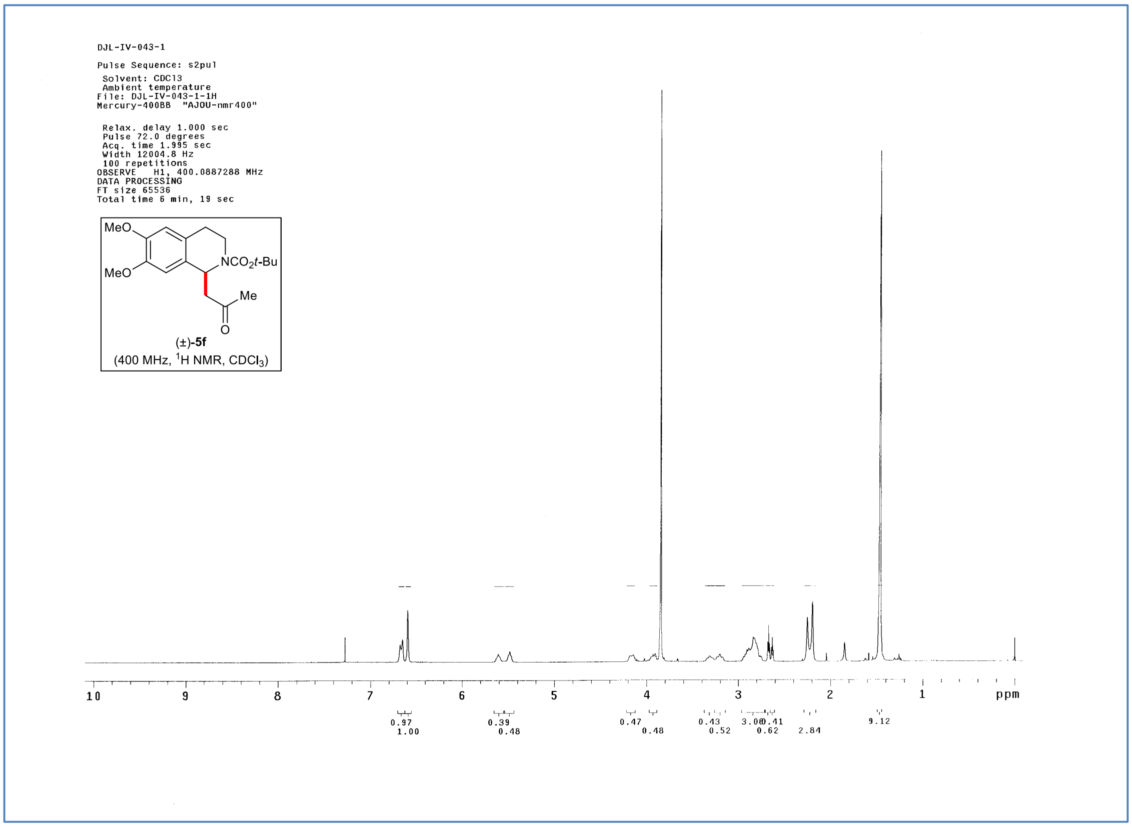

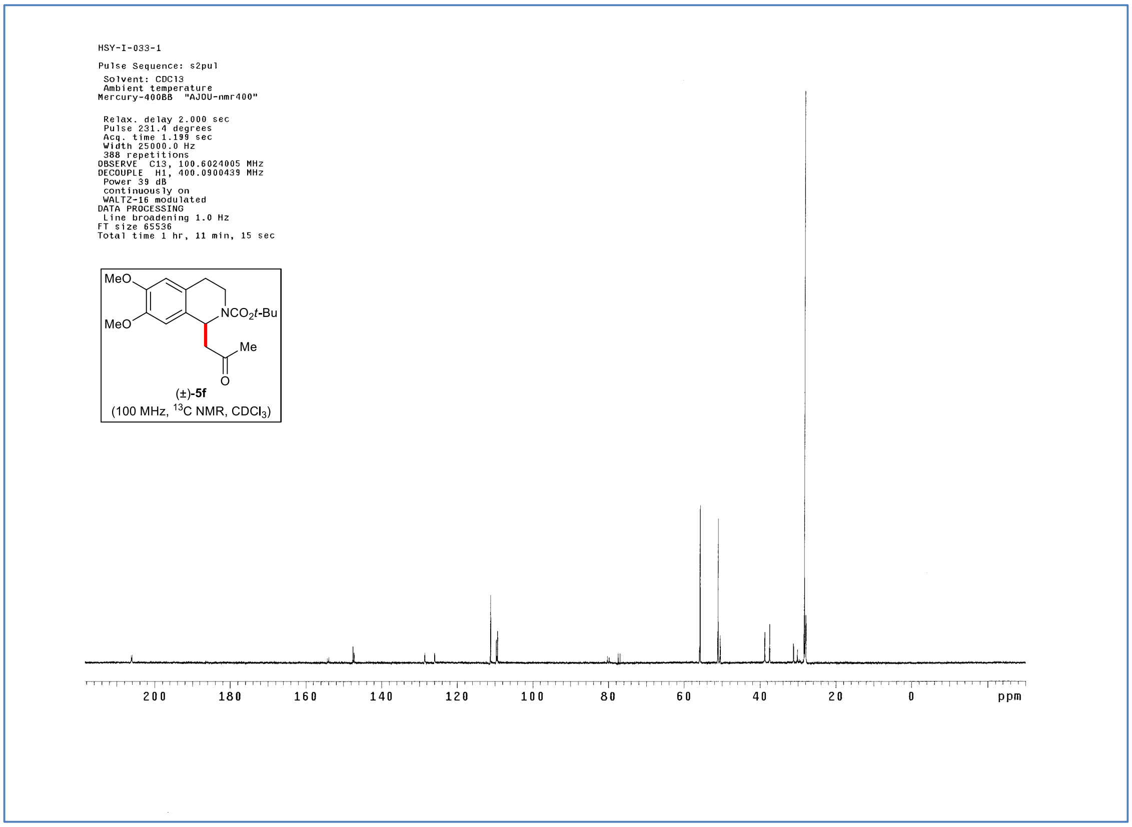

(±)-*tert*-Butyl 1-(3,3-Dimethyl-2-oxobutyl)-6,7-dimethoxy-3,4-dihydroisoquinoline-2(1*H*)-carboxylate (**5g**)

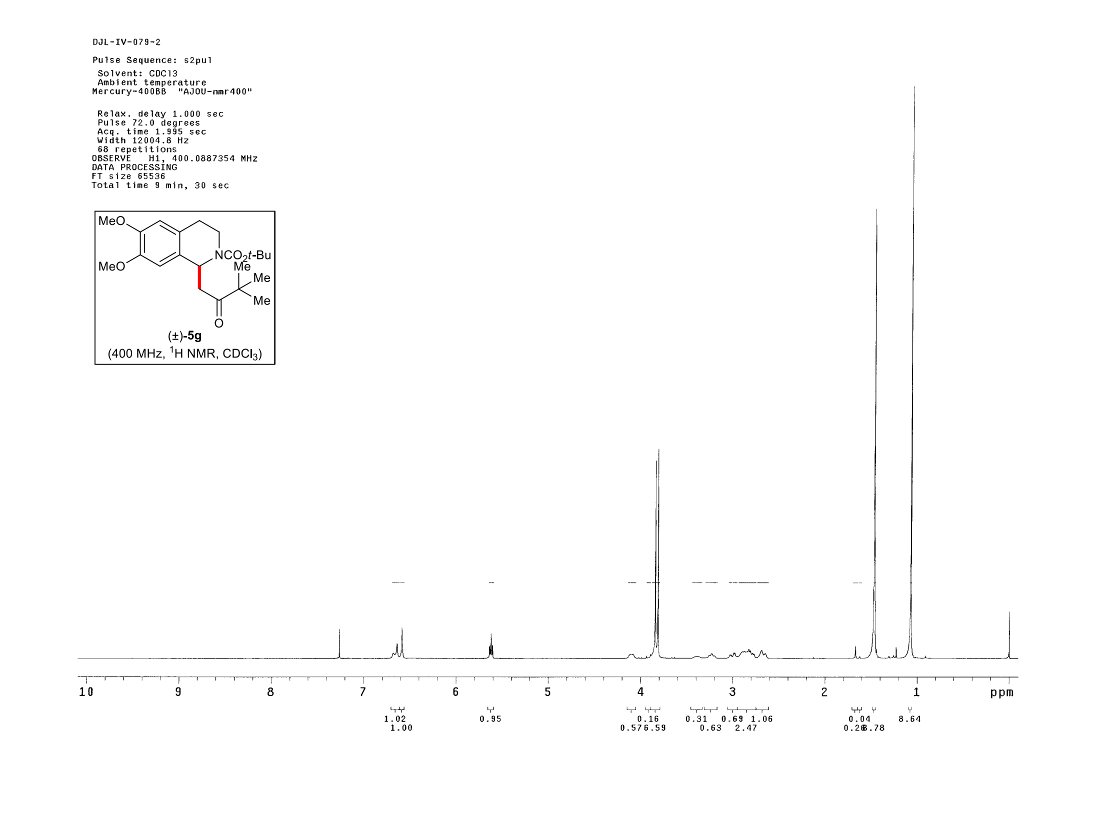

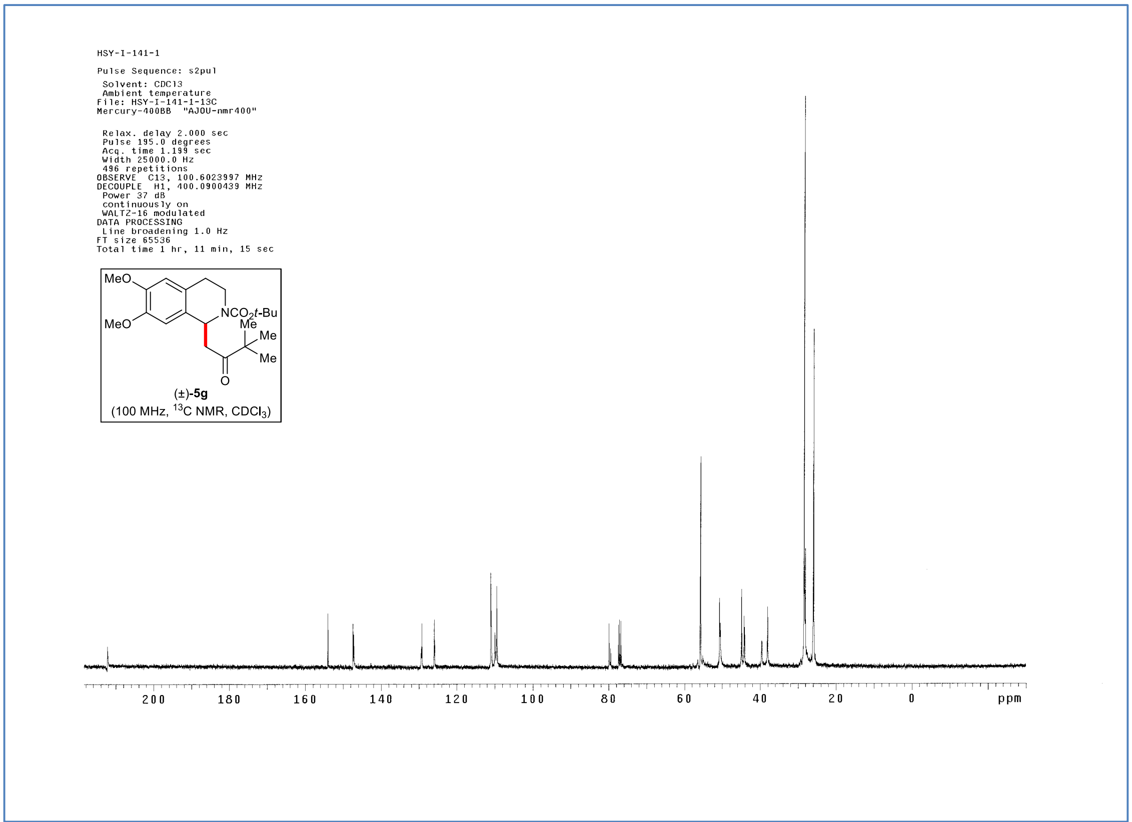

(±)-*tert*-Butyl 6,7-Dimethoxy-1-(2-oxo-2-phenylethyl)-3,4-dihydroisoquinoline-2(1*H*)-carboxylate (**5h**)

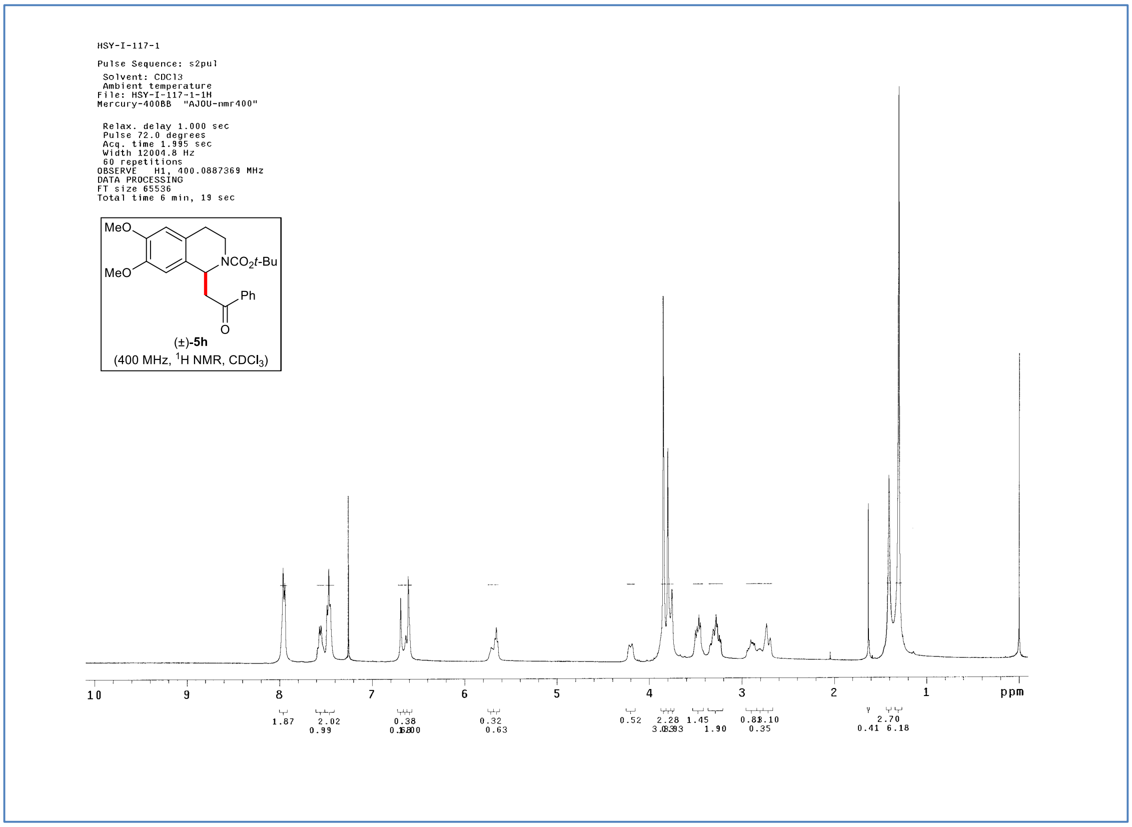

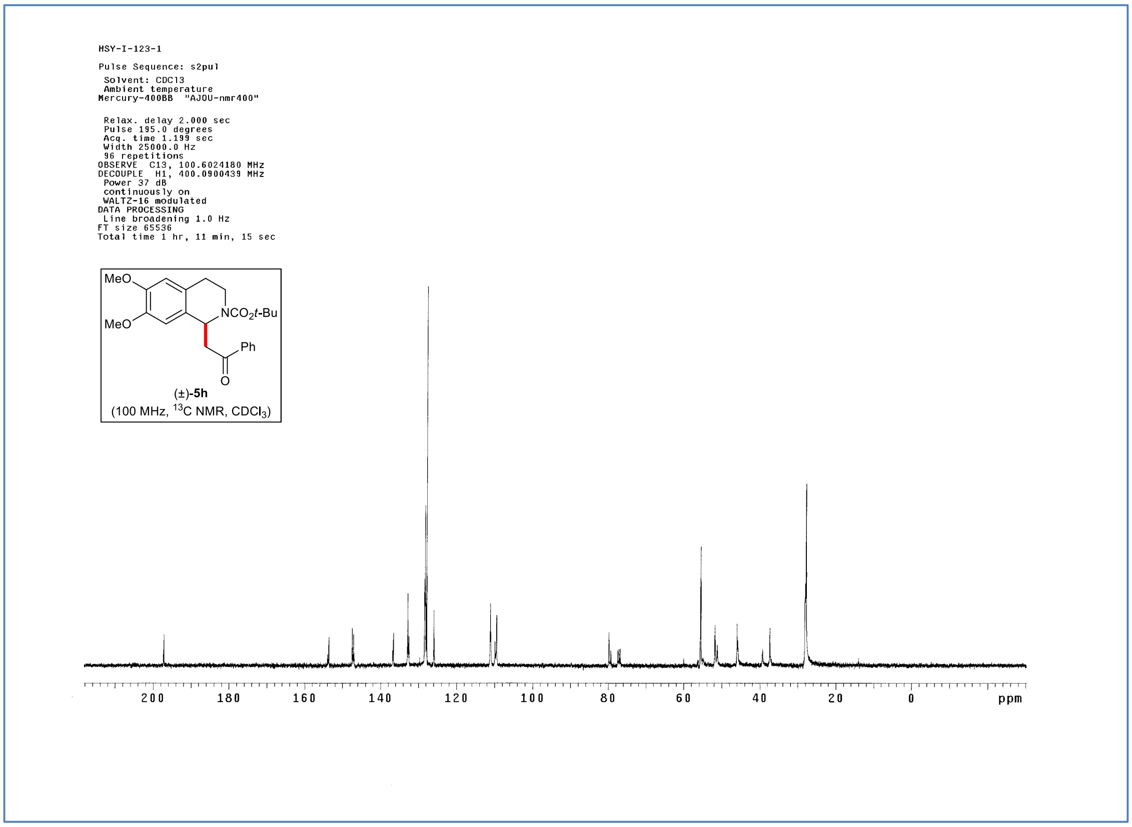

(±)-(*E*)-*tert*-Butyl 6,7-Dimethoxy-1-(4-methoxy-2-oxobut-3-en-1-yl)-3,4-dihydroisoquinoline-2(1*H*)-carboxylate (**5i**)

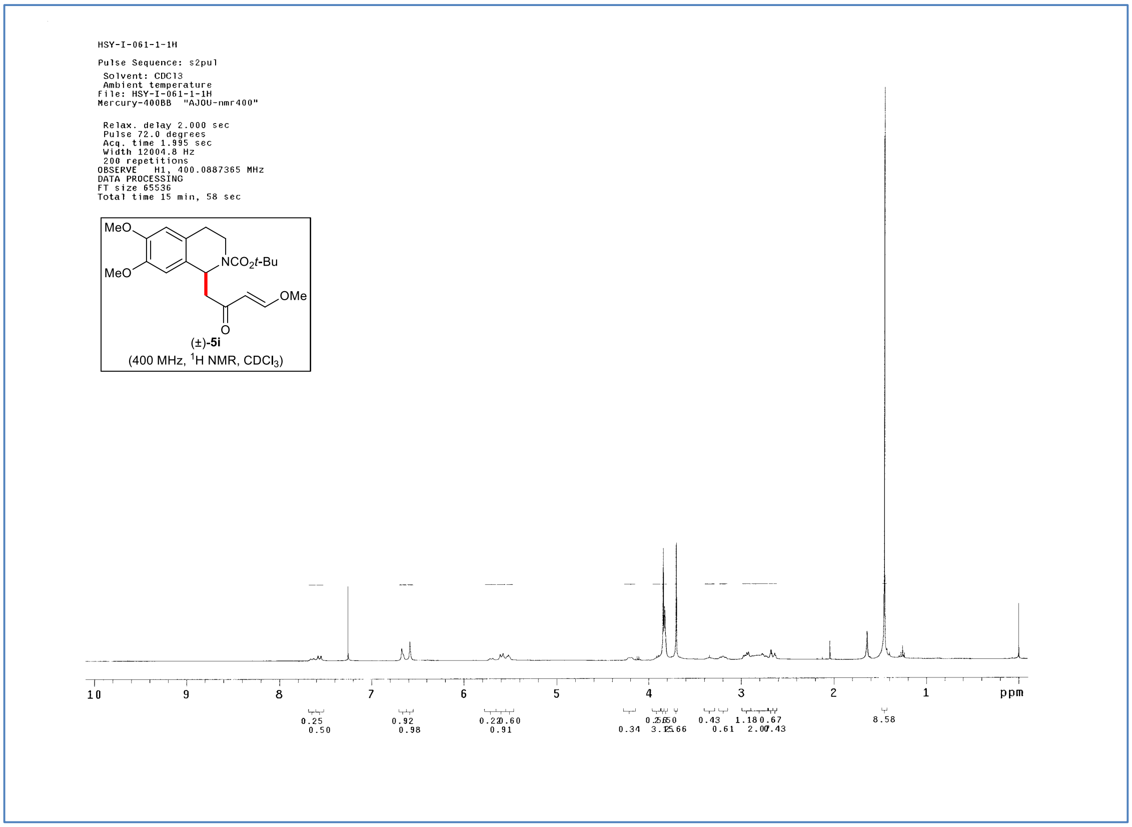

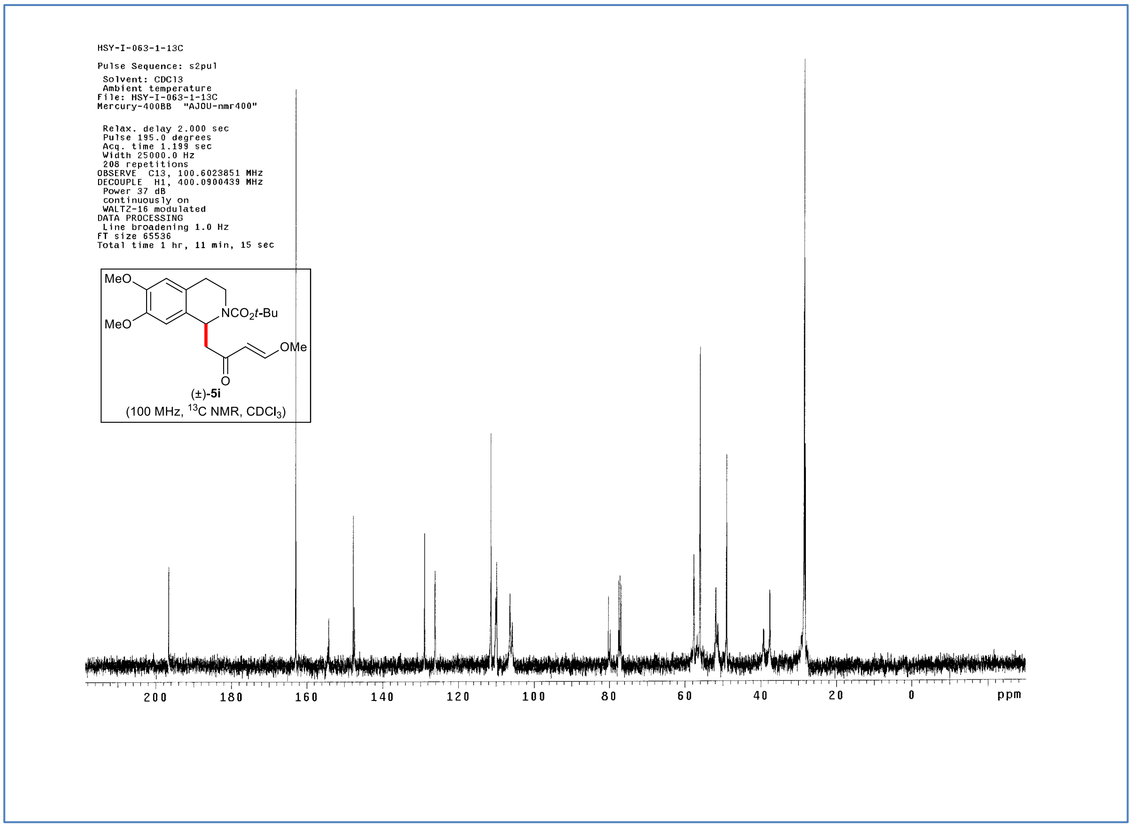

(±)-*tert*-Butyl 6,7-Dimethoxy-1-(2-methoxy-2-oxoethyl)-3,4-dihydroisoquinoline-2(1*H*)-carboxylate (**5j**)

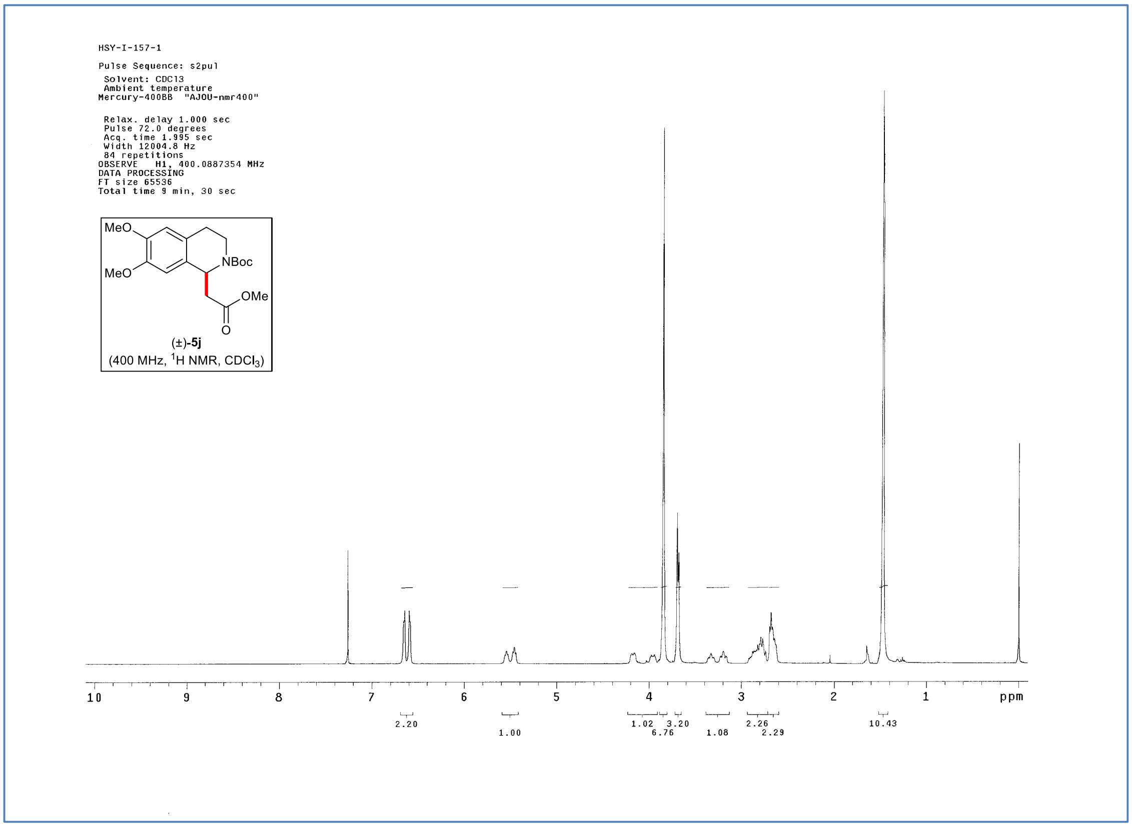

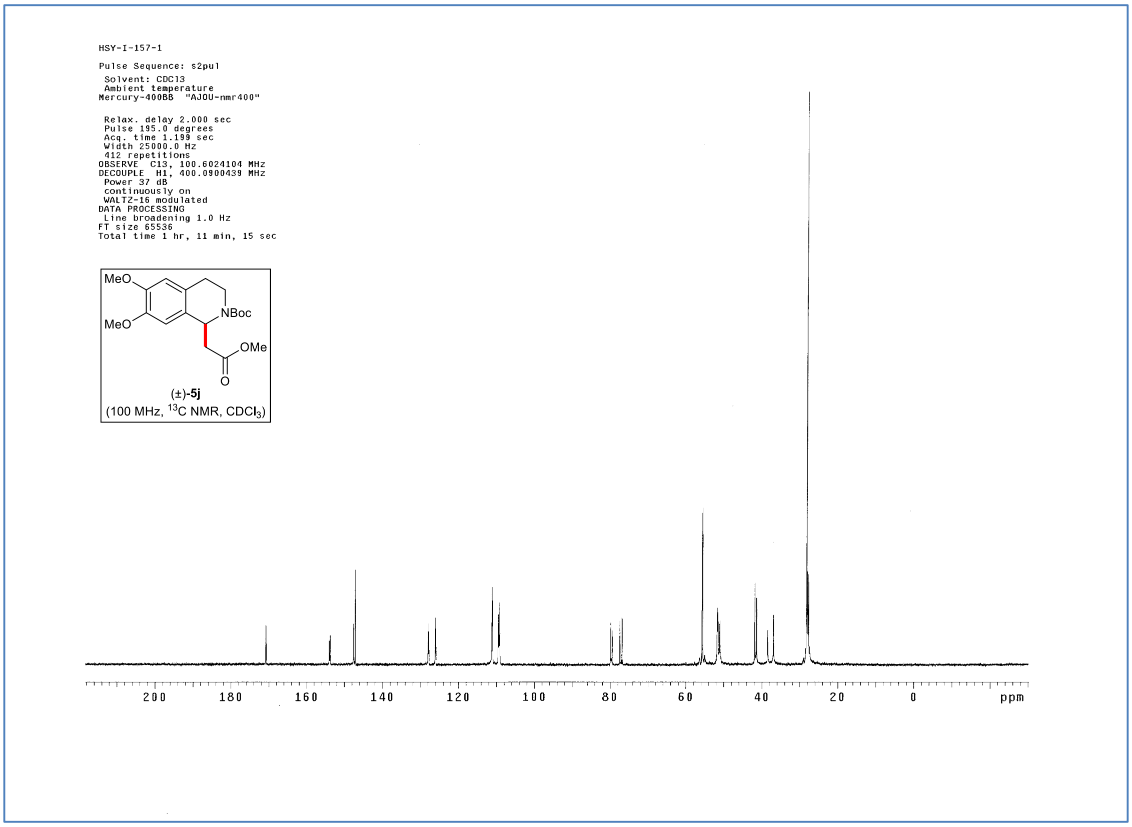

(±)-*tert*-Butyl 1-(2-Hydroxy-4,6-dimethoxyphenyl)-6,7-dimethoxy-3,4-dihydroisoquinoline-2(1*H*)-carboxylate (**5k**)

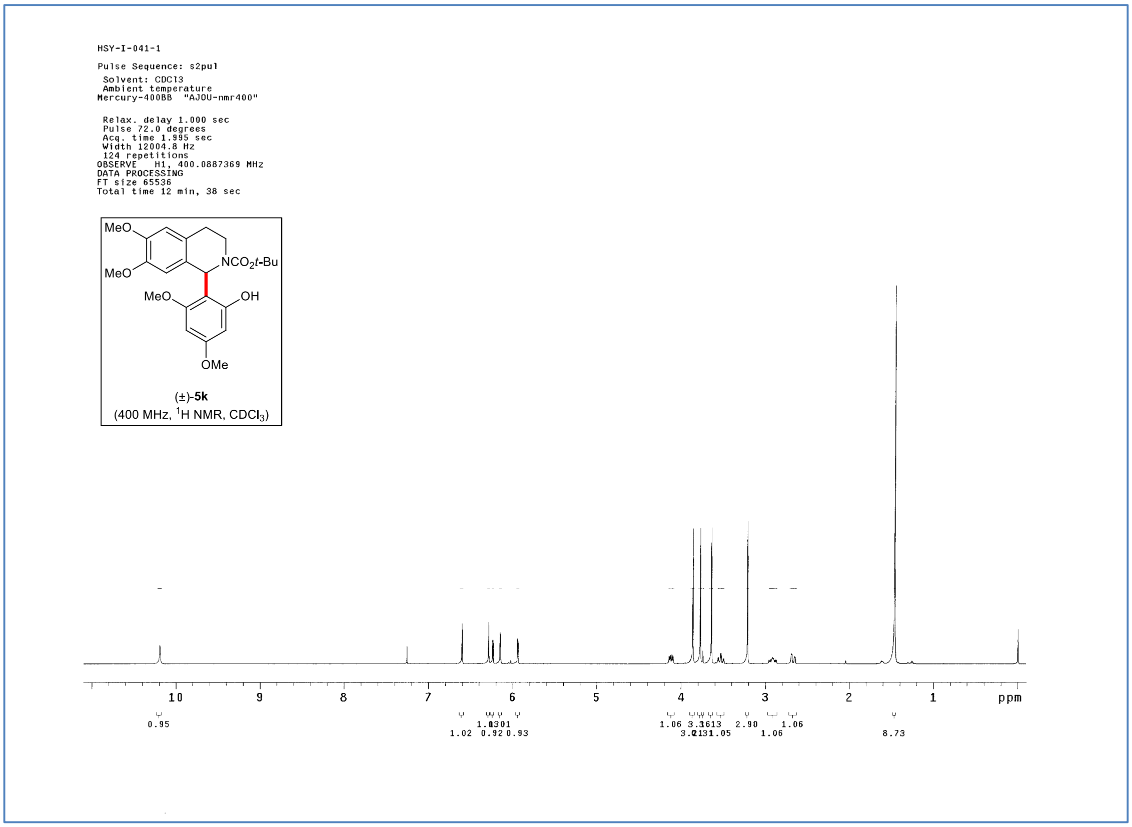

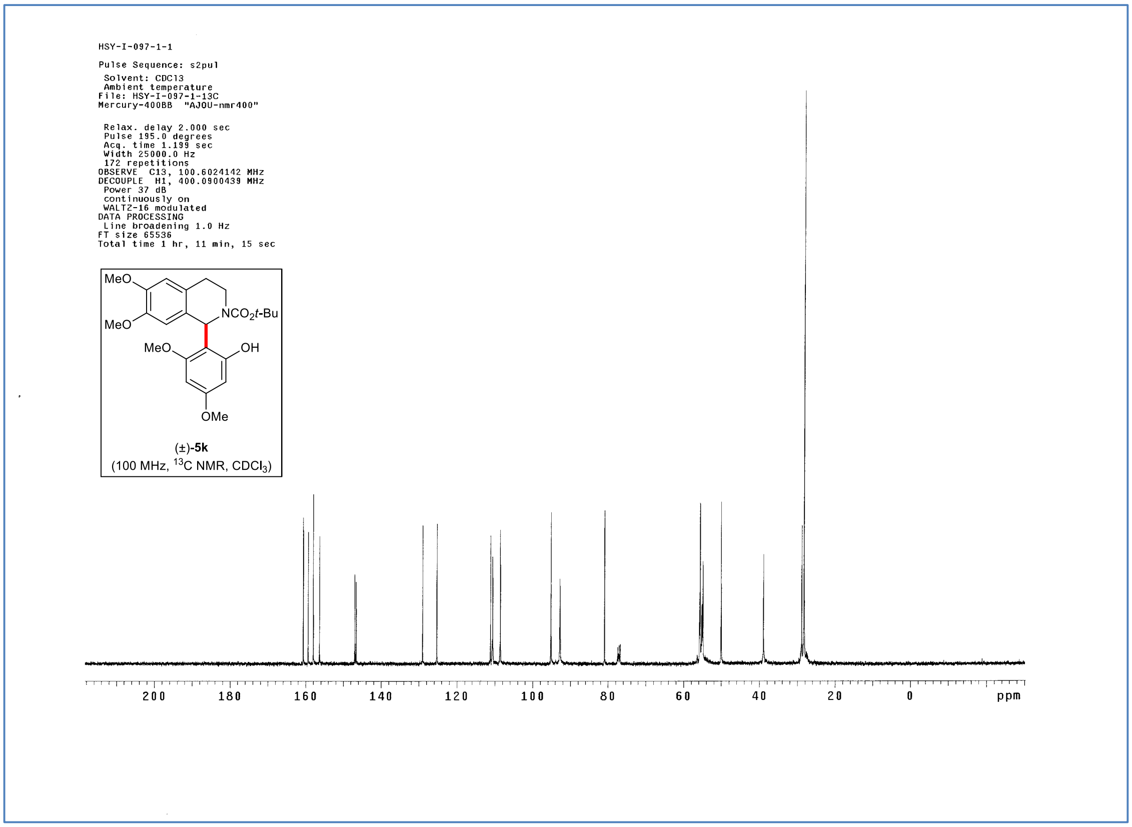

(±)-*tert*-Butyl 1-(4-(Dimethylamino)-2-hydroxyphenyl)-6,7-dimethoxy-3,4-dihydroisoquinoline-2(1*H*)-carboxylate (**5l**)

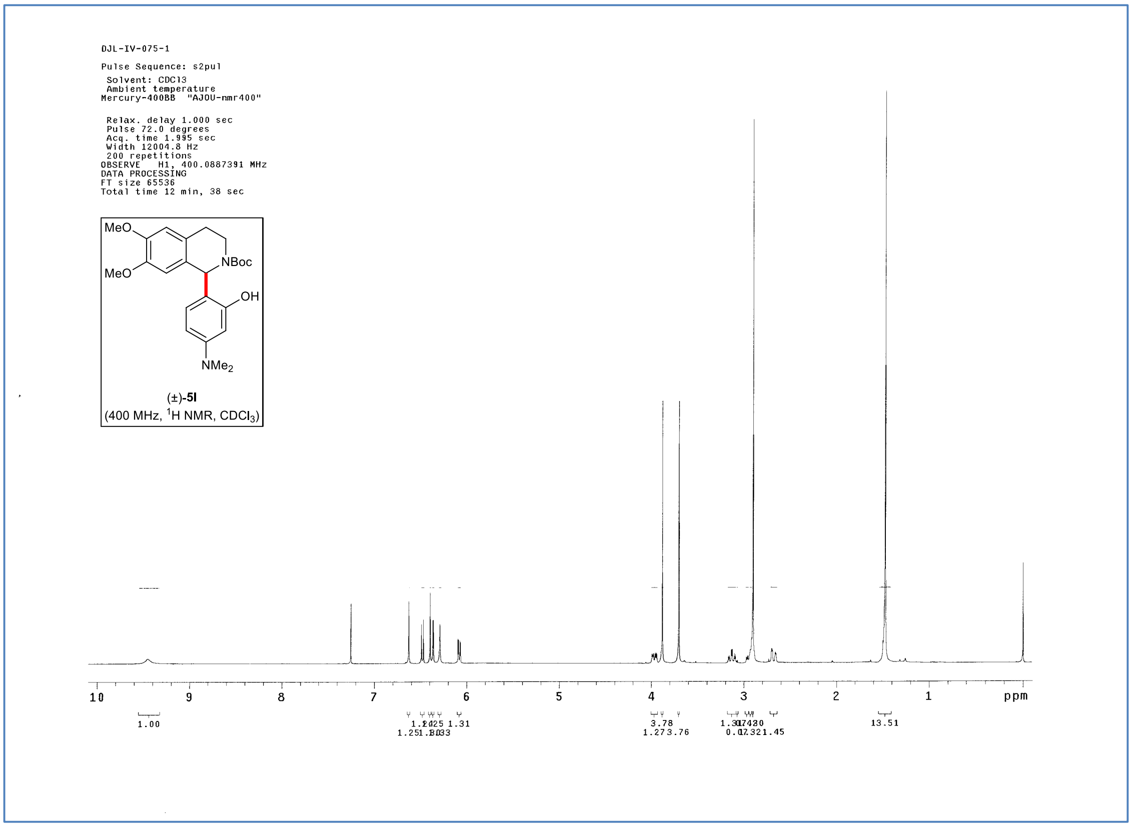

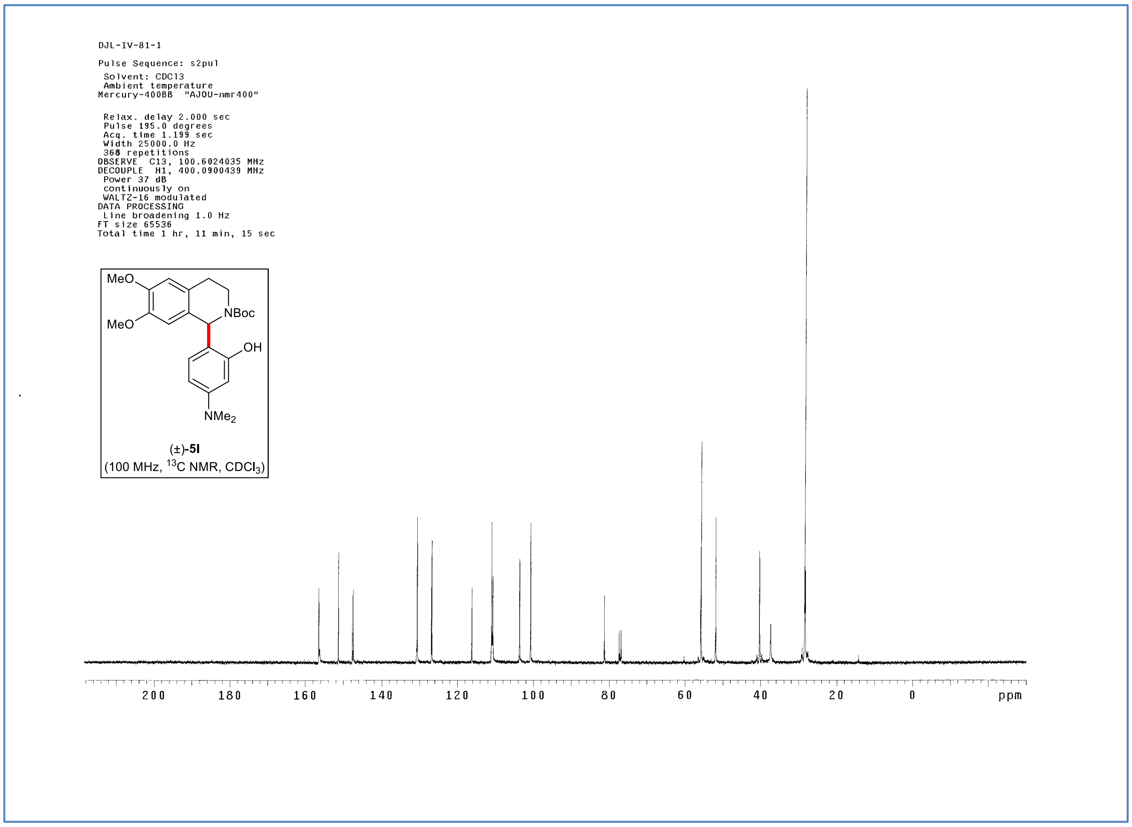

(±)-*tert*-Butyl 1-(4-(Diethylamino)phenyl)-6,7-dimethoxy-3,4-dihydroisoquinoline-2(1*H*)-carboxylate (**5m**)

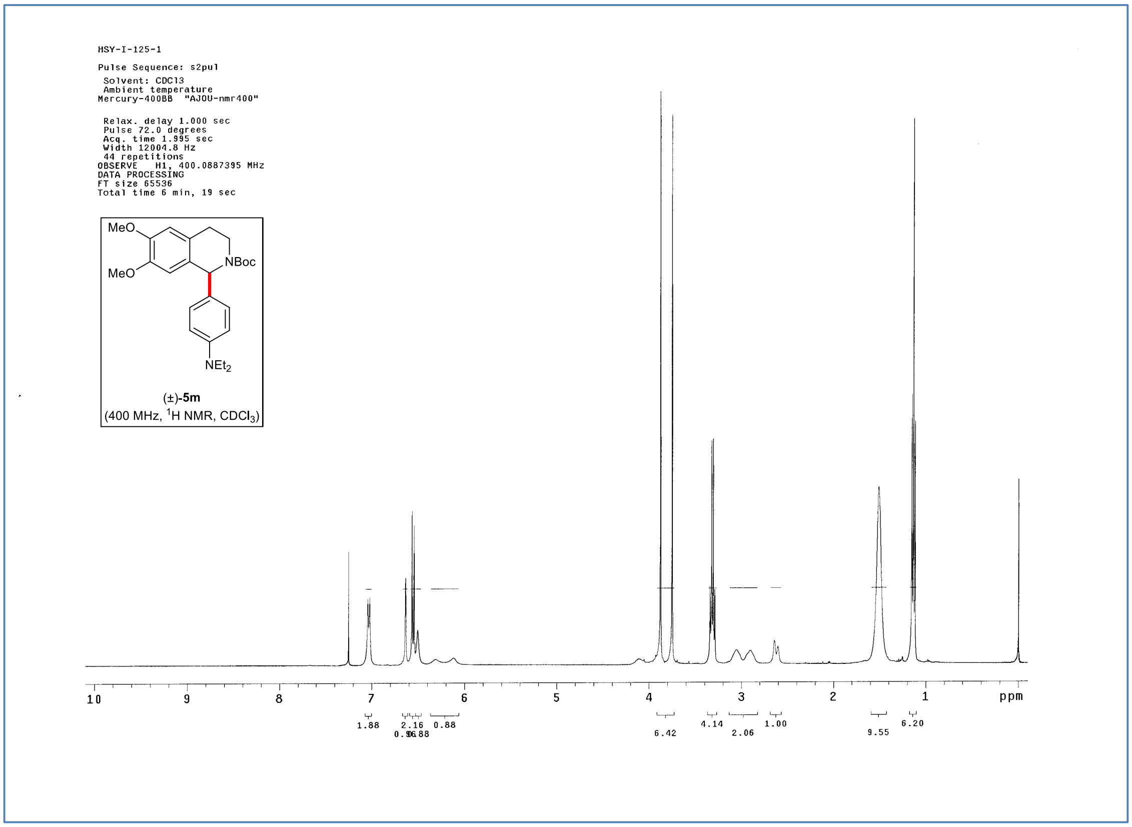

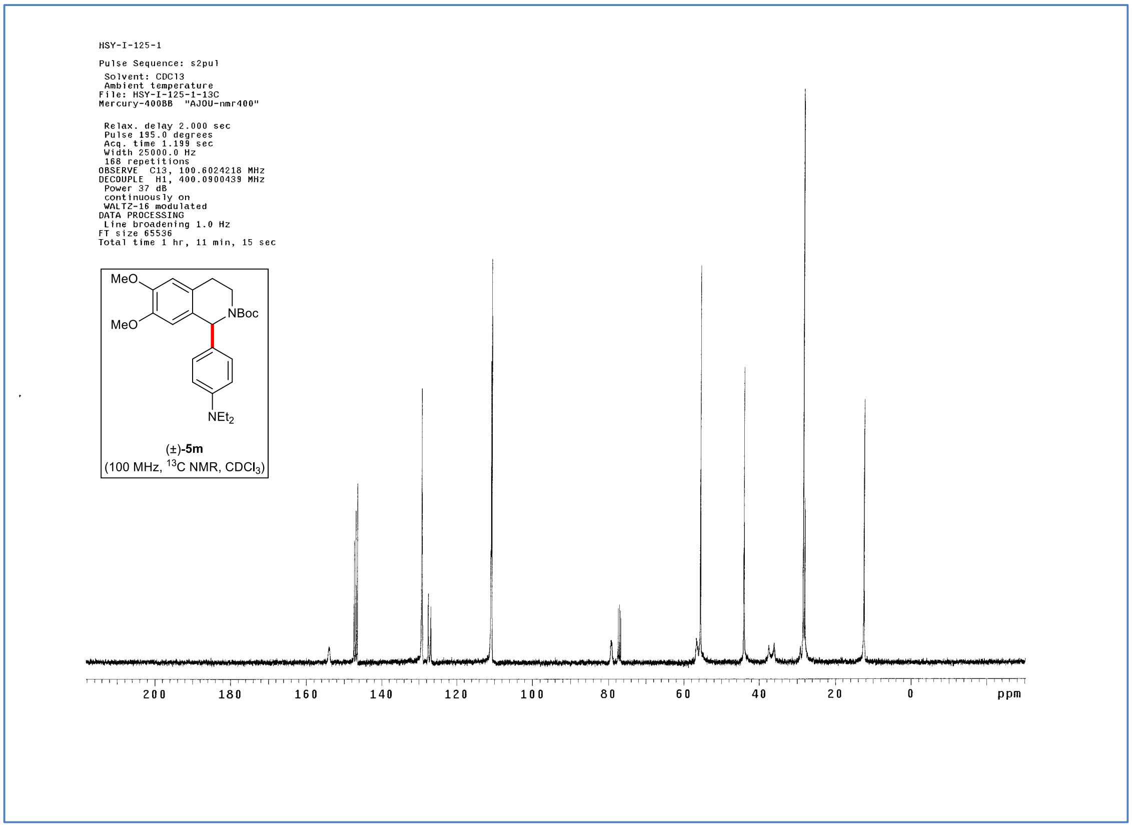

(±)-*tert*-Butyl 1-(1-Hydroxynaphthalen-2-yl)-6,7-dimethoxy-3,4-dihydroisoquinoline-2(1*H*)-carboxylate (**5n**)

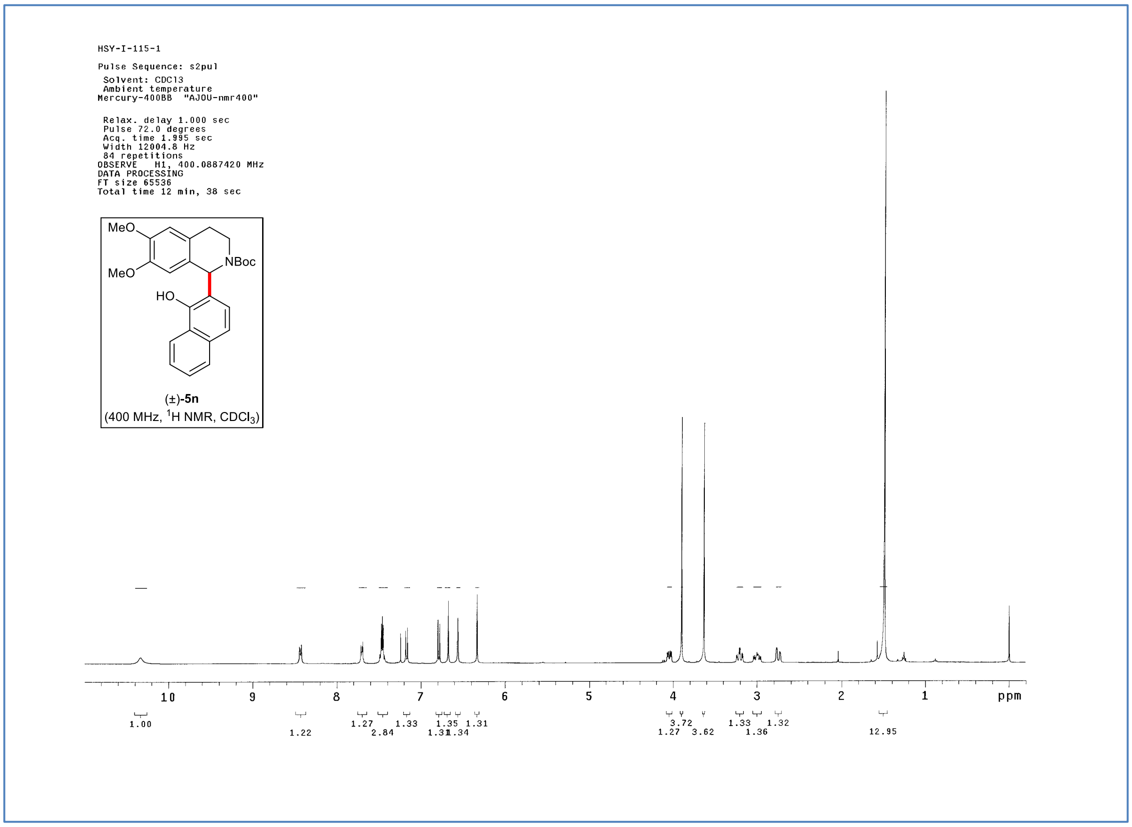

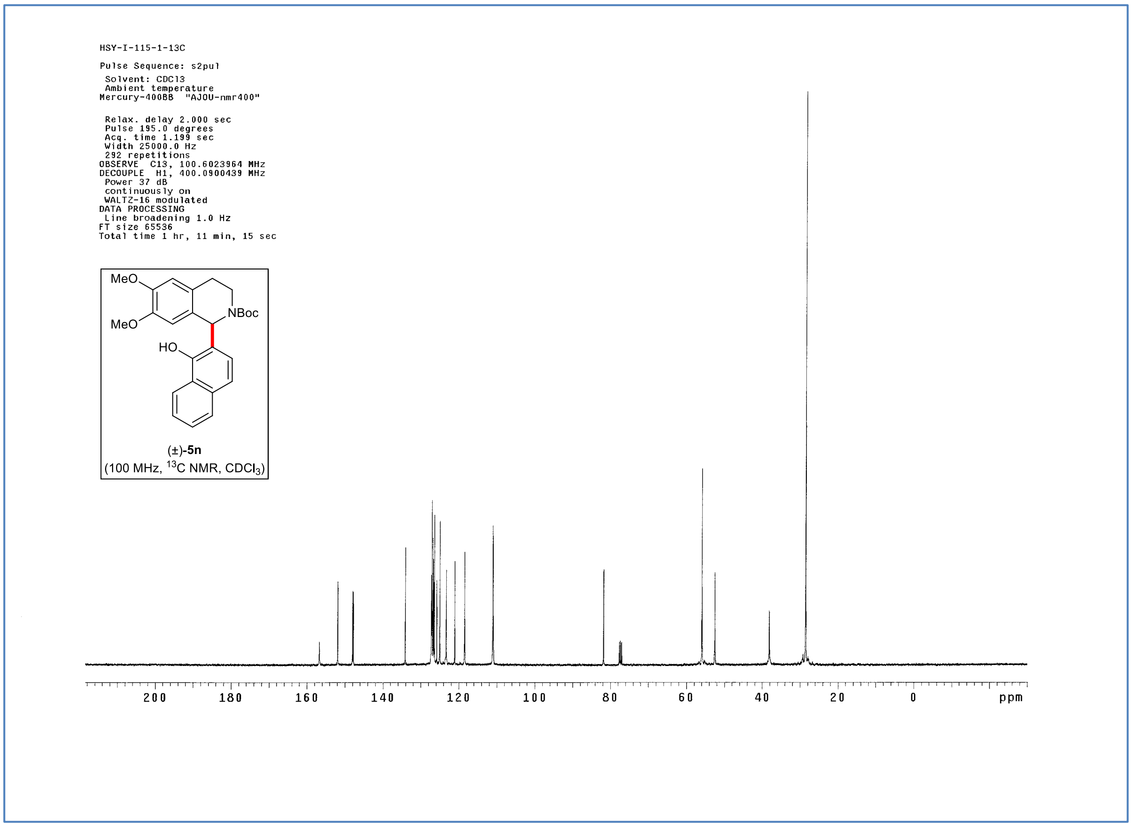

(±)-*tert*-Butyl 1-(1*H*-Indol-3-yl)-6,7-dimethoxy-3,4-dihydroisoquinoline-2(1*H*)-carboxylate (**5o**)

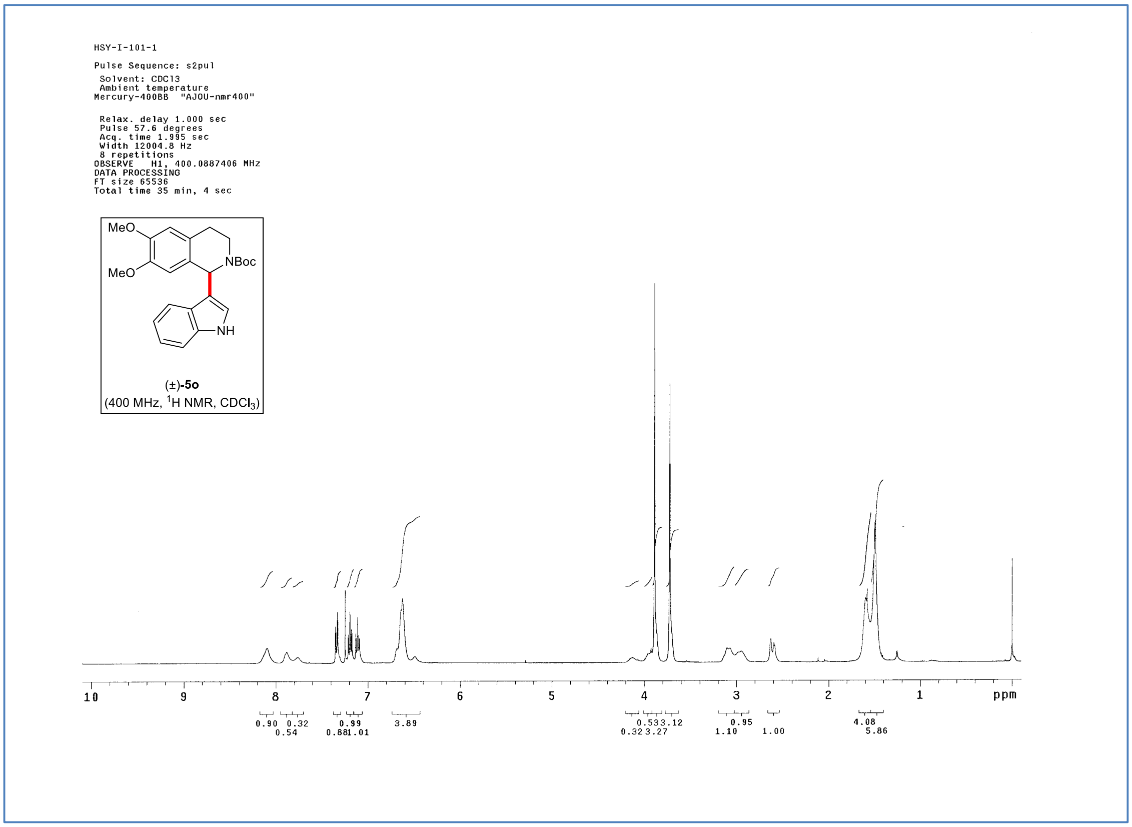

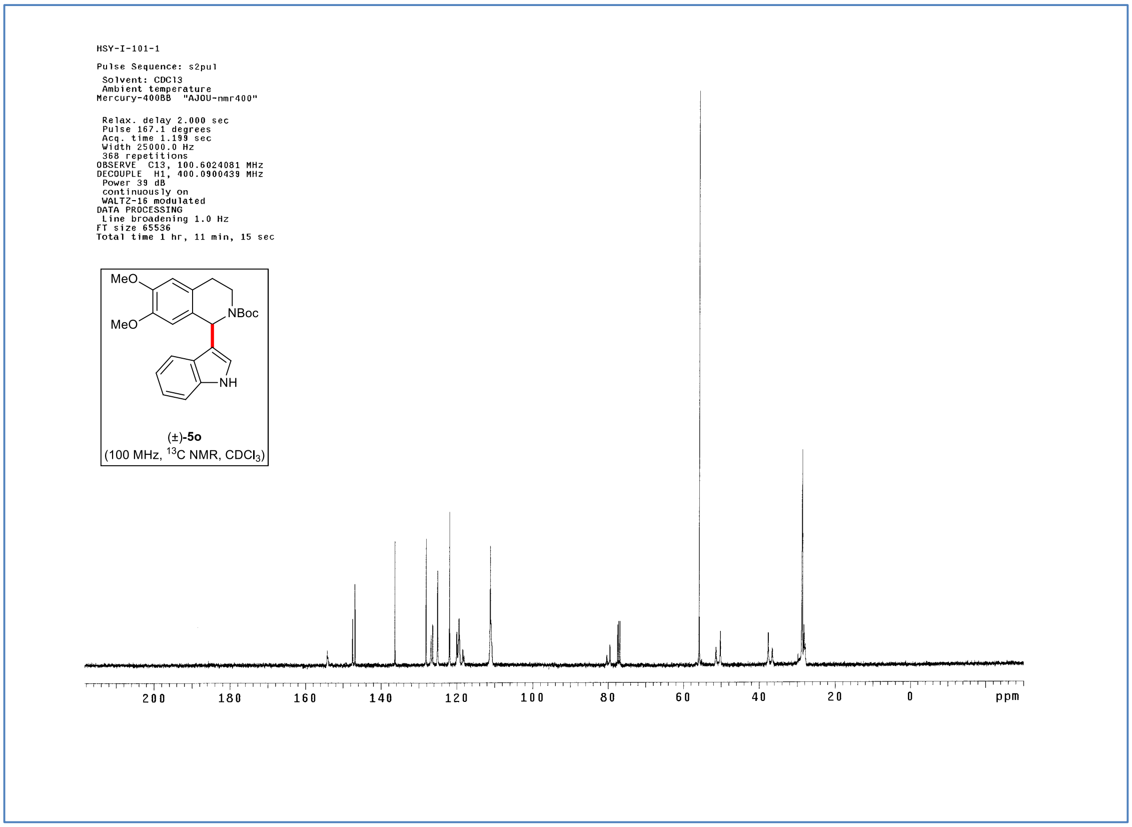

(±)-*tert*-Butyl 6,7-Dimethoxy-1-(5-methylfuran-2-yl)-3,4-dihydroisoquinoline-2(1*H*)-carboxylate (**5p**)

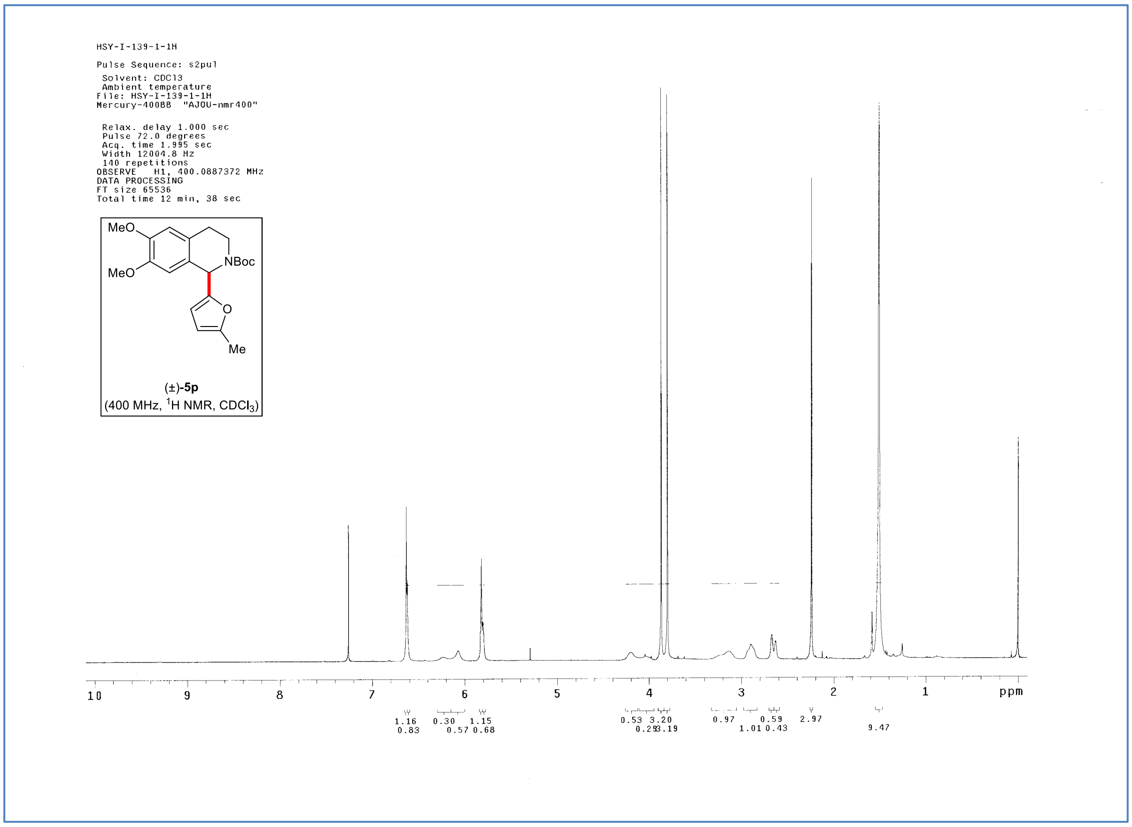

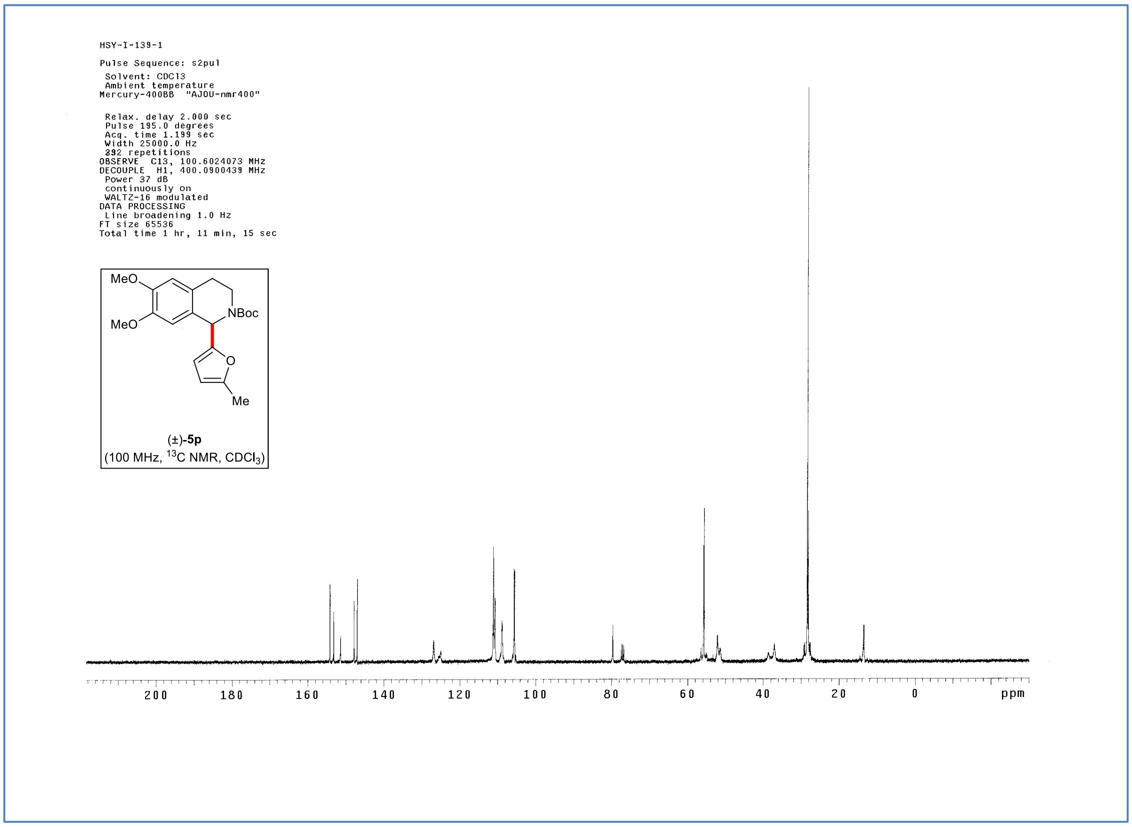

(±)-Benzyl 1-Allyl-6,7-dimethoxy-3,4-dihydroisoquinoline-2(1*H*)-carboxylate (**6a**)

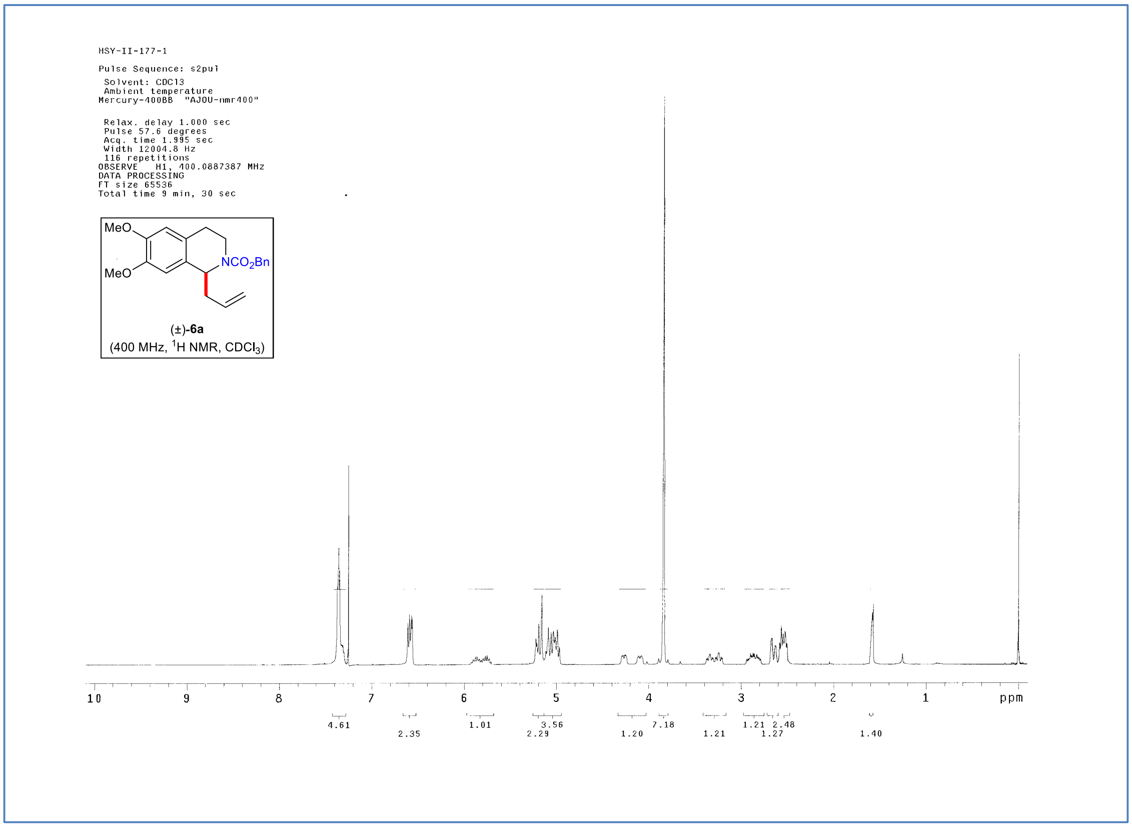

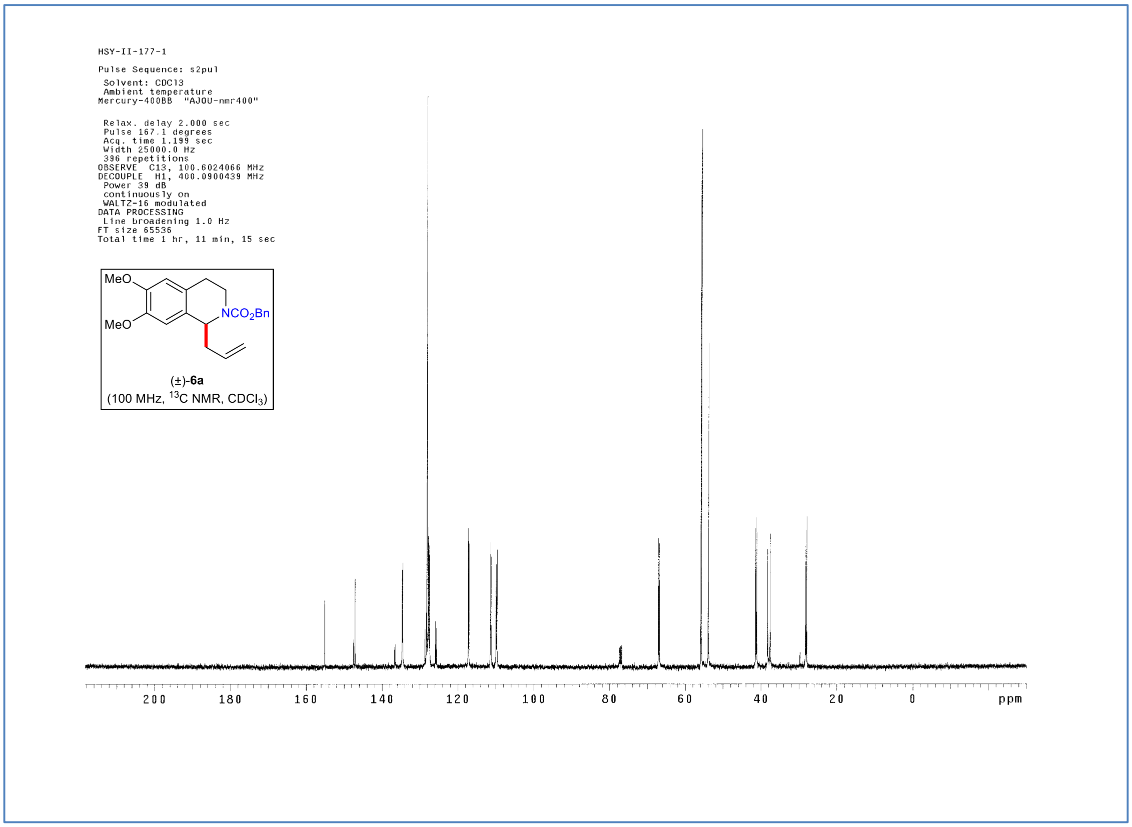

(±)-Allyl 1-Allyl-6,7-dimethoxy-3,4-dihydroisoquinoline-2(1*H*)-carboxylate (**6b**)

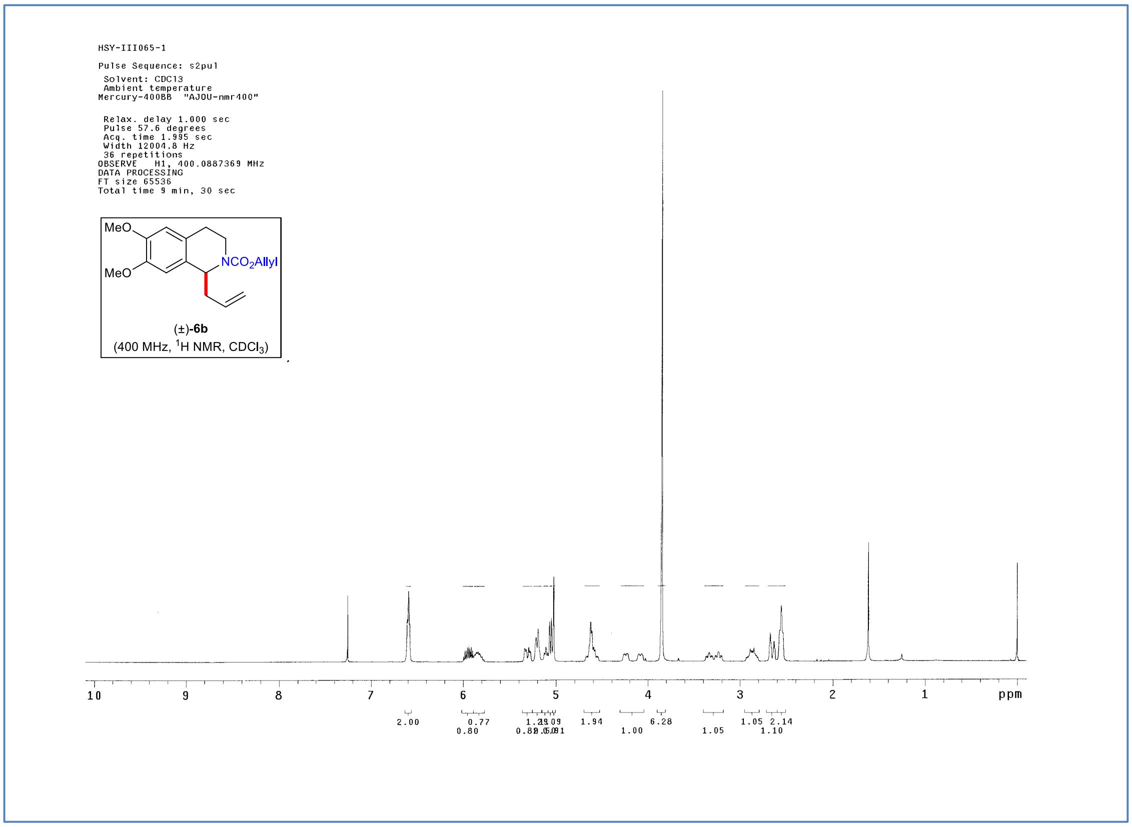

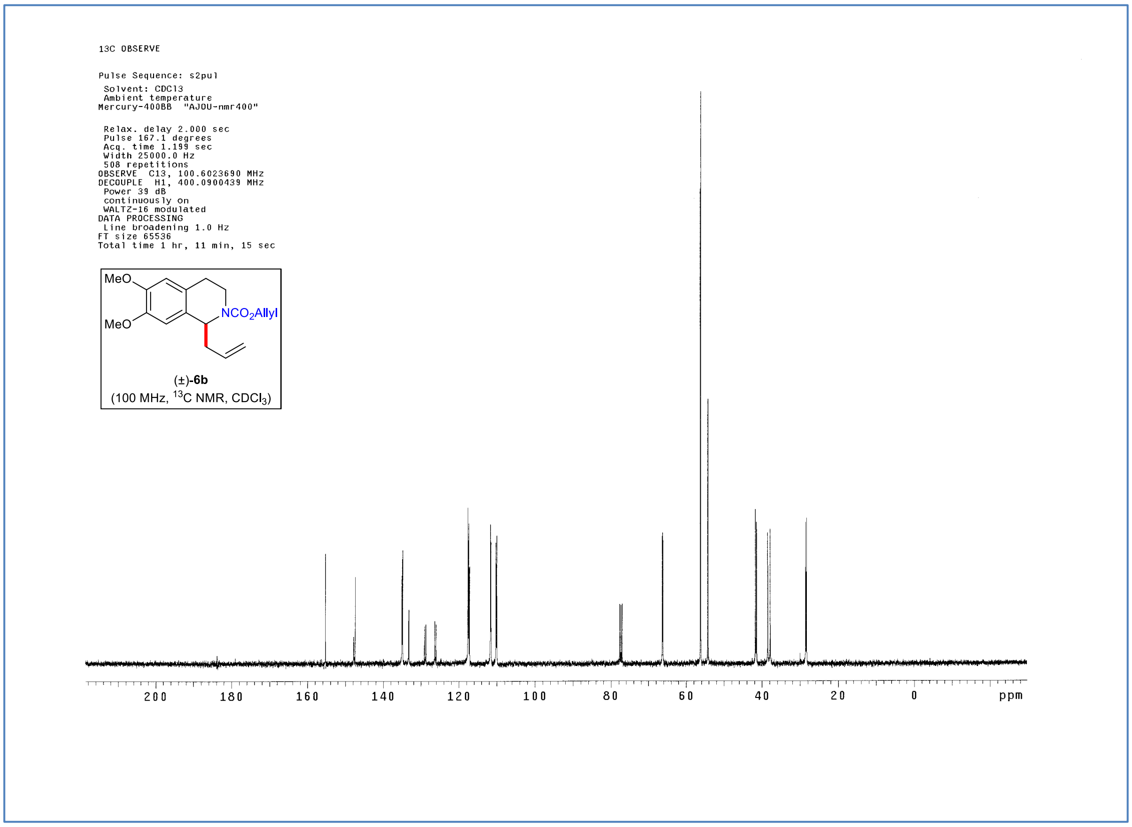

(±)-Methyl 1-Allyl-6,7-dimethoxy-3,4-dihydroisoquinoline-2(1*H*)-carboxylate (**6c**)

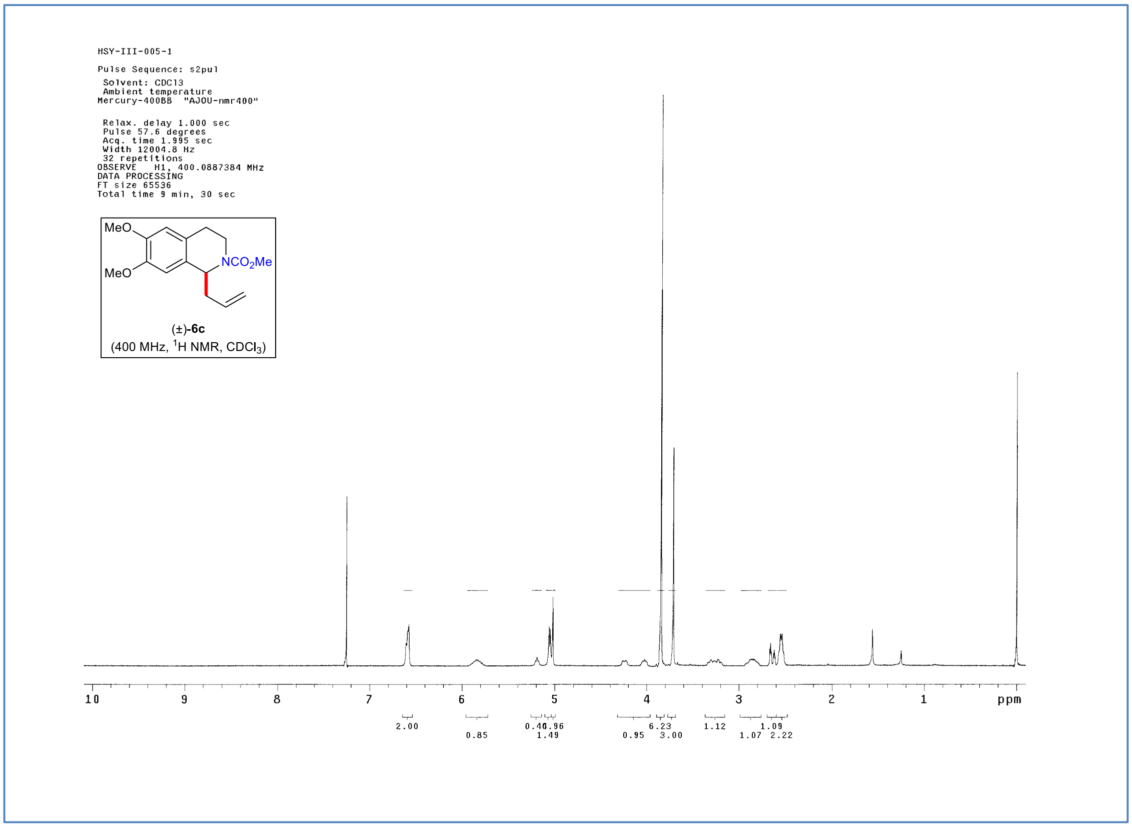

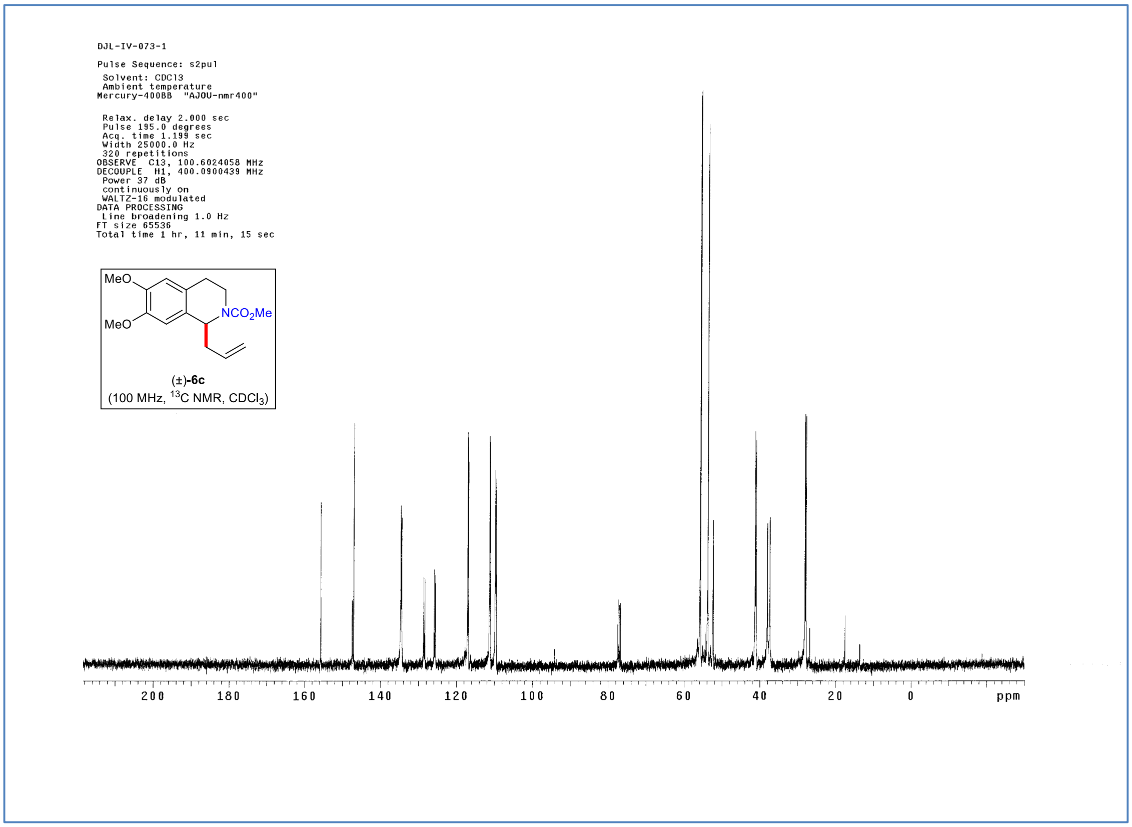

(±)-Ethyl 1-Allyl-6,7-dimethoxy-3,4-dihydroisoquinoline-2(1*H*)-carboxylate (**6d**)

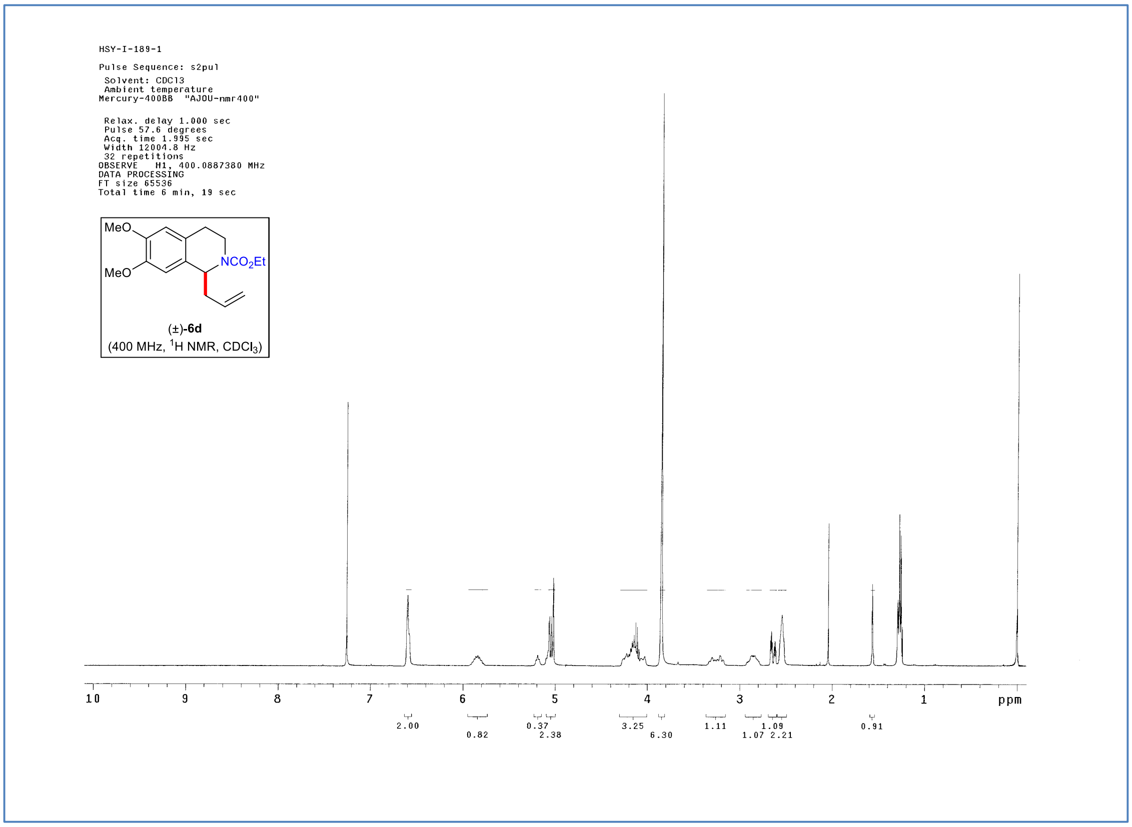

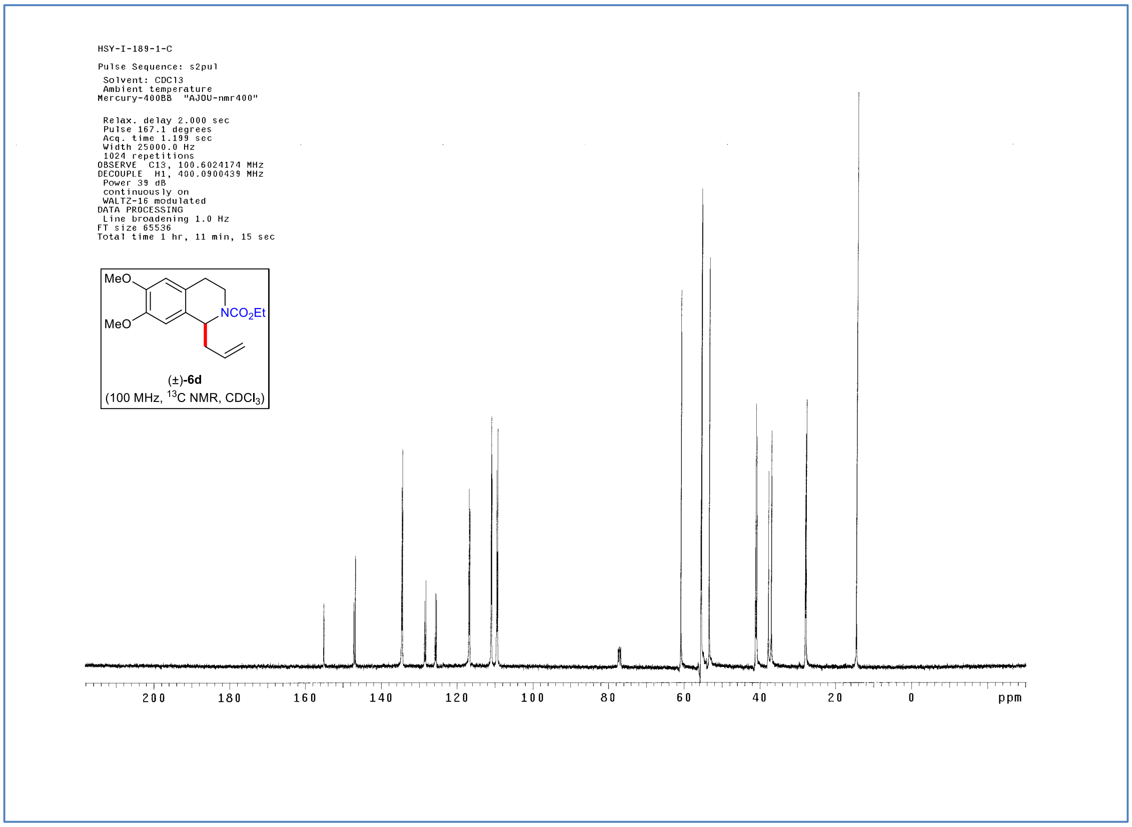

(±)-1-(1-Allyl-6,7-dimethoxy-3,4-dihydroisoquinolin-2(1*H*)-yl)ethanone (**6e**)

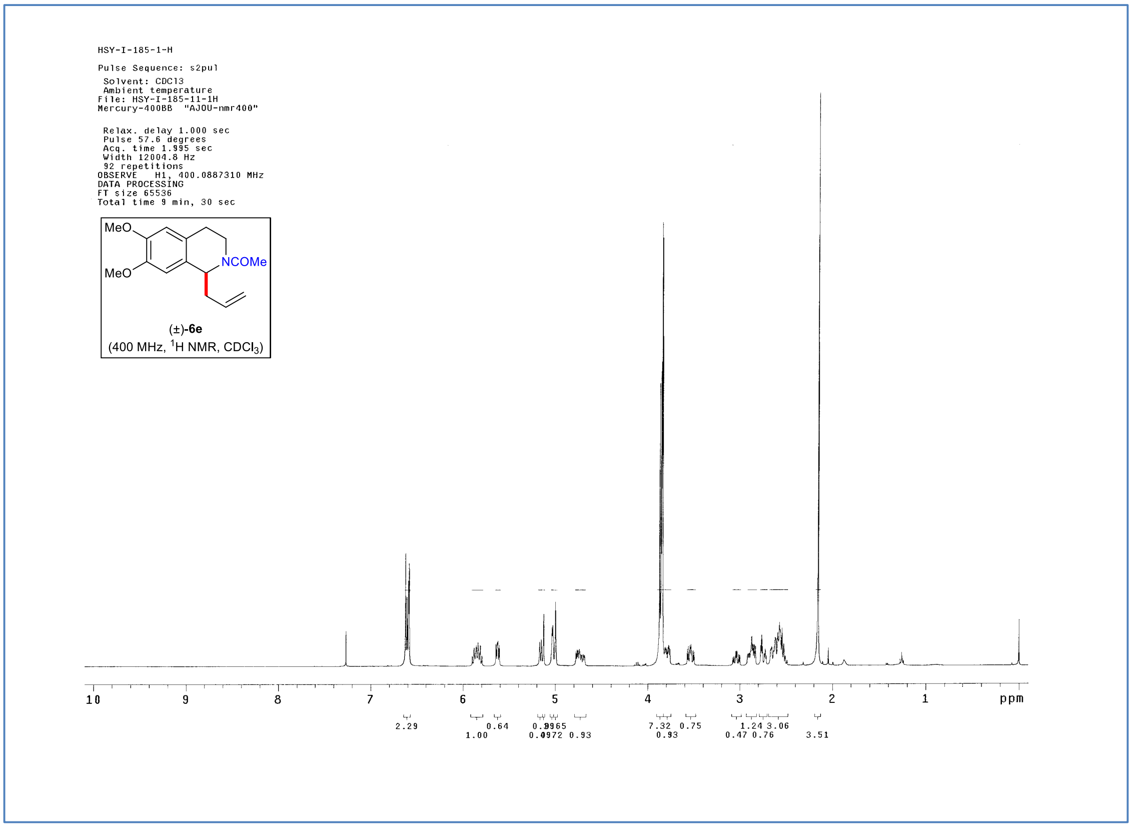

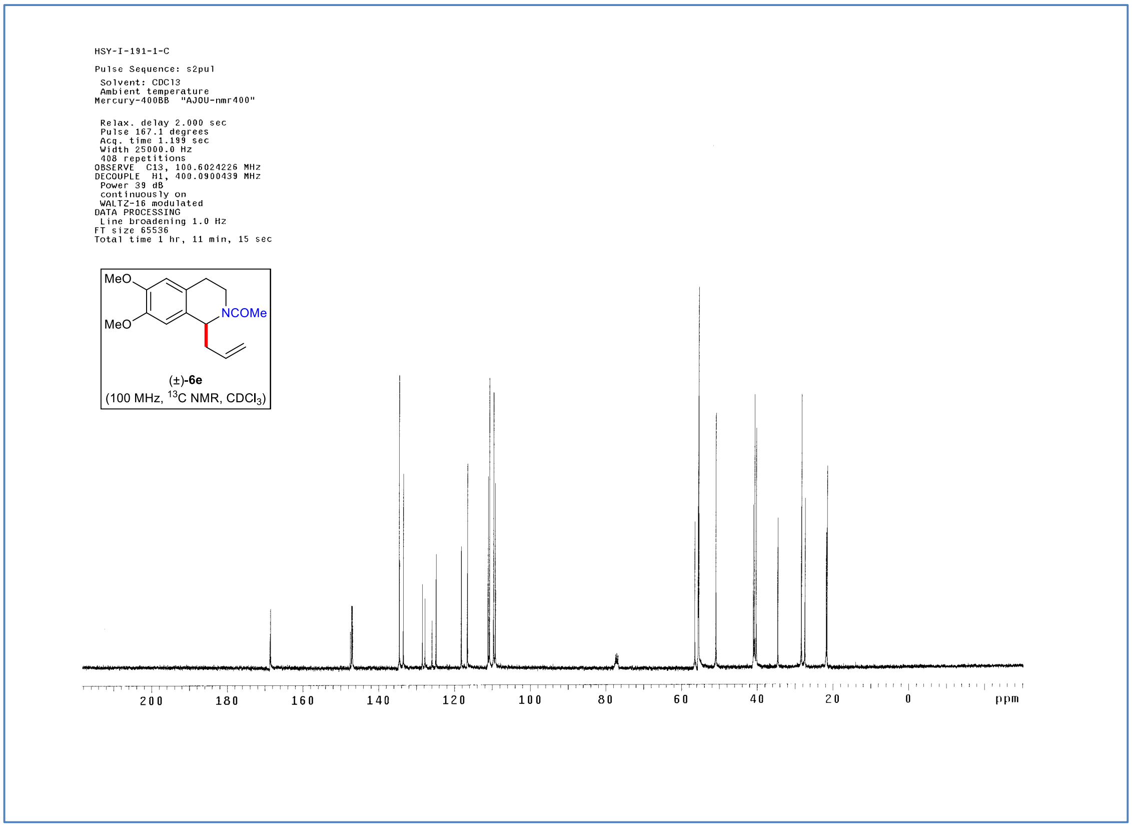

(±)-(1-Allyl-6,7-dimethoxy-3,4-dihydroisoquinolin-2(1*H*)-yl)(phenyl)methanone (**6f**)

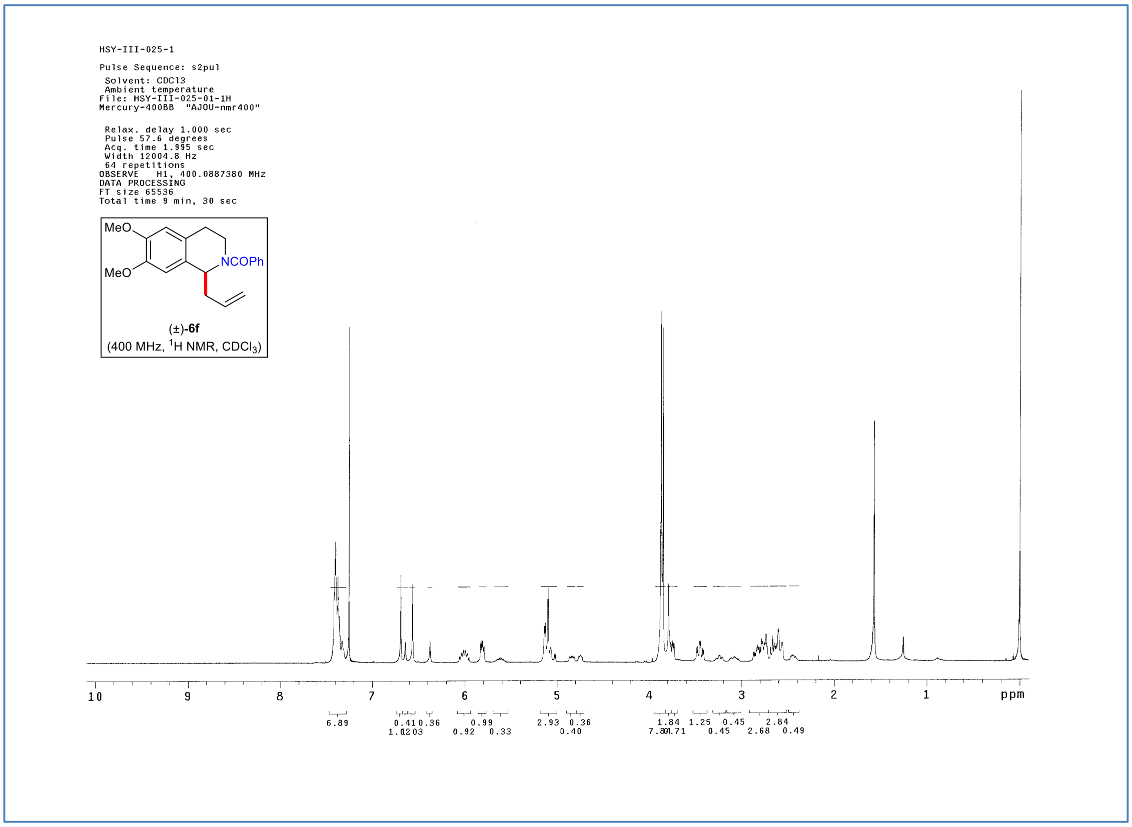

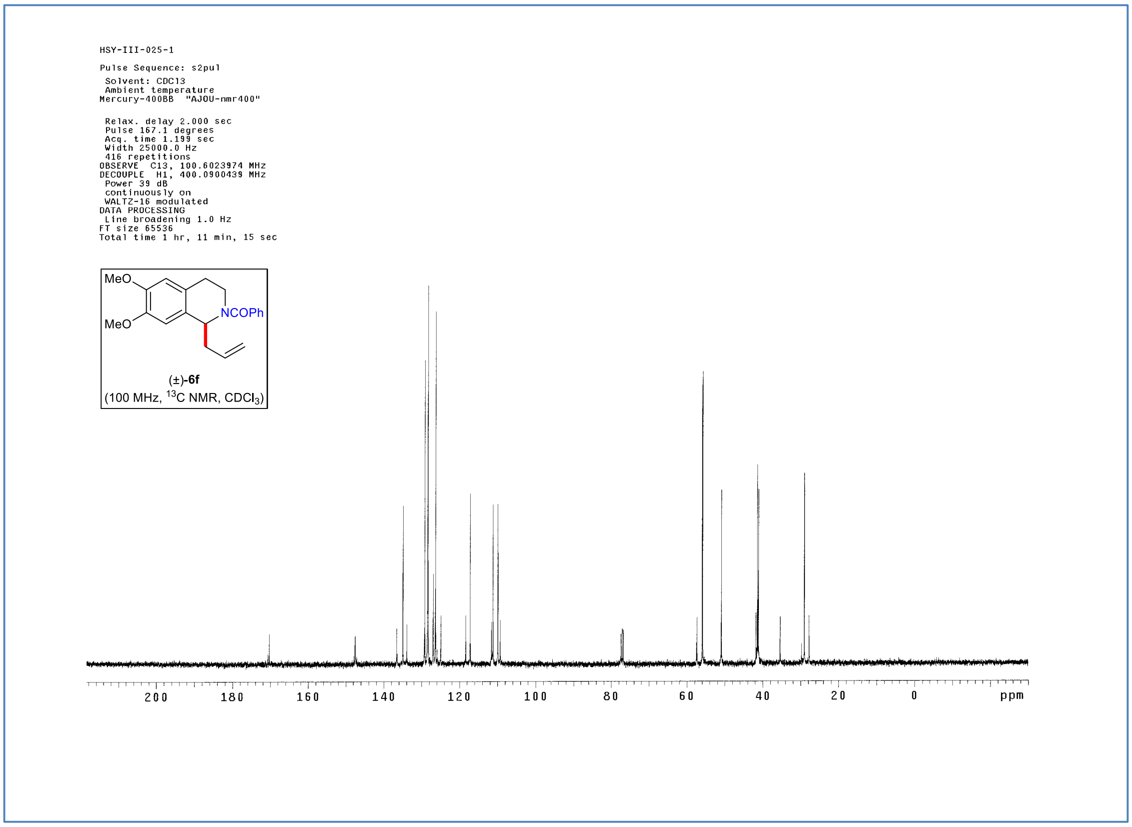

(±)-1-Allyl-6,7-dimethoxy-2-(methylsulfonyl)-1,2,3,4-tetrahydroisoquinoline (**6g**)

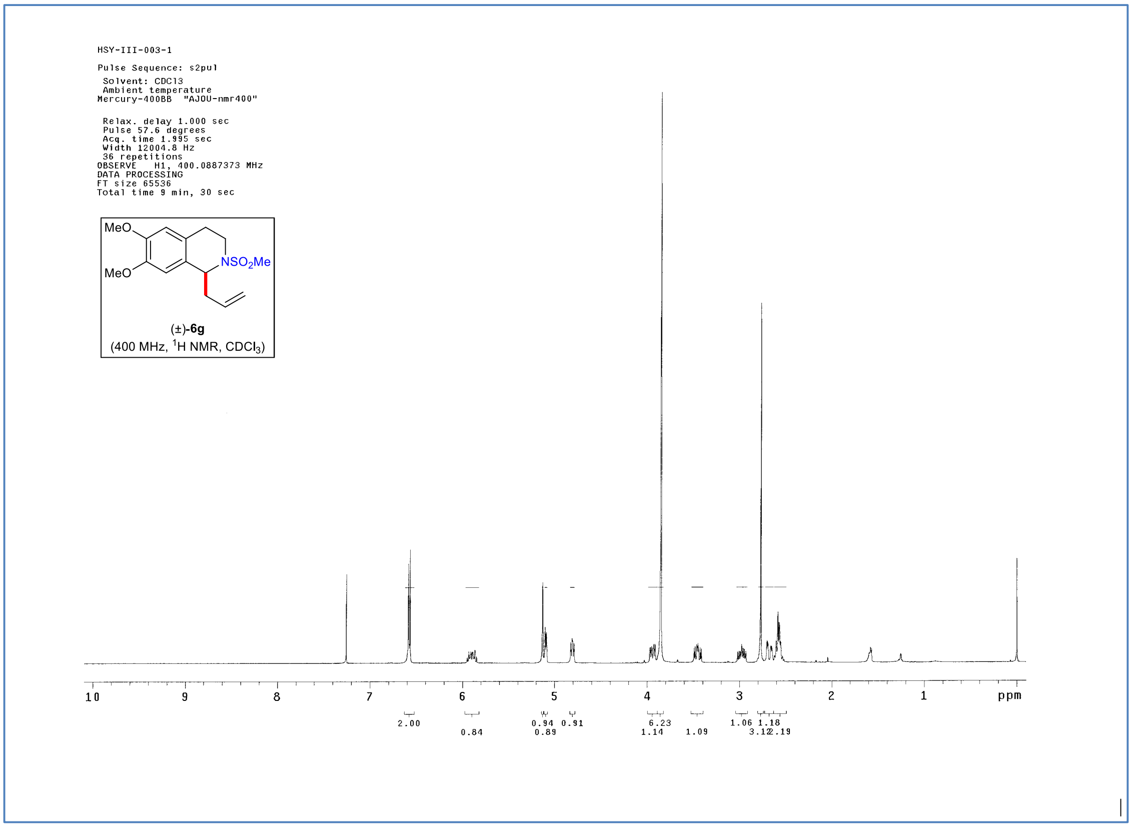

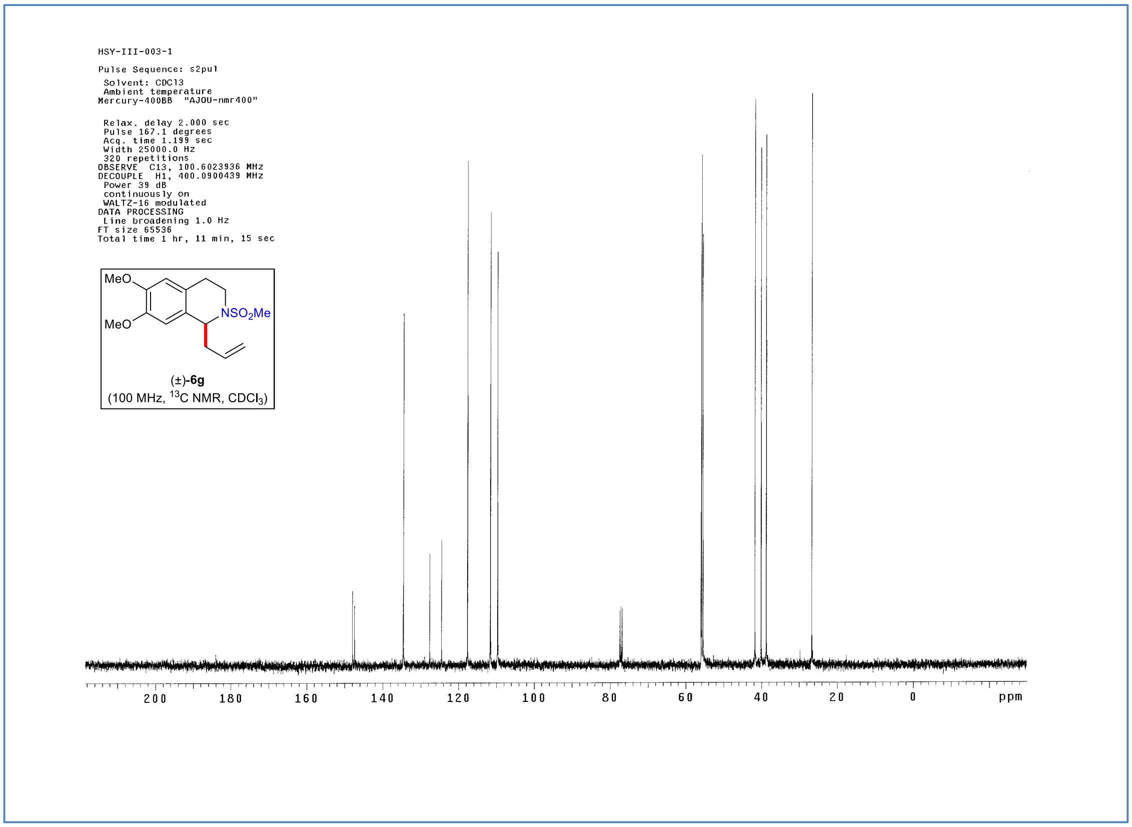

(±)-1-Allyl-6,7-dimethoxy-2-tosyl-1,2,3,4-tetrahydroisoquinoline (**6h**)

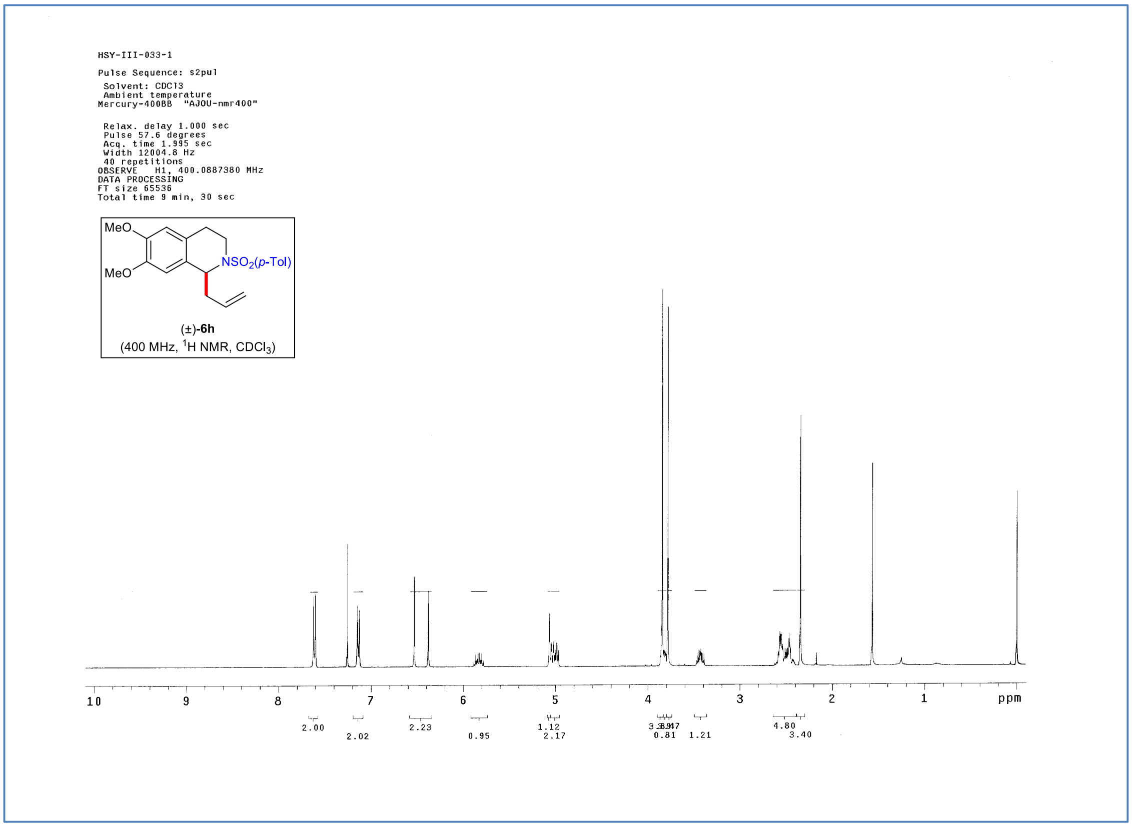

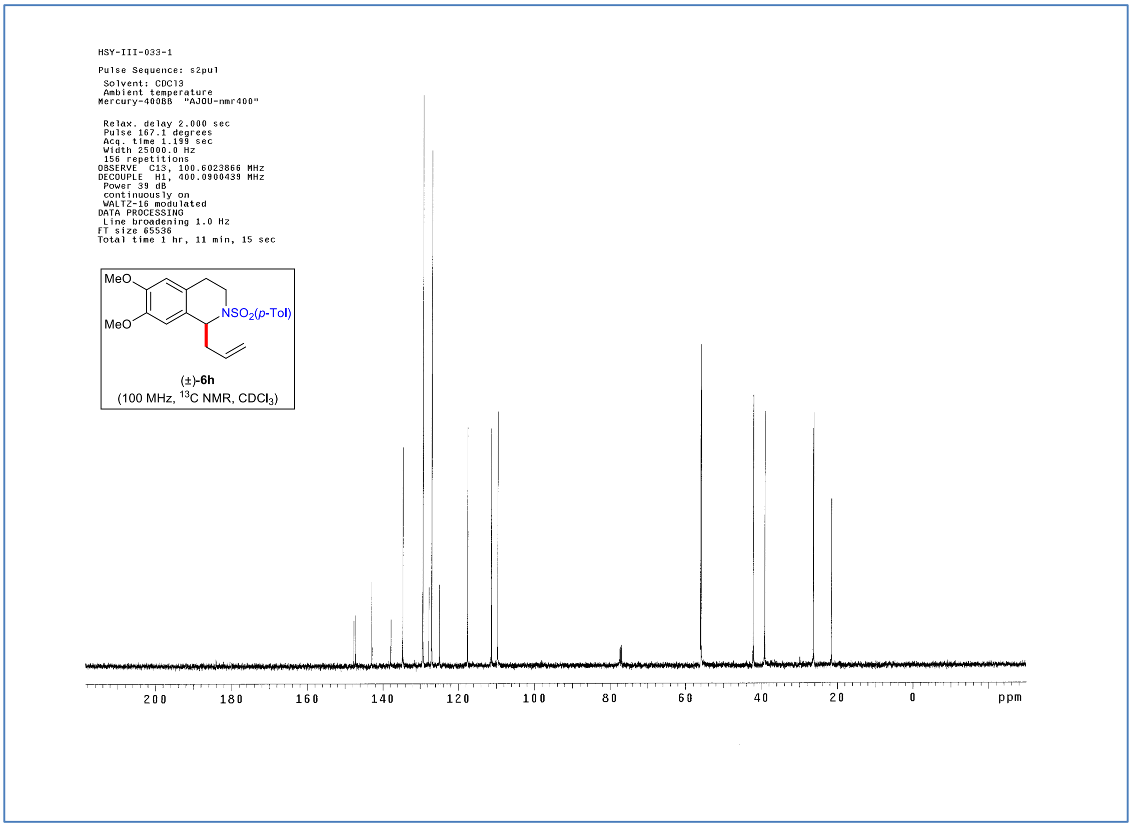

(±)-1-Allyl-6,7-dimethoxy-2-((2-nitrophenyl)sulfonyl)-1,2,3,4-tetrahydroisoquinoline (**6i**)

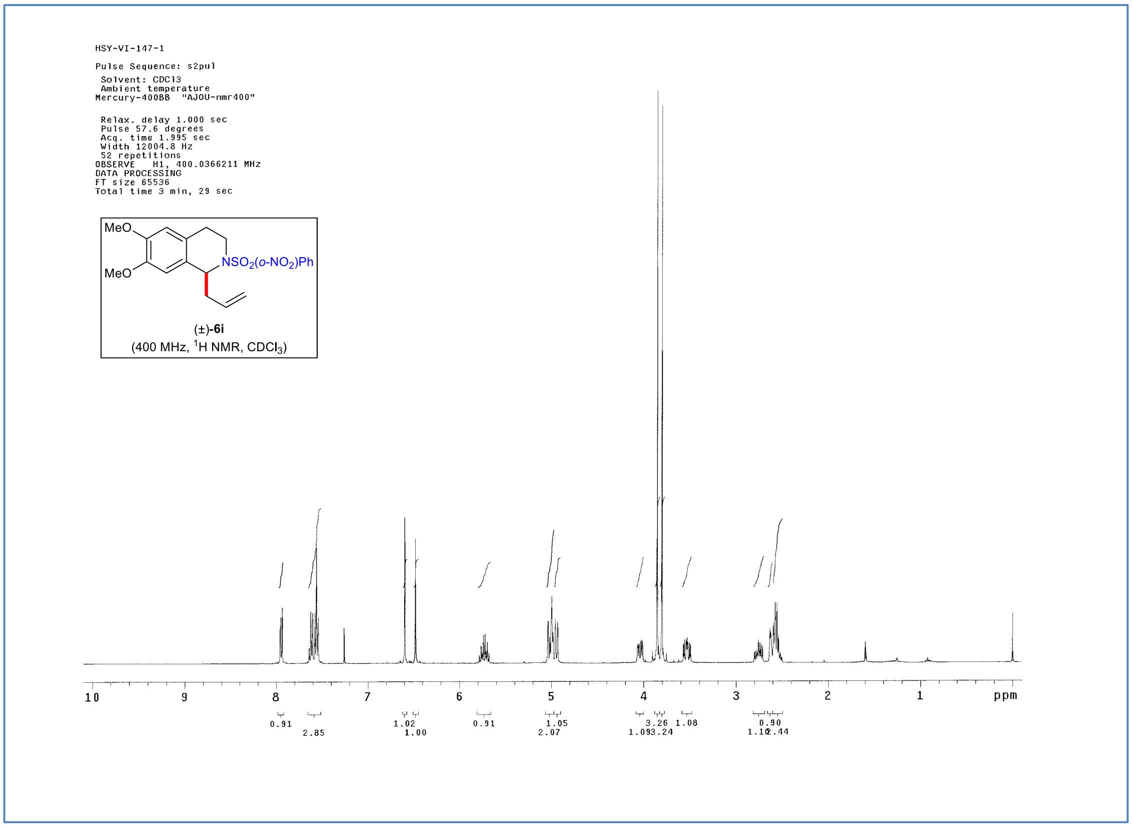

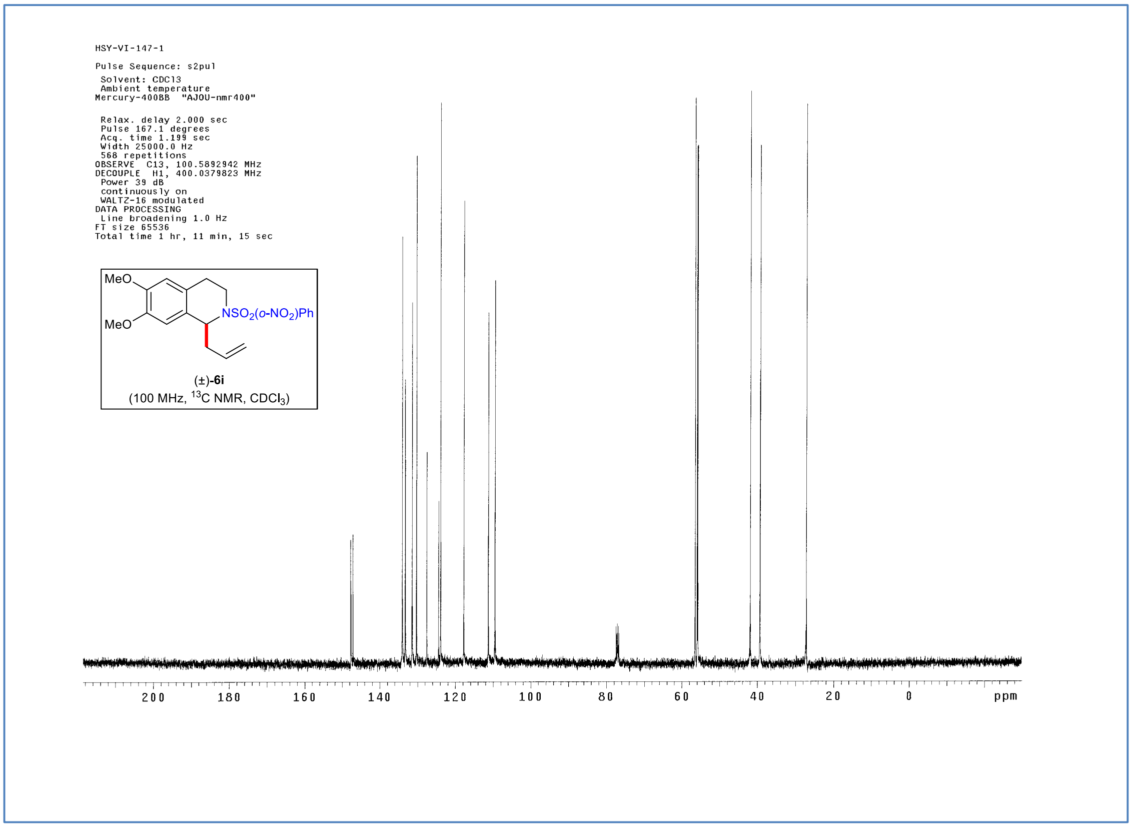

(±)-*tert*-Butyl 1-Allyl-6-methoxy-3,4-dihydroisoquinoline-2(1*H*)-carboxylate (**7a**)

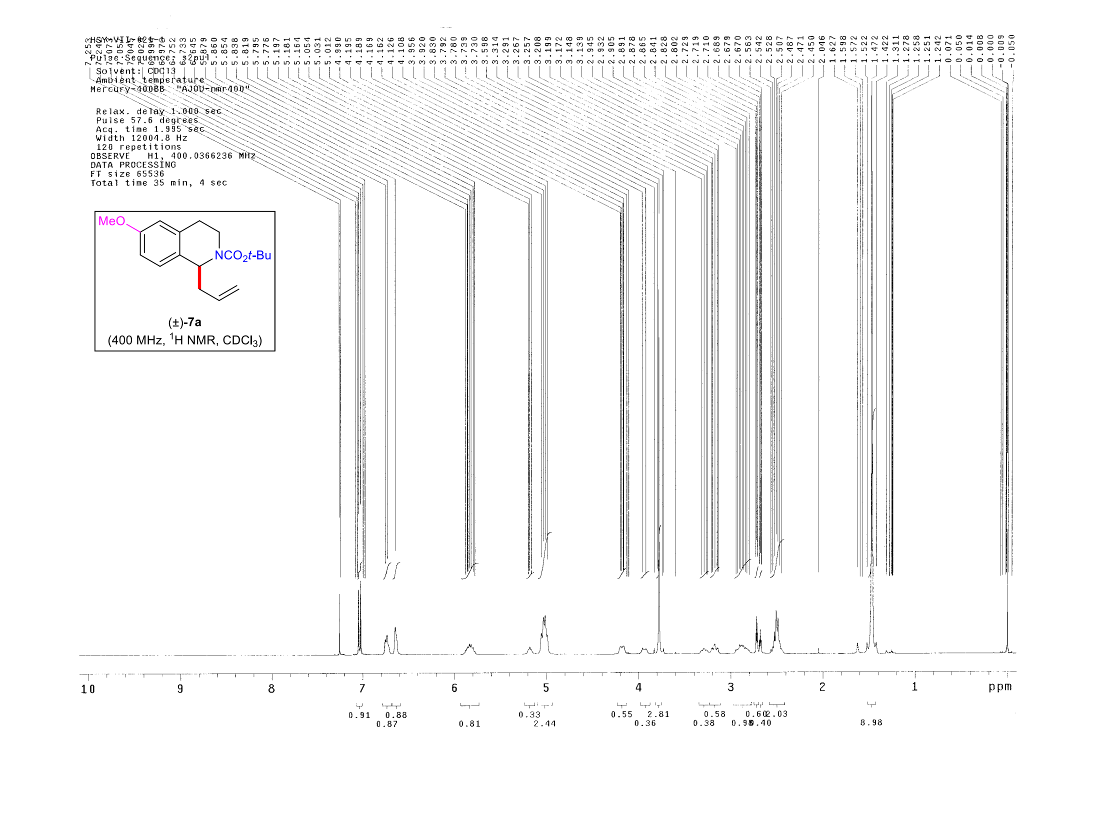

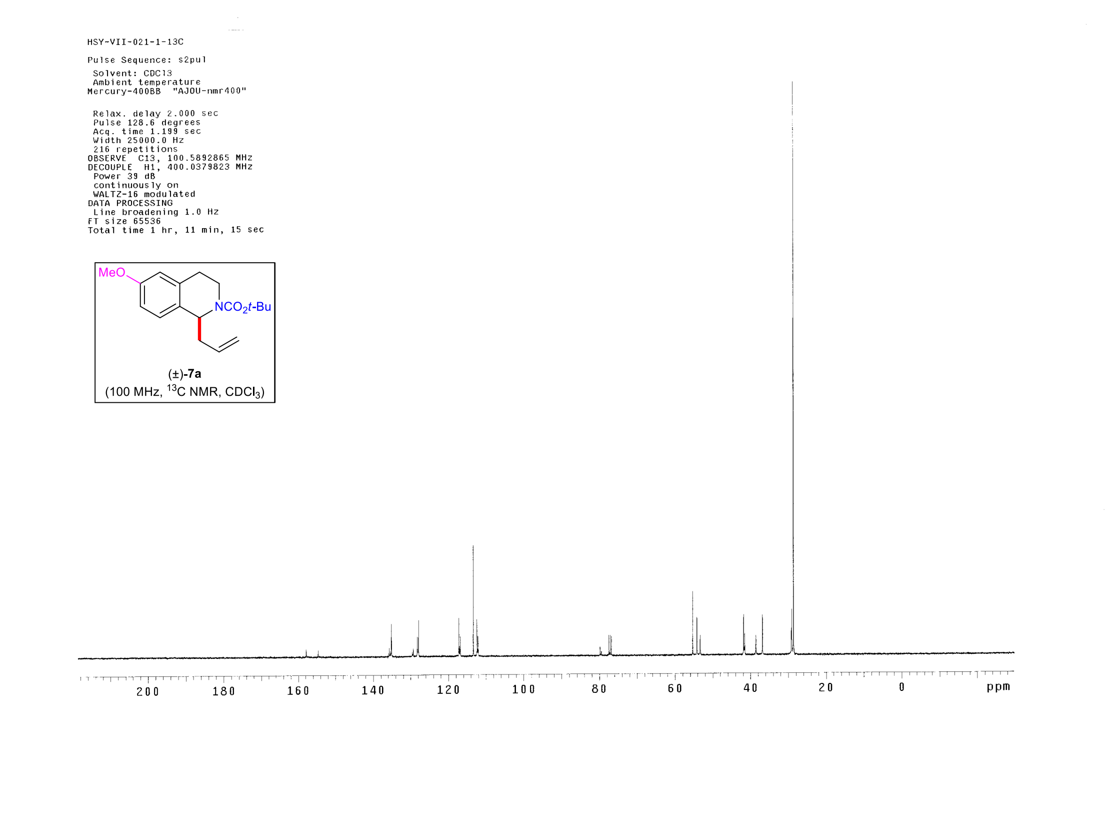

(±)-*tert*-Butyl 1-Allyl-7-methoxy-3,4-dihydroisoquinoline-2(1*H*)-carboxylate (**7b**)

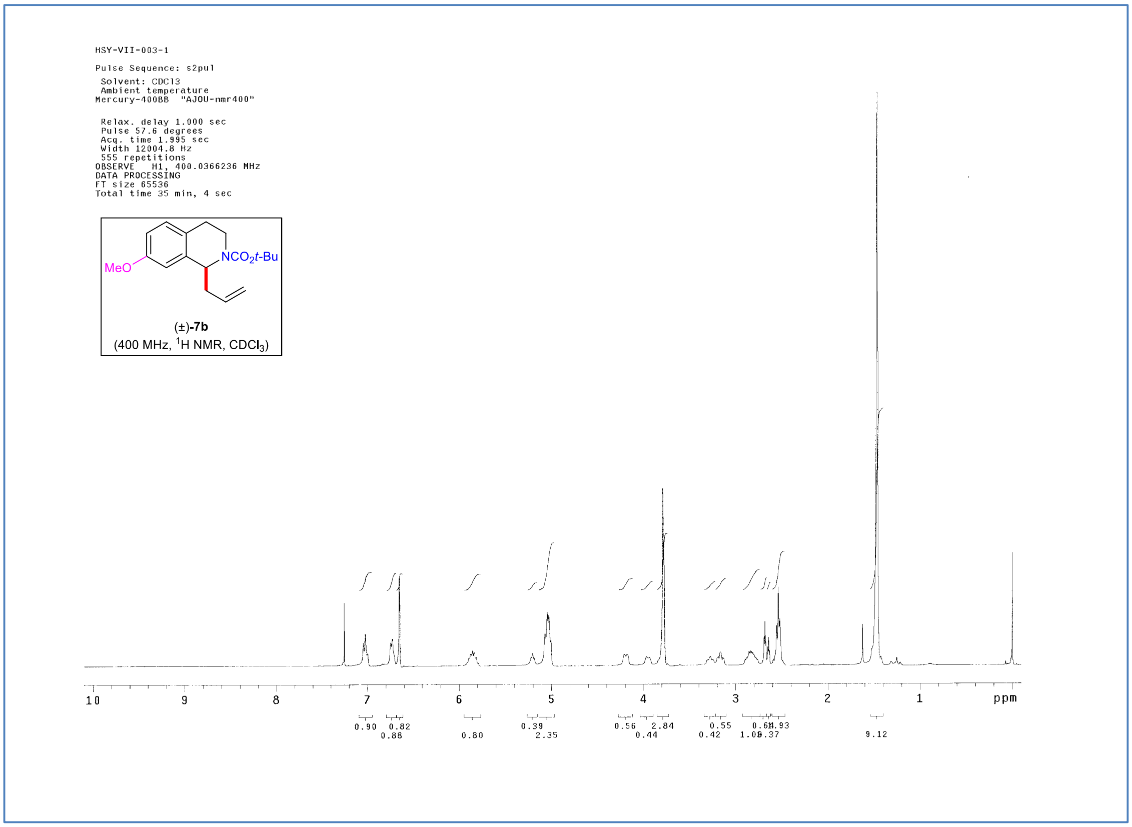

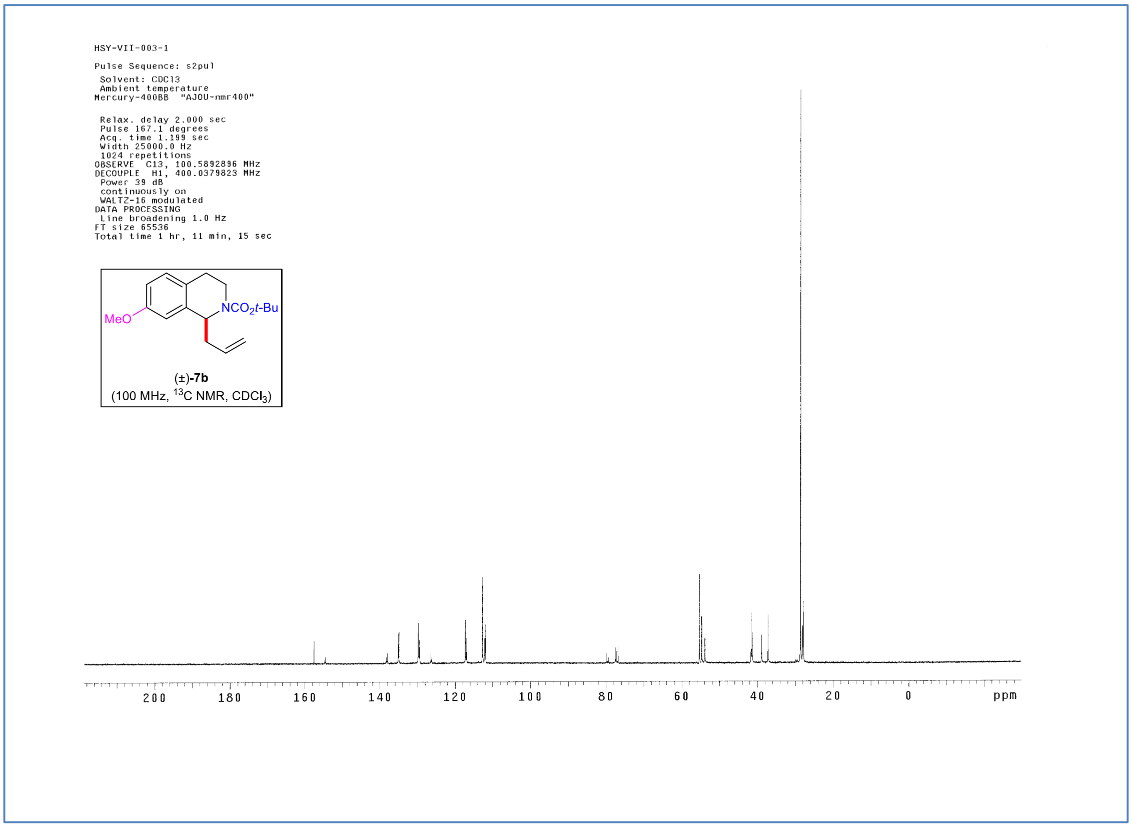

(±)-*tert*-Butyl 1-Allyl-6,8-dimethoxy-3,4-dihydroisoquinoline-2(1*H*)-carboxylate (**7c**)

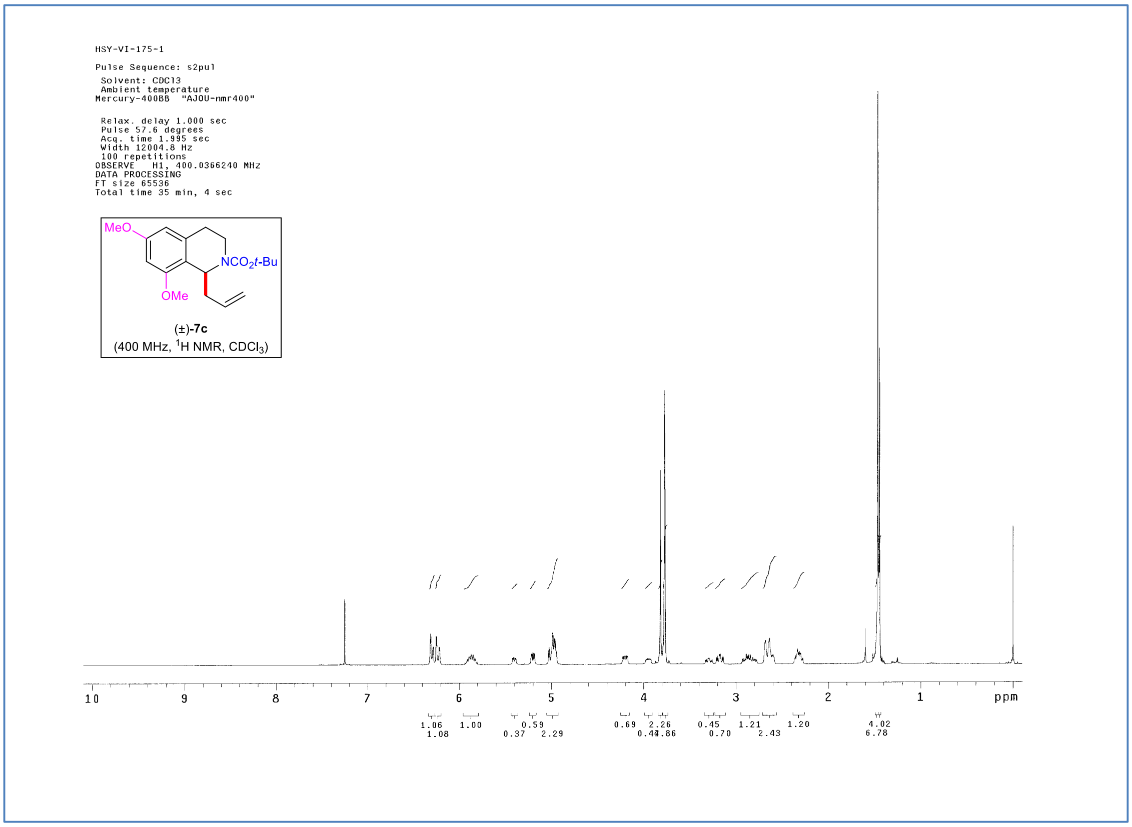

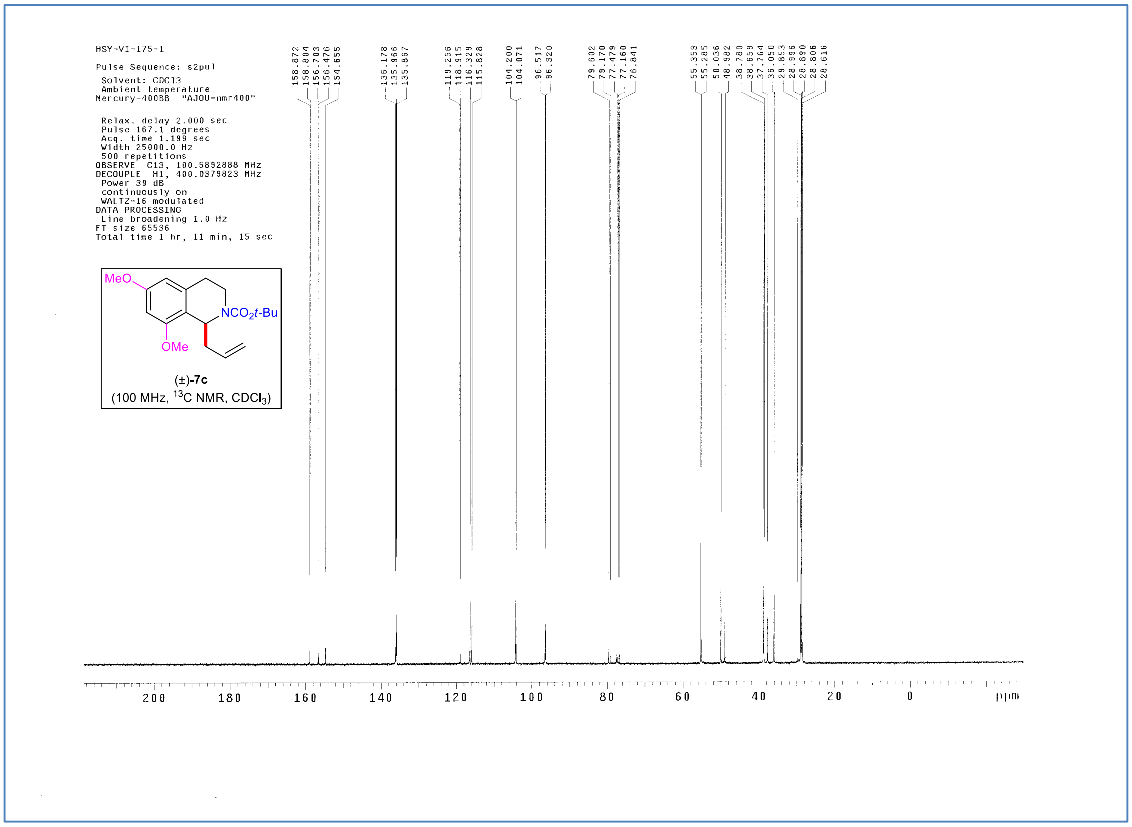

(±)-*tert*-Butyl 1-Allyl-7-fluoro-3,4-dihydroisoquinoline-2(1*H*)-carboxylate (**7d**)

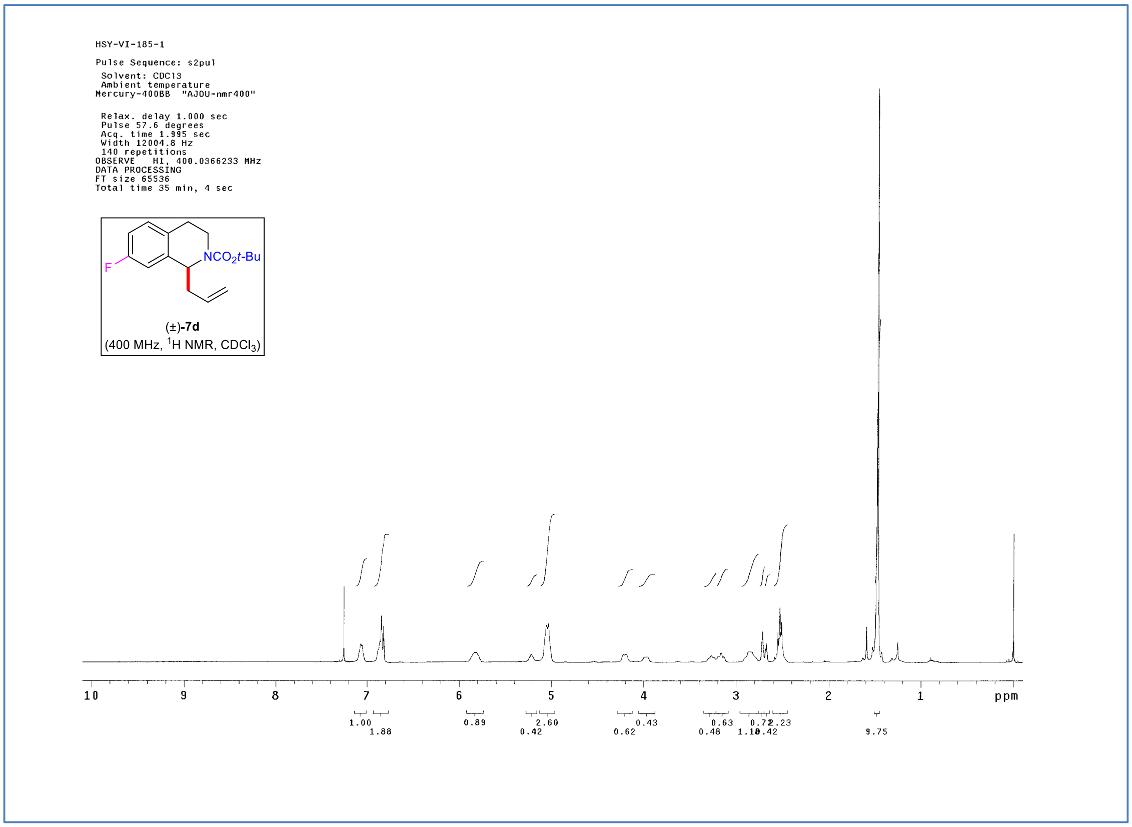

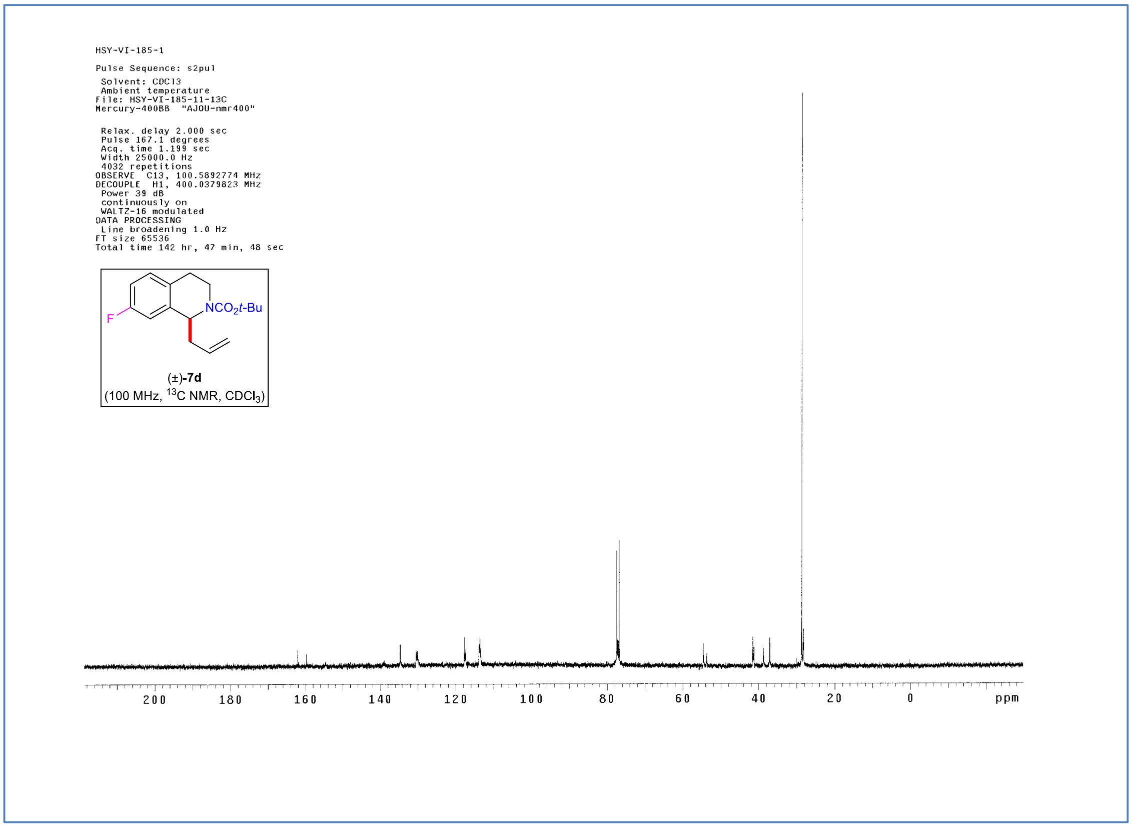

(±)-*tert*-Butyl 1-Allyl-7-bromo-3,4-dihydroisoquinoline-2(1*H*)-carboxylate (**7e**)

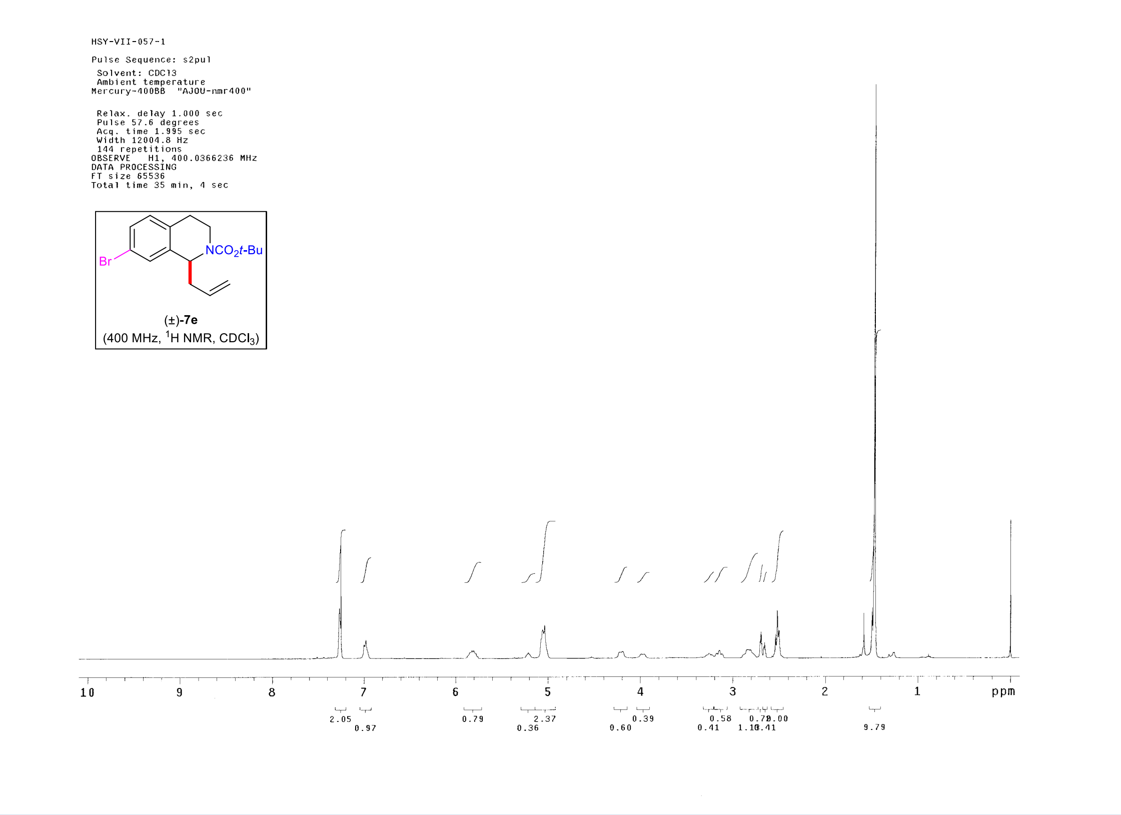

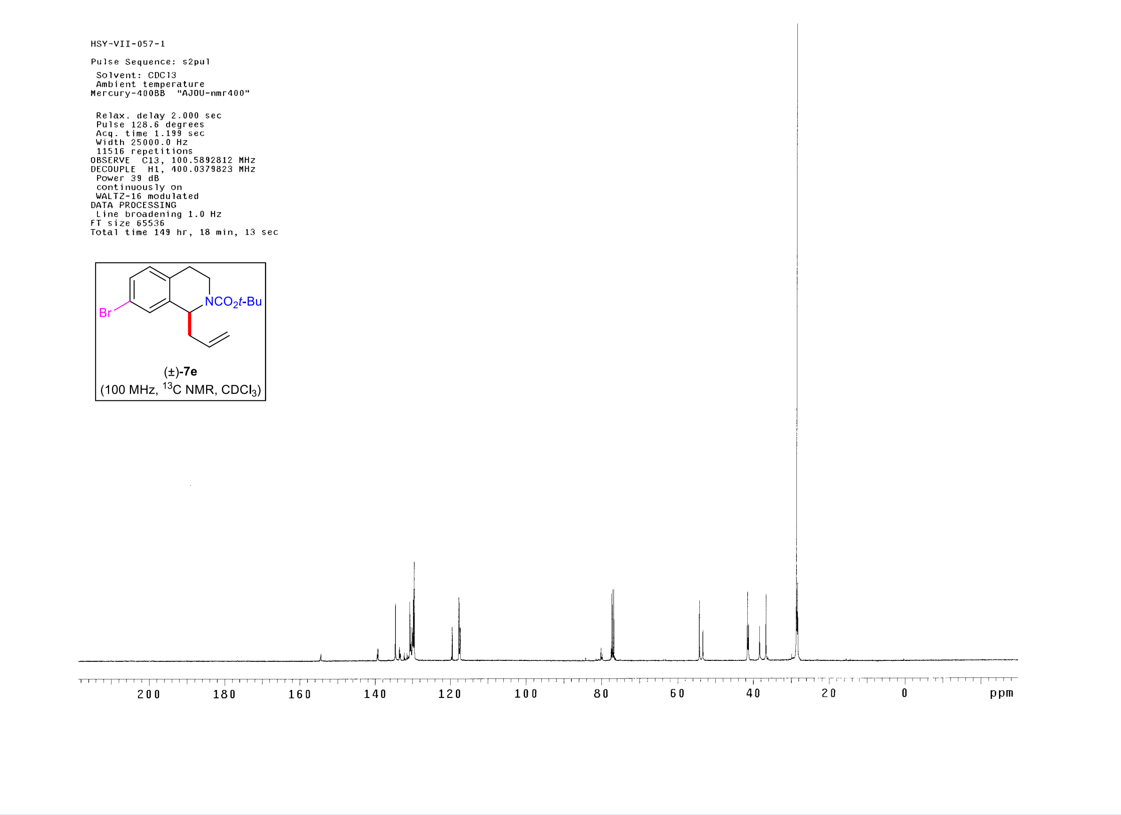

(±)-*tert*-Butyl 1-Allyl-3,4-dihydroisoquinoline-2(1*H*)-carboxylate (**7f**)

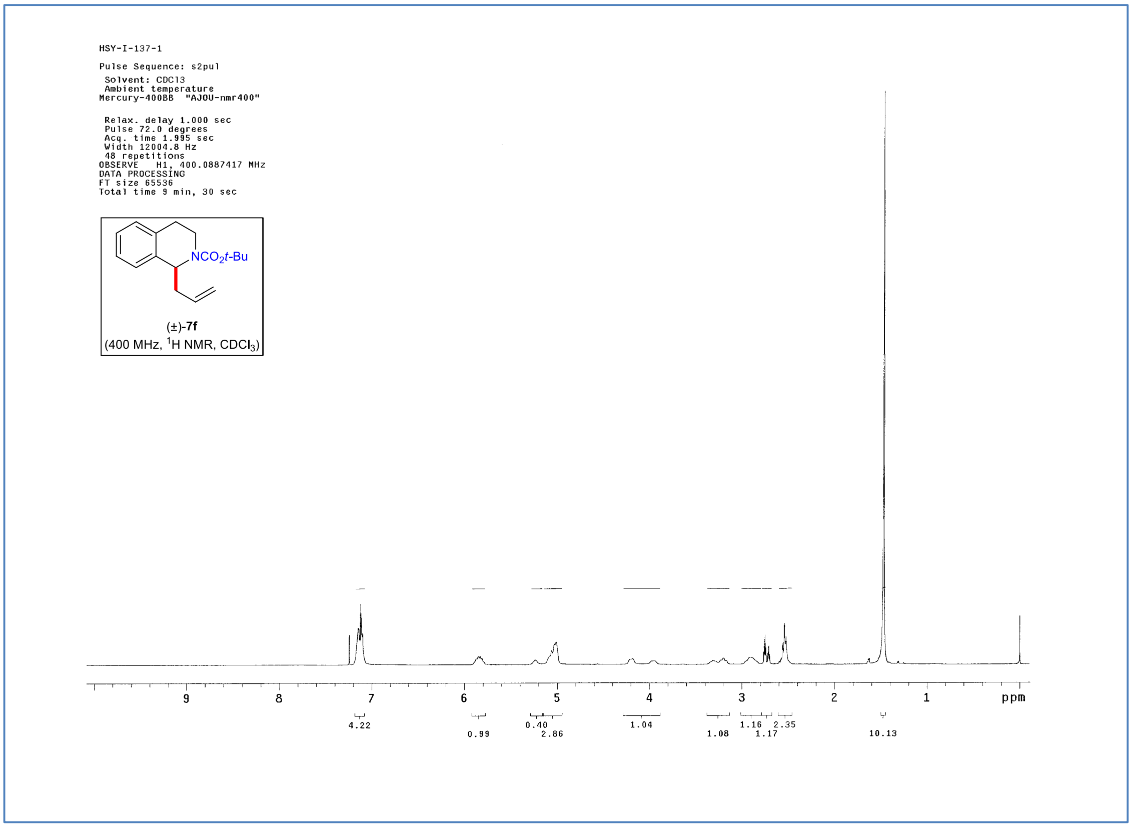

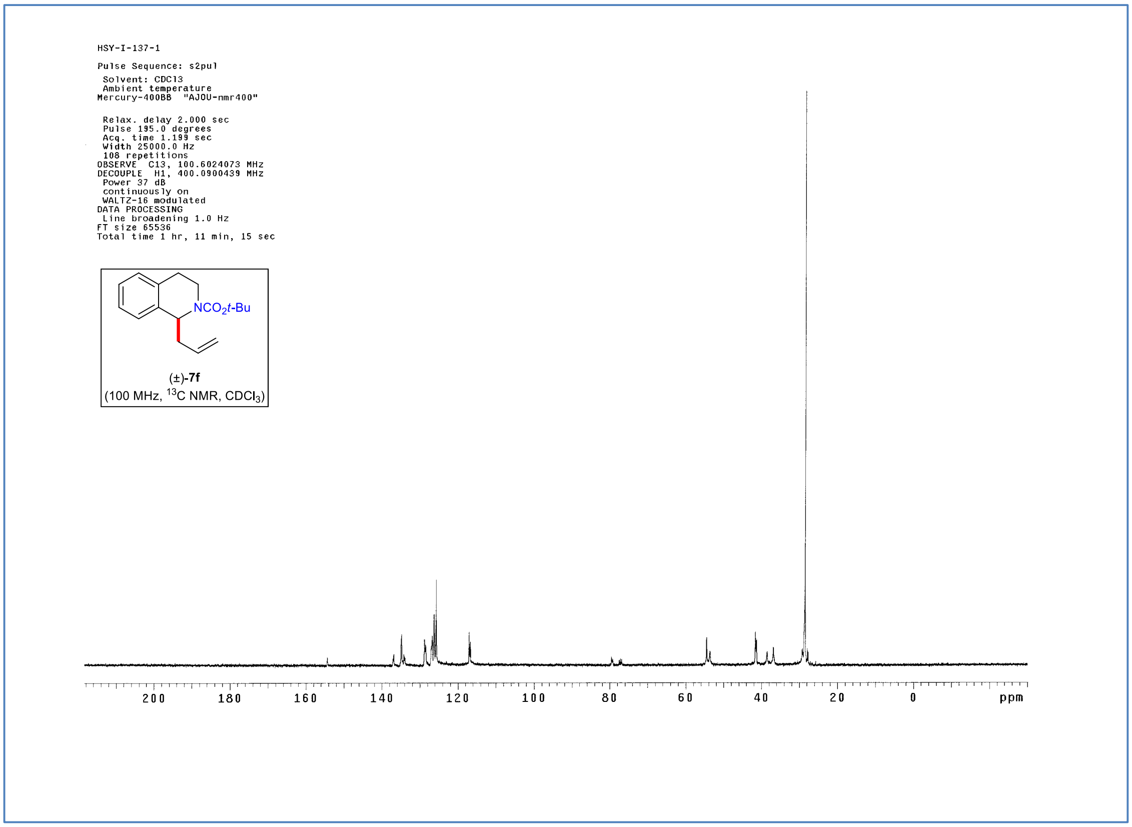

(±)-1-Allyl-6,7-dimethoxy-1,2,3,4-tetrahydroisoquinoline (**8**)

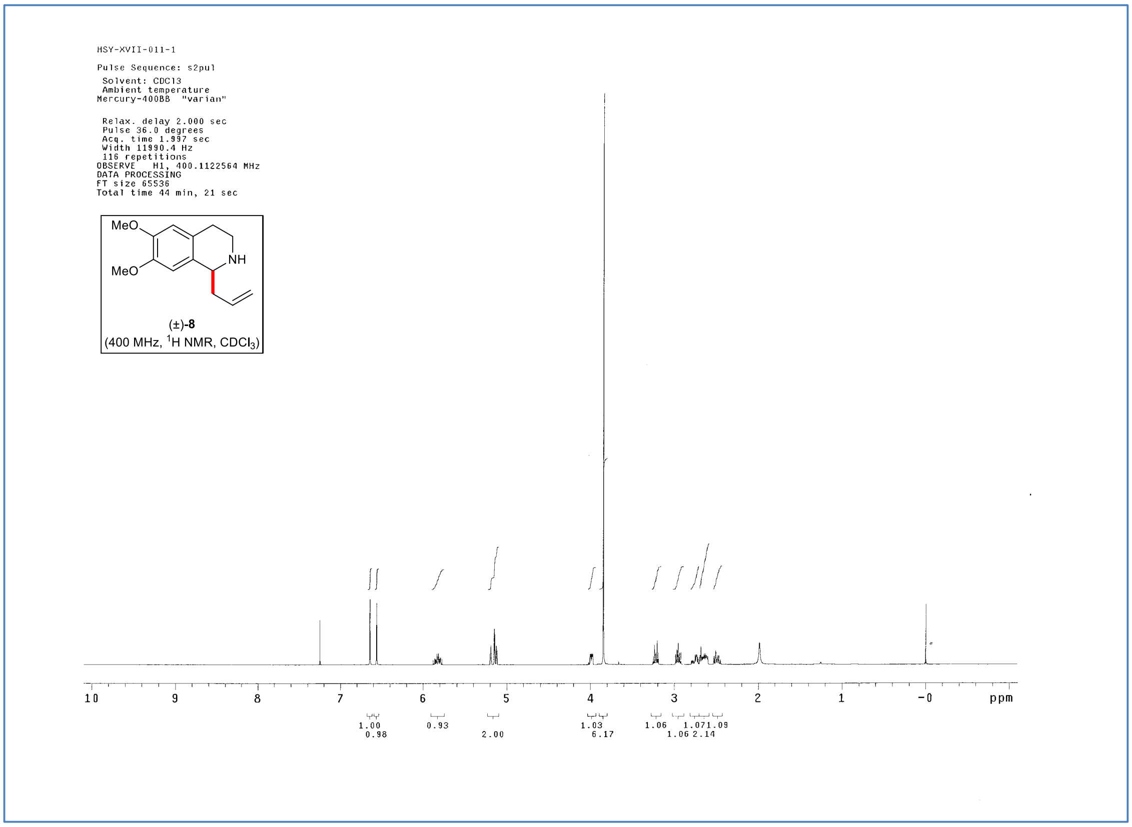

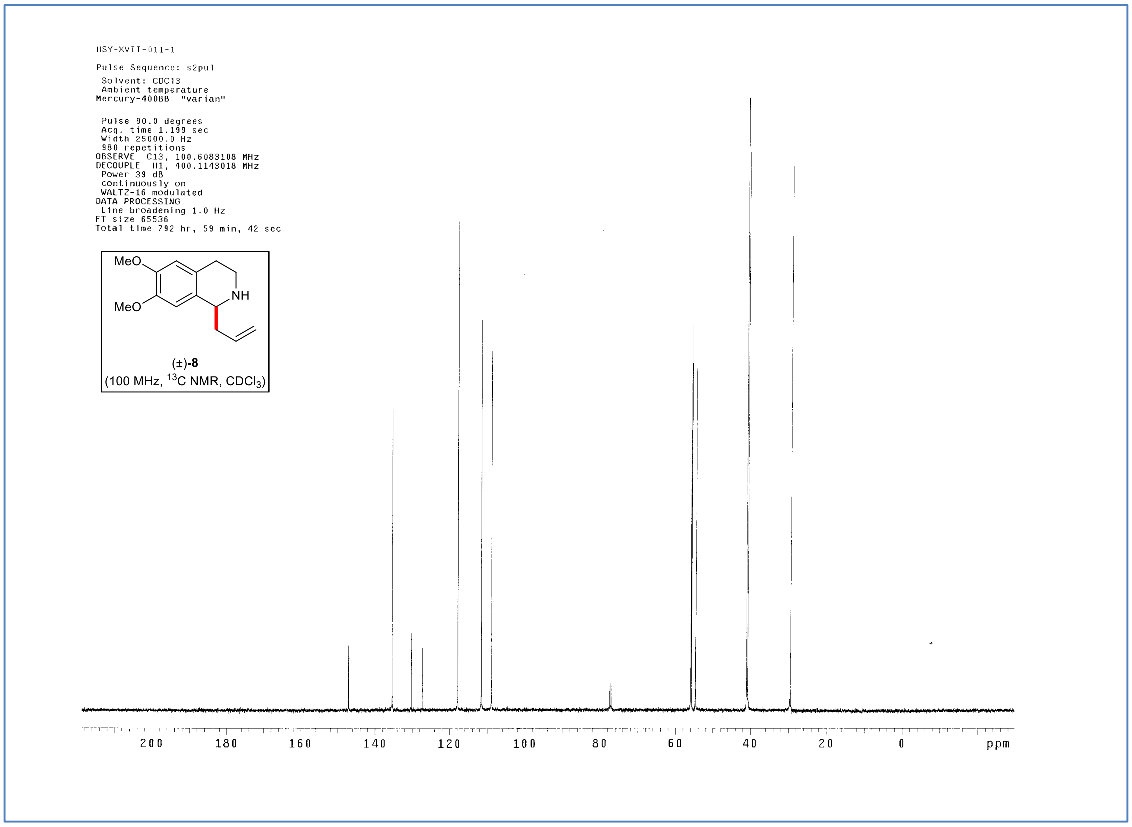

(±)-(*E*)-Benzyl 6,7-Dimethoxy-1-(4-methoxy-4-oxobut-2-en-1-yl)-3,4-dihydroisoquinoline-2(1*H*)-carboxylate (**9**)

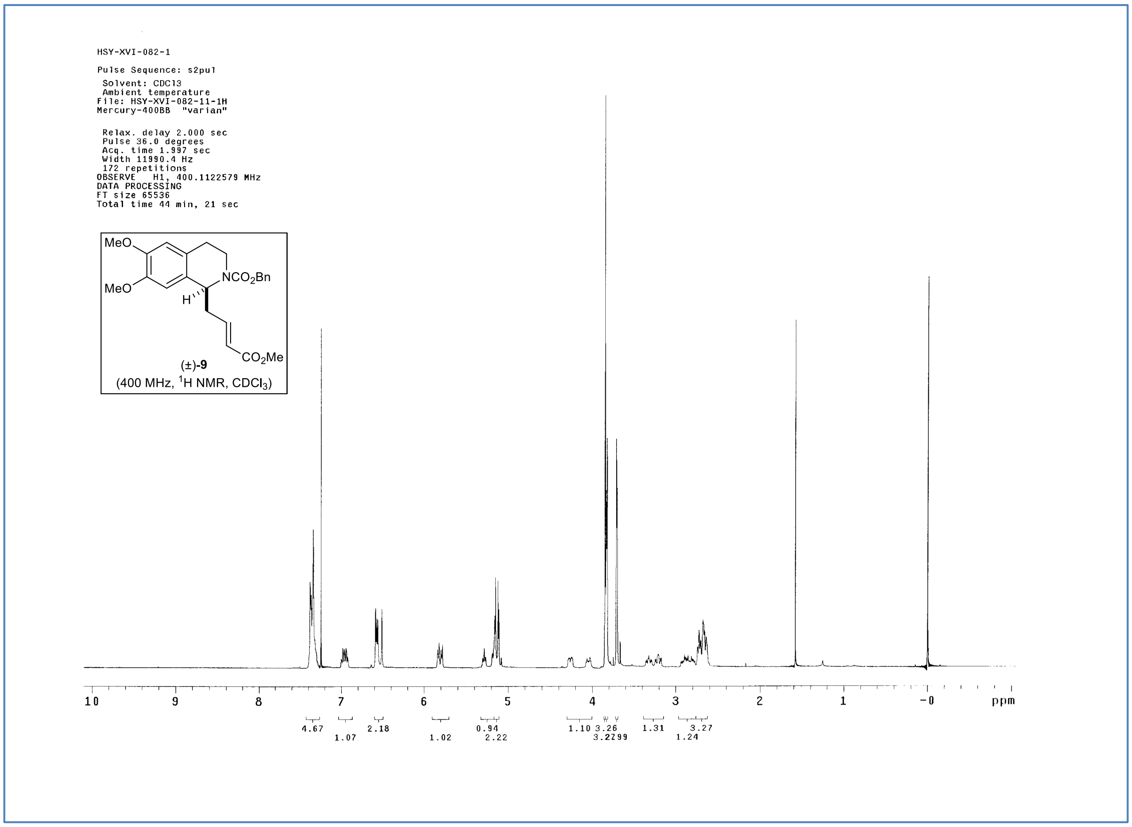

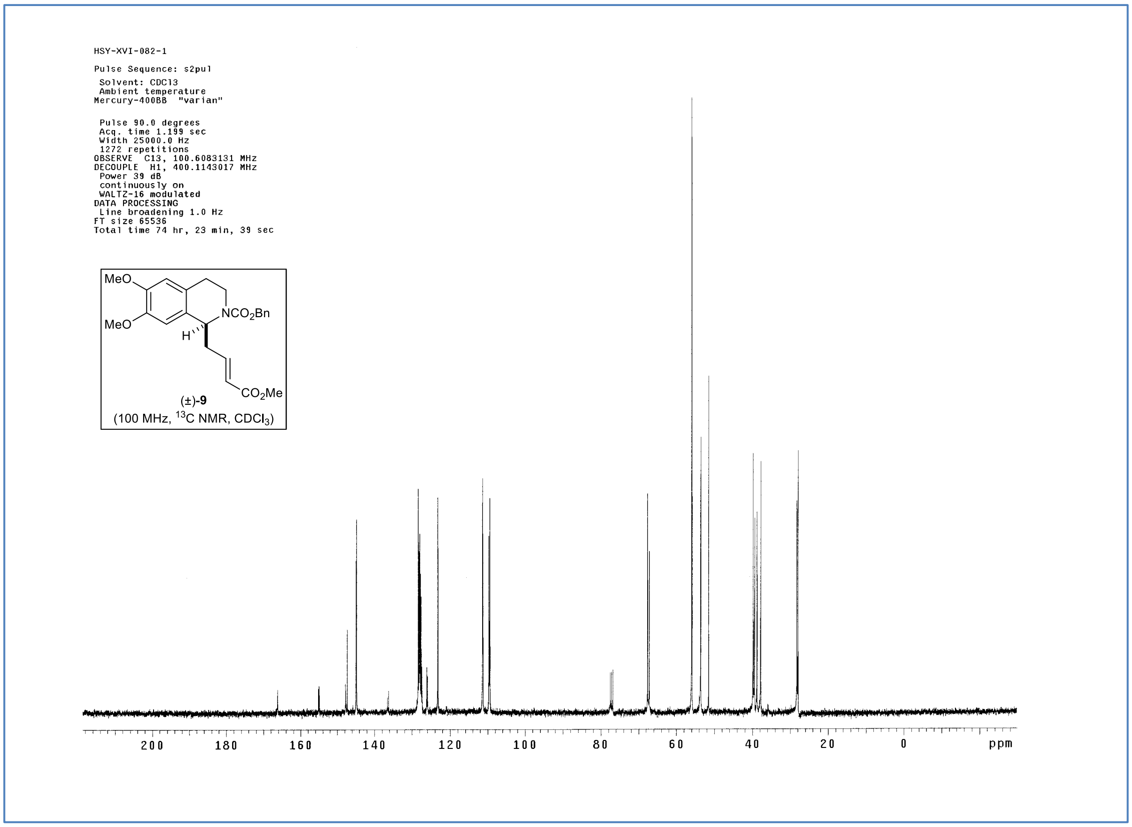

(±)-9,10-Dimethoxy-2,3,6,7-tetrahydro-1*H*-pyrido[2,1-*a*]isoquinolin-4(11b*H*)-one (**10**)

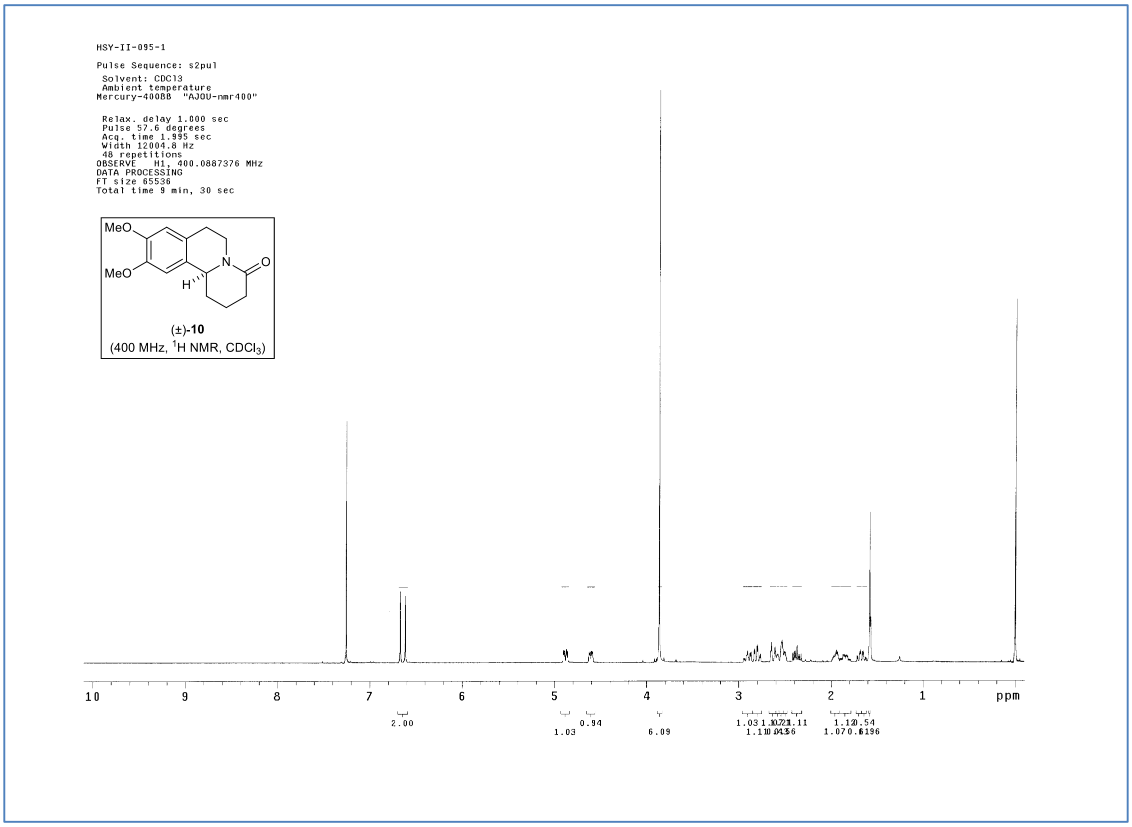

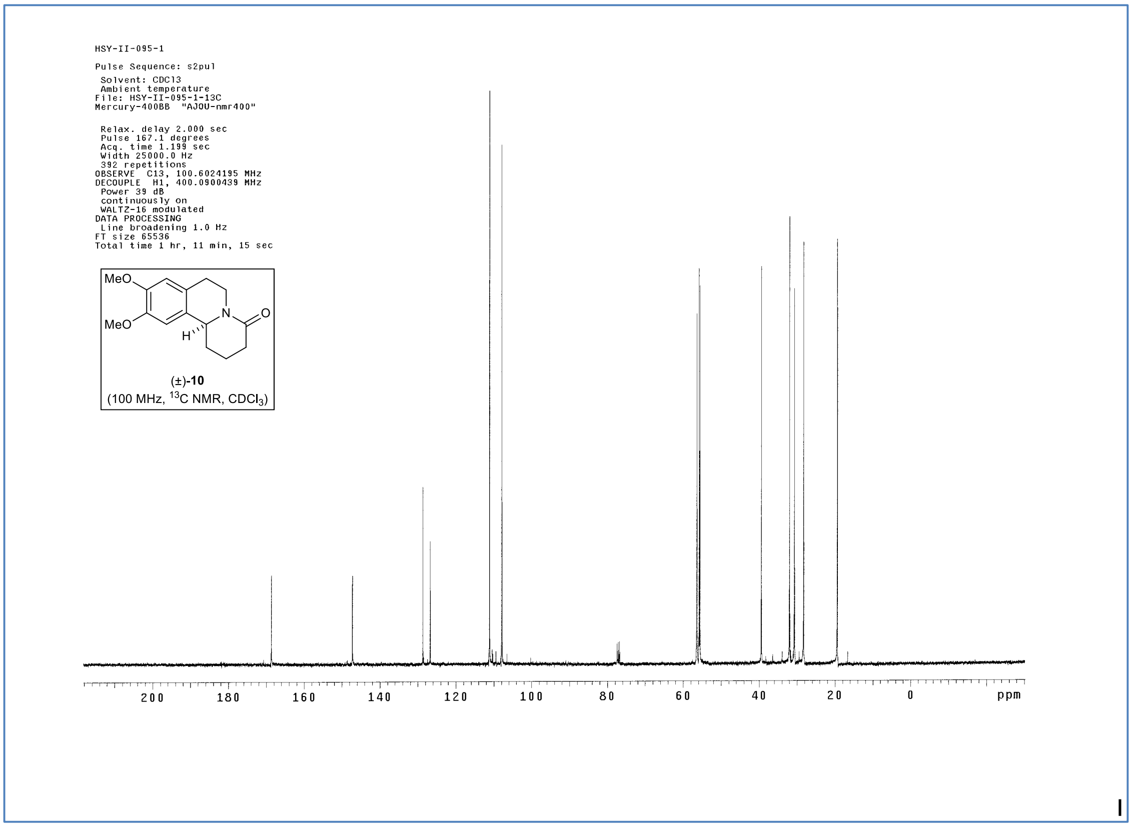

(±)-9,10-Dimethoxy-2,3,4,6,7,11*b*-hexahydro-1*H*-pyrido[2,1-*a*]isoquinoline (**11**)

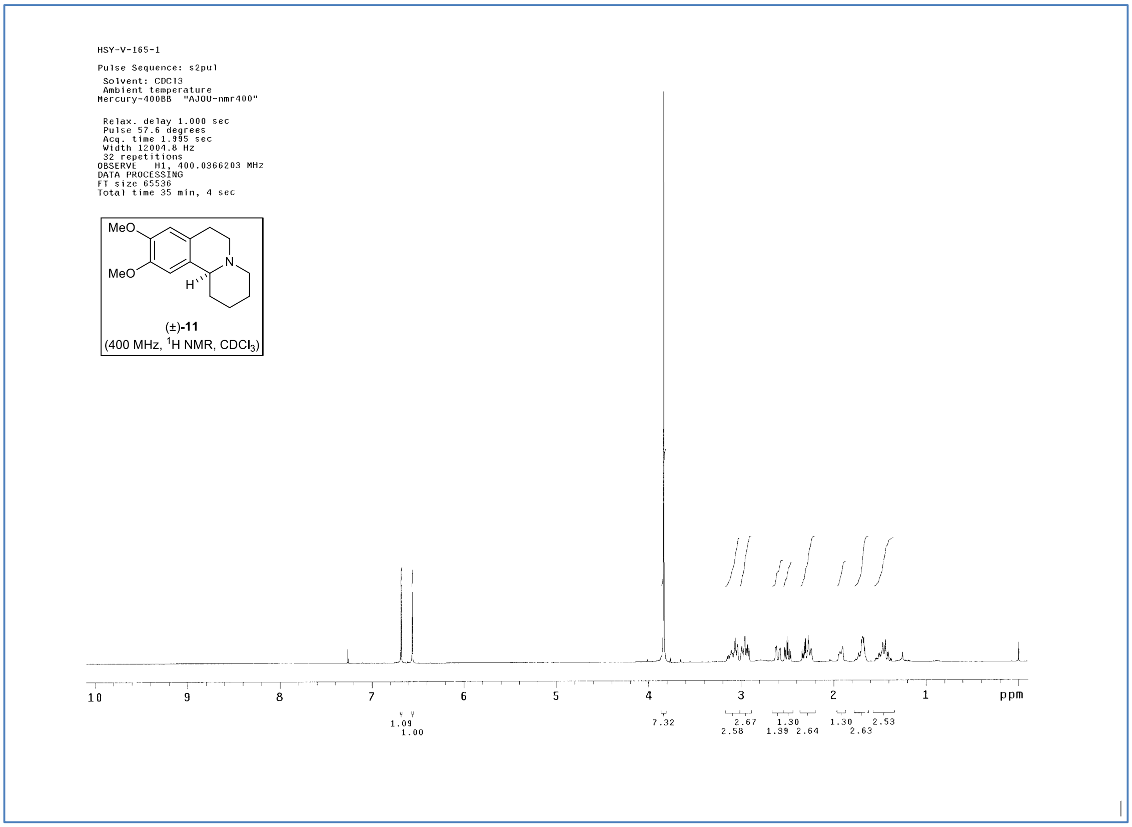

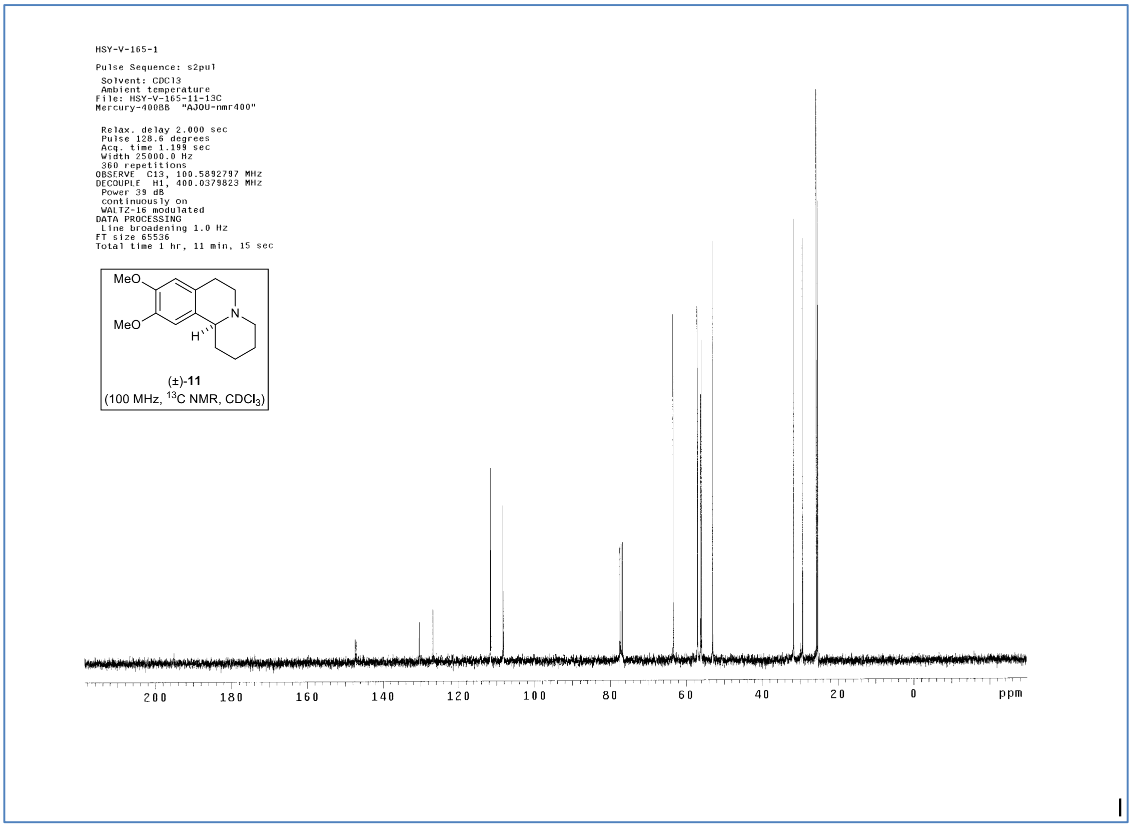

Comparison of ^{13}C NMR Data for (±)-Benzo[*a*]quinazoline (**10**)

Lee (Synthetic) (100MHz, $CDCl_3$)	Drabowicz[1] (Synthetic) (125MHz, $CDCl_3$)	Coldham[2] (Synthetic) (100MHz, $CDCl_3$)
147.3	147.38	147.3
147.1	147.14	147.1
130.4	130.51	130.2
126.8	126.83	126.6
111.6	111.52	111.4
108.3	108.22	108.1
63.4	63.28	63.2
57.0	56.94	56.9
56.2	56.00	56.0
56.0	55.85	55.8
53.0	52.93	52.8
31.7	31.58	31.5
29.3	29.17	29.0
25.7	25.52	25.4
25.3	25.15	25.0

1. Szawkalo, J.; Czarnocki, S. J.; Zawadzka, A.; Wojtasiewicz, K.; Leniewski, A.; Maurin, J. K.; Czarnocki, Z.; Drabowicz, J. *Tetrahedron: Asymmetry* **2007**, *18*, 406–413.
2. Talk, R. A.; Duperray, A.; Li, X.; Coldham, I. *Org. Biomol. Chem.* **2016**, *14*, 4908–4917.
